# Material-architecture-manufacture integrated strategy for piezoelectric metamaterials with mechanical protection and self-powered sensing

**DOI:** 10.1126/sciadv.aeg1744

**Published:** 2026-09-25

**Authors:** Zhicheng Wang, Xiaozhou Xin, Jingfei Wang, Liwu Liu, Yanju Liu, Jinsong Leng

**Affiliations:** ^1^ Department of Astronautical Science and Mechanics, Harbin Institute of Technology, Harbin 150001, People’s Republic of China.; ^2^ Suzhou Research Institute, Harbin Institute of Technology, Suzhou 215100, People’s Republic of China.; ^3^ Centre for Composite Materials and Structures, Harbin Institute of Technology, Harbin 150080, People’s Republic of China.

## Abstract

The increasingly complex tasks of intelligent equipment have shifted the demand for structural materials from mere lightweight protection to multifunction integration, i.e., high recoverable energy absorption and accurate monitoring. Herein, we presented a material-architecture-manufacture integrated strategy for piezoelectric metamaterials to simultaneously achieve mechanical protection and self-powered sensing. Specifically, an intercalation heterostructure of silane-modified barium titanate (K-BTO)/MXene was constructed to improve the interfacial polarization effect, and an innovative electric field–assisted 4D printing technology could effectively induce the in situ poling of K-BTO. Furthermore, the Kagome lattice–inspired metamaterial (k-CAH) exhibited superior specific energy absorption (0.18 joules per gram), resulting from compression-bend-torsion coordination and multidirection coupling mechanisms. The multimode coupling deformation and local strain amplification mechanism, induced by the geometry design, substantially improved output capacities through activating various piezoelectric response modes at low frequency. With the material-architecture-manufacture synergy, $g_{33}^{\text{eff}}$ reached up to 1.712 volt-meters per newton. The developed piezoelectric metamaterial system exhibited accurate self-powered sensing of real-time impact and high-efficiency energy harvesting under microvibration conditions, promising for the next-generation smart structural materials.

## INTRODUCTION

Intelligent equipment plays a key role in modern mechanical engineering, indispensable for advanced technology applications, from autonomous robotic platforms (*1*, *2*) to sophisticated aerospace systems (*3*–*5*). The escalating needs for complicated tasks have shifted the performance requirement of equipment, from foundationally mechanical protection to incorporating a high integration of sensing, actuation and adaptive control (*6*, *7*). This evolution imposes increasingly stringent demands on the high-performance structural materials as an all-in-one backbone of intelligent equipment. It is imperative for structural materials to be not only lightweight (*8*) and high damage tolerant (*9*) but also combining conflicting mechanical properties (e.g., high strength and large recoverable strain) for energy absorption (*10*, *11*). Nowadays, it has attracted increasing interests for further integrating morphing (*12*) and self-sensing capacities (*13*) into structural materials. Nonetheless, it remains a formidable challenge for integrating these optimizations to achieve synergistic mechanical protection and self-sensing in modern structural materials.

Piezoelectric materials are capable of reversible conversion between mechanical strain and electrical potential due to the unique intrinsic noncentrosymmetric structure (*14*, *15*), which are promising for introducing self-sensing function into structural materials. These materials have been widely used into precision actuators (*16*), manipulators (*17*), energy harvesters (*18*), and sensors (*19*, *20*) for state-of-art autonomous systems. Yet, commonly used piezoelectric materials [i.e., lead zirconate titanate (PZT), zinc oxide (ZnO), and barium titanate (BTO)] are susceptible to crack initiation and propagation under long-time mechanical load due to the inherent brittleness and rigidity, limiting the application in structural components (*21*, *22*). Meanwhile, existing piezoelectric materials can mainly response to the external load along the normal or shear direction, and the single-mode energy conversion severely hinders the harvesting efficiency and sensing accuracy. Thus far, researchers mainly focus on material optimization methods [i.e., anion doping (*23*), defect engineering (*24*), and self-assembly (*25*)] to modulate crystalline phase structure and thus boost the mechanical and output performances of piezoelectric materials, which are high-cost materials and require complex procedures.

Mechanical metamaterials, known as architected materials, exhibit exotic properties compared to conventional materials, including auxetic (*26*), negative stiffness (*27*), and elastic wave bandgap (*28*). These promising properties have attracted extensive attention in tailoring the mechanical performances of structural materials for smart sensors and actuators (*29*, *30*). Different from natural materials whose properties were primarily derived from intrinsic composition, the functionalities of architected materials rely on unit structures and topology strategy, which offers a brand-new design paradigm for high-performance structural materials (*31*, *32*). Additive manufacturing technology, in a layer-by-layer deposition manner, has advanced the development of architected materials, offering unprecedented design freedom for complex three-dimensional (3D) geometries and intricate microstructures (*33*–*35*). Recently, additive-manufactured architected materials have garnered increasing attention in the field of piezoelectricity, and the architecture optimization can effectively improve stress distribution and activate various electromechanical conversion modes (*36*, *37*), emerging an ideal approach for piezoelectric structural material. Despite these innovations, collaborative combination of material, architecture, and manufacturing remains challenging to design piezoelectric metamaterials, integrating high-efficiency energy absorption and high-accuracy sensing capacity.

**Fig. 1.**
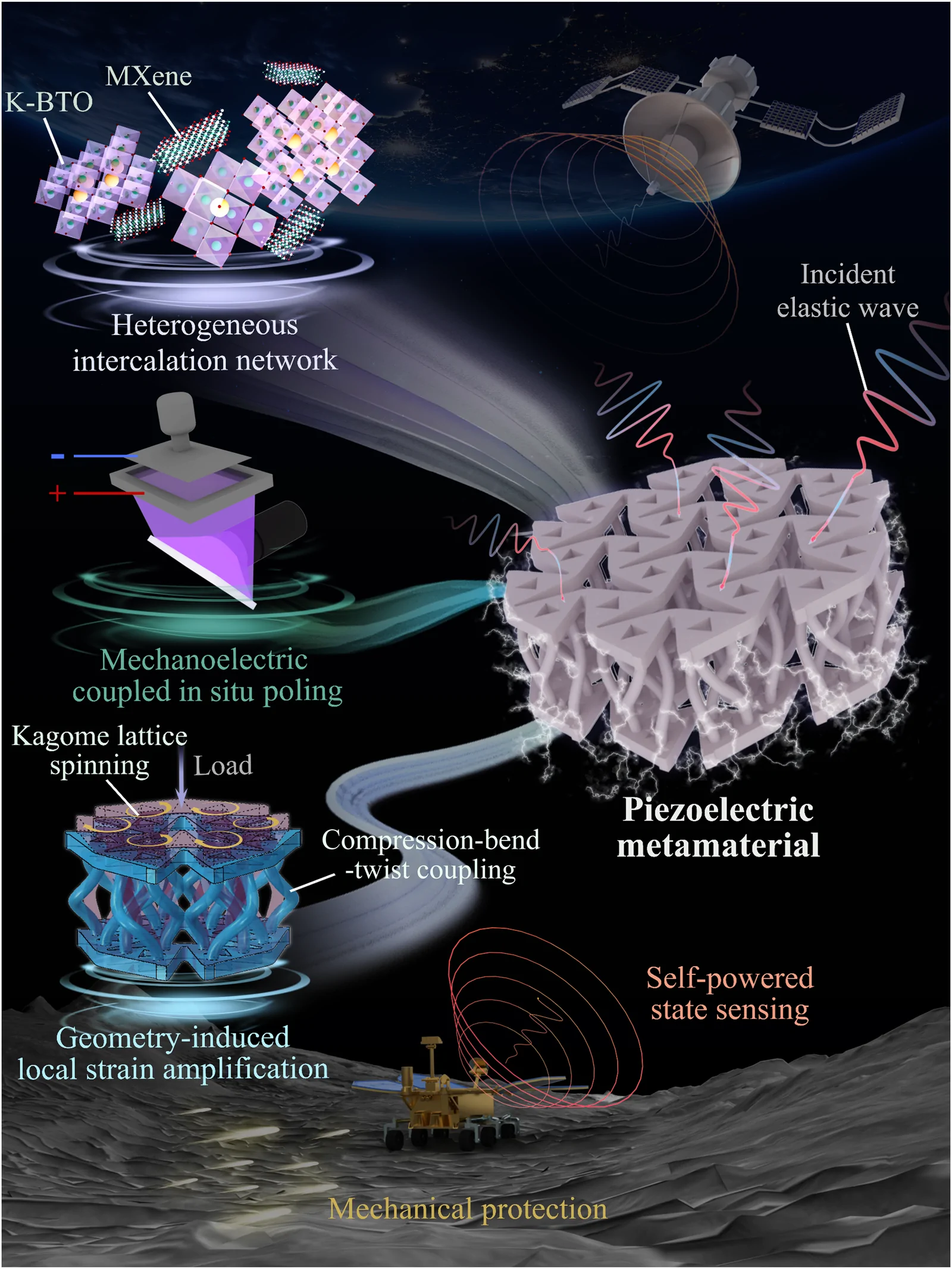
Schematic diagram of the material-architecture-manufacture integrated strategy for piezoelectric metamaterials with mechanical protection and self-powered sensing. The designed piezoelectric metamaterial achieves an unprecedented integration of robust mechanical protection and self-powered state perception, fundamentally derived from trilevel synergistic optimization: (i) enhanced interfacial polarization within the hybrid silane-coated tetragonal crystalline barium titanate (K-BTO)/MXene heterogeneous network (material level), (ii) geometry-induced local strain amplification and multimode coupling deformation (architecture level), and (iii) in situ poling of piezoelectric particles during the multifield-coupled 4D printing process (manufacture level). By seamlessly converting incident low-frequency elastic waves into electricity while attenuating broad-band vibrations, this metamaterial exhibits immense potential as a smart structural component for the next-generation aerospace equipment.

Herein, we presented a novel material-architecture-manufacture integrated strategy for piezoelectric metamaterials, capable of large recoverable energy absorption and excellent self-sensing, for smart structural materials (Fig. 1). For material optimization, we prepared a piezoelectric composite containing an intercalation heterostructure of silane-modified barium titanate (K-BTO) nanoparticles and MXene nanosheets. Through tailoring the component ratio, the piezoelectric coefficient *d*_33_ reached 18.5 pC/N. Moreover, we innovatively developed an electric field–assisted 4D printing method to manufacture piezoelectric metamaterials with superior output capacity and reconfigurability. The synergistic effect of electric and mechanical fields induced in situ poling of piezoelectric phase K-BTO and orientation of reinforcement phase MXene, leading to the improvement of piezoelectric performance (up to 41.5 pC/N in *d*_33_ and 0.763 V·m/N in *g*_33_). For architecture design, we proposed an approach to a chiral helix-arch metacell, integrating prebuckling into the origin geometry and achieving compression-bend-torsion coordinated deformation. The metacells were subsequently assembled following the topology structure inspired by Kagome lattice, and the metamaterial exhibited excellent energy absorption (0.18 J/g) and large recoverable deformation (40%). In addition, the simultaneous activation of various piezoelectric response modes and local strain amplification mechanism lead to a higher effective piezoelectric coefficients, substantially improving the output capacities of metamaterials. This overcomes the low-frequency electromechanical transduction barrier through geometry-driven kinematic amplification rather than frequency-dependent resonance, thereby unlocking unprecedented energy harvest efficiency in a broader frequency domain. Furthermore, the protection system, embedded with 4D-printed piezoelectric metamaterial and microcontroller unit (MCU), can not only sense the real-time impact without external supply but also harvest the vibration energy, potentially for application in aerospace equipment, e.g., satellites and rovers. The developed piezoelectric metamaterial is a promising candidate for smart structural materials, integrating mechanical buffering, energy absorption, self-powered sensing and energy recycling, in next-generation intelligent equipment.

## RESULTS

### Composite preparation and characterizations

**Fig. 2.**
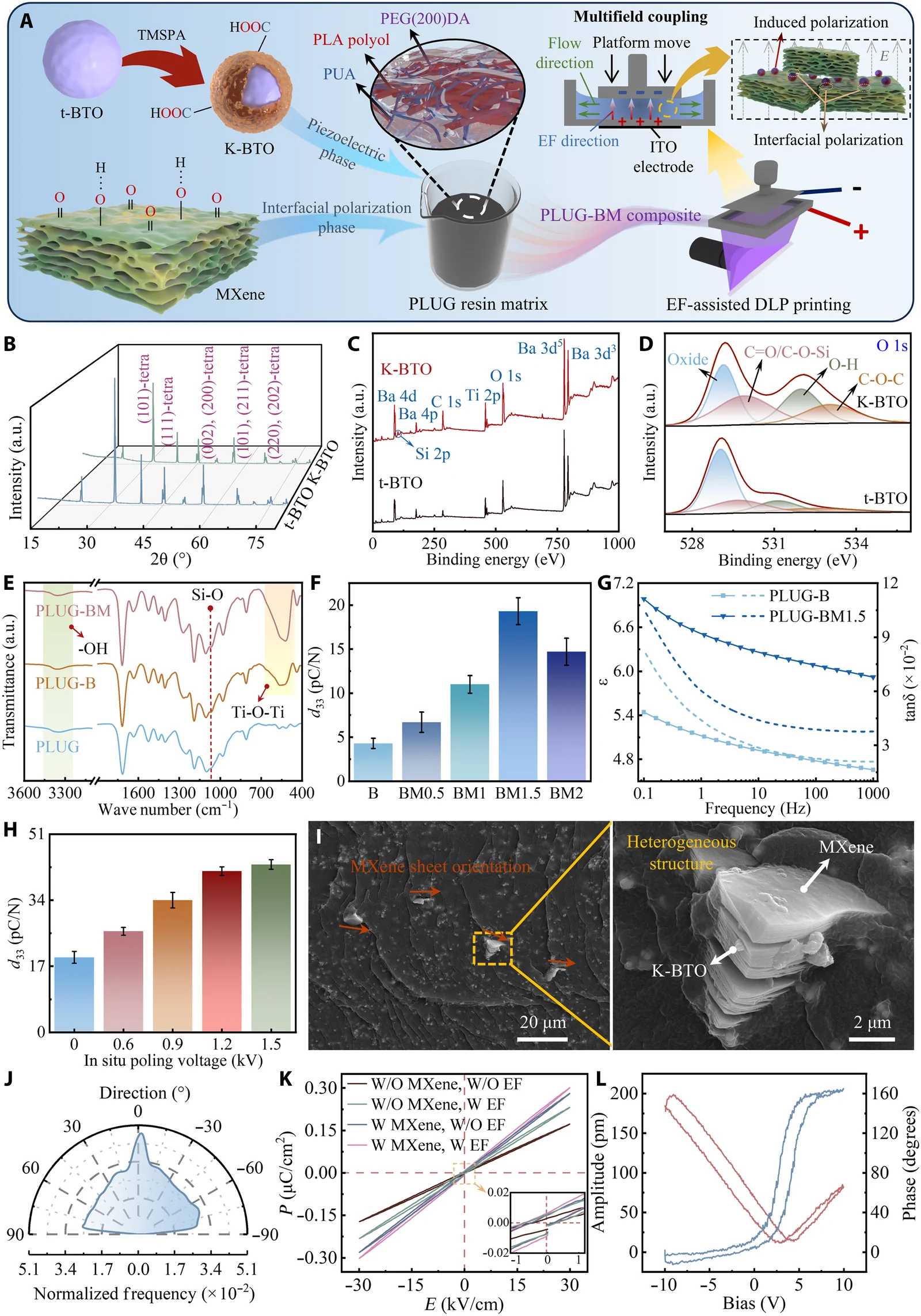
Preparation and characterization of the composite with the heterogeneous intercalation microstructure (PLUG-BM). (**A**) Composition and preparation process of the piezoelectric structure, where the intercalation heterostructure of K-BTO and MXene forms under the coupling effect of flow force and electric field. (**B**) XRD, (**C**) XPS, and (**D**) O 1s spectra of t-BTO and silane-modified barium titanate (K-BTO). a.u., arbitrary units. (**E**) FTIR spectra of polymer matrix (PLUG), composite with K-BTO (PLUG-B) and PLUG-BM. (**F**) Comparison of the piezoelectric constant (*d*_33_) with different MXene contents. (**G**) Real dielectric constant (ε′) and dielectric loss (tanδ) of the PLUG-B and PLUG-BM1.5. (**H**) Comparison of the piezoelectric constant (*d*_33_) with different in situ poling electric field strengths. (**I**) Cross-sectional morphology and (**J**) orientation distribution of PLUG-BM1.5 printed with an in situ electric field (EF) of 1.2 kV/cm. (**K**) *P*-*E* loops of piezoelectric composites with different components and treatments. (**L**) PFM amplitude and phase curves of PLUG-BM1.5 printed with an in situ electric field of 1.2 kV/cm.

The piezoelectric slurry was composed with K-BTO, MXene, and polymer matrix (PLUG), consisting polylactic acid (PLA) polyol, polyurethane acrylate (PUA) and poly(ethylene glycol) 200 diacrylate [PEG(200)DA]. The pure polymer was labeled as PLUG, whereas the PLUG/K-BTO composite was labeled as PLUG-B and PLUG/K-BTO/MXene composites were labeled as PLUG-BM. With the K-BTO content of 10, 20, 30, and 40 wt %, the PLUG-BM composites were labeled to PLUG-B1, PLUG-B2, PLUG-B3, and PLUG-B4, respectively. On the basis of PLUG-B2, the PLUG-BM composites were labeled to PLUG-BM0.5, PLUG-BM1, PLUG-BM1.5, and PLUG-BM2 when the MXene content was 0.5, 1, 1.5, and 2 wt %, respectively. The photocurable slurry was poured into resin tank of the DLP machine for 4D printing, as shown in Fig. 2A. Rather than extrusion-based additive manufacturing techniques limited by nozzle diameters, the high optical resolution of DLP ensures the high-precision fabrication. Compared to the common DLP printing process, this study introduced an electric field–assisted DLP method for piezoelectric metamaterial fabrication. The bottom surface of the tank was attached with a transparent indium tin oxide (ITO) membrane, connecting to the positive electrode of external power supply, whereas the metal platform was connected to the negative electrode. The power supply was toggled with a 5-s interval using a timing circuit (fig. S1). The potential difference between the ITO membrane and platform led to an electric field during the 4D printing process, which induced the in situ poling of K-BTO. Meanwhile, the platform movement made slurry flow along the surface of platform, aligning the MXene nanosheets. As a result of multifield coupling effect, an intercalation heterostructure was constructed, consisting of K-BTO nanoparticles and MXene nanosheets, where the polarized K-BTO particles were well-dispersed between MXene nanosheets. Crucially, the in situ flow-electric synergistic mechanism is inherently dimension-agnostic, facilitating future large-scale production via scale upgrade or modular assembly.

Among various barium titanate crystal phase, the cubic phase features a low piezoelectric coefficient, whereas the tetragonal crystal structure exhibits high piezoelectric performance (*38*, *39*). Because of the ferroelectricity and stability, barium titanate with tetragonal crystalline structure (t-BTO) was chosen as the piezoelectric phase whereas 3-(trimethoxysilyl)propyl methacrylate (TMSPMA) was used to modify the surface of t-BTO (Fig. 2A). Figure 2B indicated that the silane modification had no effect on the crystalline structure of the t-BTO as the x-ray diffraction (XRD) spectrum of K-BTO was similar to that of t-BTO. X-ray photoelectron spectroscopy (XPS) analysis indicated that the silanization reaction successfully introduced TMSPMA molecular bridge on the surface of K-BTO, with the presence of the Si 2p signals at 103.5 eV (*40*) (Fig. 2C). The surficial hydrophilic groups, carbonyl (529.9 eV) and hydroxyl (532 eV), could improve the dispersion of K-BTO nanoparticles in polymer matrix and interaction with MXene nanosheets (*41*, *42*) (Fig. 2D and fig. S2). As shown in fig. S3, the dimension increased slightly after TMSPMA modification, and the scanning electron microscopy (SEM) morphology of the composites exhibited that K-BTO could be well dispersed in the PLUG matrix (fig. S4). The functional group evolution was examined by Fourier transform infrared spectroscopy (FTIR). Absorption peaks at 550, 1050, and 3350 cm^−1^ were attributed to the stretching vibration of Ti─O─Ti, Si─O, and ─OH bonding, respectively (*43*, *44*). There were Si─O bonds in the PLUG-B and PLUG-BM composites with the addition of K-BTO, and the peak for Ti─O─Ti bonding shifted to stronger with the introduction of MXene, indicating the interfacial coupling between MXene and K-BTO (Fig. 2E). Furthermore, the coupling effect reached the highest when K-BTO/MXene ratio reached 30:1.5 (PLUG-BM1.5) as the stretching peak at 550 cm^−1^ was the strongest (fig. S5). The XRD spectra also showed that the coupling effect of MXene had no effect on the crystalline structure of K-BTO nanoparticles (fig. S6).

The energy harvest capacity of piezoelectric materials is determined by the piezoelectric voltage constant (*g*_33_), which represents the electric field generated per unit stress and can be calculated following Eq. 1 (*45*)

$$g_{33}=\frac{d_{33}}{\varepsilon \cdot \varepsilon_{0}}$$ (1)

where *d*_33_, ε, and ε_0_ denoted the piezoelectric charge constant, dielectric constant of the material, and permittivity of free space, respectively. As a result, the piezoelectric material with higher *d*_33_ and lower ε can maximum the energy recycling efficiency, and the recycled energy per area (*E*_*rec*_) could be calculated following Eq. 2

$$E_{\text{rec}}=\frac{1}{2}\cdot d_{33}\cdot g_{33}\cdot \sigma^{2}\cdot t$$ (2)

where σ and *t* represented the applied stress and material thickness, respectively. With the addition of K-BTO, the real dielectric constant (ε′) of PLUG composites exhibited a significant improvement at gigahertz-band frequency, whereas the dielectric loss (*tan*δ) remained low (fig. S7). It was also found that the ε′ of composites increased slightly with the introduction of MXene, which resulted from the construction of an intercalation heterostructure. The *d*_33_ piezoelectric charge constant was further measured at room temperature, and the PLUG-BM1.5 composite exhibited the highest *d*_33_, reaching 18.5 pC/N, which was four times of the PLUG-B (Fig. 2F). This indicated that the heterostructure was optimized when the ratio between K-BTO and MXene reached 30:1.5, consistent with the results of FTIR. To calculate the frequency-consistent *g*_33_ value, the low-frequency ε and *tan*δ of PLUG-BM1.5 and PLUG-B, as a control, were subsequently measured at room temperature, ranging from 0.1 to 1000 Hz. As shown in Fig. 2G, the ε of PLUG-B and PLUG-BM1.5 at 100 Hz, frequency-consistent with *d*_33_, was 4.83 and 6.15, respectively. To optimize the electric field–assisted 4D printing process, the relationship between *d*_33_ of 4D-printed specimens, based on PLUG-BM1.5, and electric field strength was further studied. The result indicated that *d*_33_ increased with the increasing in situ electric field strength, and the improvement turned to weak when electric field reached higher than 1.2 MV/m (Fig. 2H). The specimens for following tests were fabricated by 4D printing assisted with an electric field of 1.2 MV/m, whose *d*_33_ reached 41.5 pC/N. According to Eq. 1, the *g*_33_ of PLUG-BM1.5 prepared via electric field–assisted 4D printing reached 0.763 V·m/N, which was 2.7 times of traditional polyvinylidene difluoride (PVDF) (0.28 V·m/N) and 6.9 times of poly(l-lactic acid) (PLLA) (0.11 V·m/N) (*46*, *47*).

To evaluate the synergy of the heterogeneous intercalation microstructure and in situ poling effect during the printing process, the *g*_33_ of piezoelectric specimens with different components and treatments was compared (Table 1). The postprinting corona poling treatment was carried out as a control group, and the external poling electric field was set to 30 kV/cm as the PLUG-BM1.5 broke down when the electric field reached higher than 35 kV/cm. It was found that the effect of postprinting corona poling was significantly higher than that of in situ poling as the *g*_33_ of the PLUG-B specimen with corona poling (0.625 V·m/N) was more than twice of the group with in situ poling (0.274 V·m/N). In addition, the introduced MXene could slightly improve the *g*_33_ of the piezoelectric composite, from 0.101 to 0.355 V·m/N. It was exciting to find that the *g*_33_ of PLUG-BM1.5 with in situ poling (0.763 V·m/N) surpassed that with postprinting corona poling (0.689 V·m/N), attributing to the improved charge separation and transfer efficiency with the construction of the K-BTO/MXene intercalation heterostructure (*48*). In the intercalation heterostructure network, aligned MXene nanosheets acted as microcapacitors to amplify the local electric field, effectively driving the BTO nanoparticles to the poled state. Meanwhile, the in situ polarization occurs during the photopolymerization process, and the mechanical clamping resistant in liquid resin is much lower than the solid cross-linked resin, significantly decreasing the energy barrier for domain switching (*49*). To directly visualize the intercalation heterostructure and validate the flow-induced alignment mechanism, the cross-sectional morphology of the PLUG-BM1.5 was observed with SEM, and an aligned MXene network was observed (Fig. 2I). Specifically, K-BTO nanoparticles were imbedded in the multilayers of MXene nanosheets, forming a well-dispersed intercalation heterostructure. As shown in Fig. 2J, statistical analysis of the nanosheet orientations revealed that MXene/K-BTO network mainly centered at 0°, which directly and quantitatively proved that the shear stress of flow field during DLP printing effectively aligns the MXene network.

**Table 1.** *g*_33_ (V·m/N) of piezoelectric composites with different components and treatments. W/O, without; W, with; EF, in situ electric field (1.2 MV/m) poling; CP, postprinting corona poling.

	W/O EF	W EF	W CP
**PLUG-B**	0.101	0.274	0.625
**PLUG-BM1.5**	0.355	0.763	0.689

The in situ poling efficiency was fundamentally investigated across different scales. The ferroelectric hysteresis (*P*-*E*) loops exhibited that all composite groups were nearly linear or highly unsaturated curves (Fig. 2K). This results from the severe voltage-divider effect induced by the highly insulating resin matrix, shielding the active piezoelectric phase and preventing the composite from the entirely poling saturation (*50*). However, localized ferroelectric switching was unambiguously verified via piezoresponse force microscopy (PFM). As shown in Fig. 2L, the PLUG-BM1.5, with an in situ poling electric field of 1.2 MV/m, exhibits a butterfly amplitude curve and a highly sharp phase hysteresis loop as well as a near 180° phase switching, upon a 10-V bias voltage. It was crucially demonstrated that, despite that the composite did not reach poling saturation macroscopically, locally ferroelectric domain switching was successfully achieved at the microscale at the heterogeneous interfaces. To rigorously assess the synergistic effect of construction of the heterogeneous intercalation microstructure and in situ poling electric field, a systematic study was performed using PFM amplitude and phase mappings across four groups (fig. S8). Without both MXene and in situ electric field (W/O MXene, W/O EF), the mappings displayed pure background noise. Crucially, applying the in situ electric field to the pure composite (W/O MXene, W EF) or incorporating MXene without poling (W MXene, W/O EF) yields only marginal improvements, maintaining a random phase distribution and negligible amplitude enhancement. A marked transition emerged when material optimization and multifield coupling manufacture were integrated (W MXene, W EF). This group exhibits significantly enhanced piezoelectric amplitude (up to 65 mV) and high phase contrast (exceeding 140°). This proved that the macroscopic piezoelectric enhancement was derived exclusively from the synergy of intercalation heterostructure formation and multifield coupling, where the flow stress aligned MXene nanosheets, as distributed microcapacitors, thereby driving the in situ polarization of K-BTO nanoparticles during the DLP printing process.

### Metacell design and deformation mechanism analysis

**Fig. 3.**
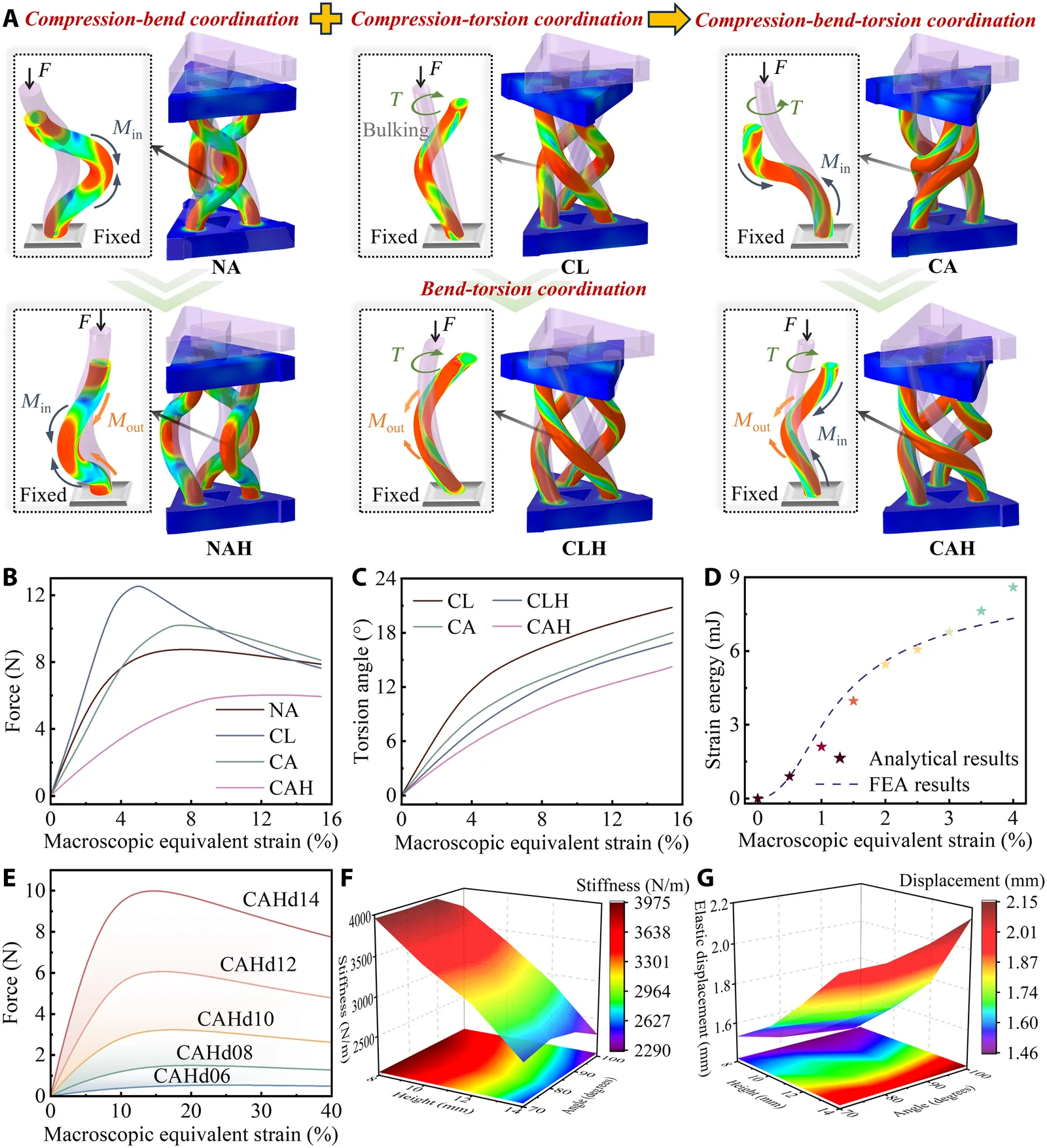
Deformation mode analysis and structure optimization of metacells. (**A**) Schematic diagram of the compression-bend coordination mode of the nonchiral arch metacell (NA), compression-torsion coordination of chiral line metacell (CL), compression-bend-torsion coordination of chiral arch metacell (CA), and out-of-plane bend-torsion coordination of helix metacells (NAH, CLH, and CAH). (**B**) Force-strain and (**C**) torsion angle-strain curves of metacells. (**D**) Comparation between the analytical model and FEA results. (**E**) Force-strain curves of CAH metacells with different rod diameters of 0.6 mm (CAHd06), 0.8 mm (CAHd08), 1 mm (CAHd10), 1.2 mm (CAHd12), and 1.4 mm (CAHd14). (**F**) Stiffness and (**G**) elastic displacement contour plots of CAH metacells with various rod heights and oblique angles (β).

Arch rods have been widely used for buffering in large-scaled load-bearing structure due to the superior stability and high critical buckling load compared to the vertical rods (*51*). Therefore, we first designed a triangled metacell with three arch rods and the theory analysis considered the stage of small compression displacement, where no buckling occurs. The strain energy (*U*_s_) stored in the arched rods, subjected to a vertical compression force (*F*_v_), is mainly attributed to the coordination of compression (*U*_cpr_) and in-plane bending (*U*_in-bend_), as shown in Fig. 3A, and the maximal von Mises stress (σ_v_) occurs at the outer surface of rod ends. The analytical model can be considered as the solution to a doubly clamped rod question, which follows Eqs. 3 and 4

$$U_{s}=U_{\text{cpr}}+U_{\text{bend}}=\frac{E_{m}\pi r^{2}s^{2}}{2L_{0}}+\frac{E_{m}\pi^{5}r^{4}w_{\text{bend}}^{2}}{L_{1}^{3}}=F_{v}\varepsilon$$ (3)

$$K_{\text{eff}}=\frac{F_{\text{rod}}}{\varepsilon }=\frac{E_{m}\pi r^{2}}{L_{0}},\;F_{\text{rod}}=\frac{F_{v}}{3}$$ (4)

where *r*, *L*_0_, *L*_1_, and *E*_m_ represent the radius, initial and shortened length, and Young’s modulus of rods, respectively, and ε represents the compression vertically axial displacement. In addition, *n*, *s*, and *w*_bend_ represent the rod number, compression deformation, and curvature of rods, respectively. The specific analytic model was shown in text S1. As *s* << *w*_bend_, the stored strain energy is mainly attribute to the bend deformation when bend occurs, and the effective stiffness (*K*_eff_) of the arch rod is significantly lower than the vertical rod. However, the uneven distribution of stress leads to the low strength of metacells, which limits the application in practical engineering. Therefore, it is necessary to introduce additional deformation modes for absorbing more energy.

Chiral structure, benefited from the unique compression-torsion coordination, introduces additional twist deformation to store more energy, which substantially improves the energy absorption efficiency (*52*, *53*). The *U*_s_ of chiral metacells is attributed to compression and torsion (*U*_tor_) of the rod (Fig. 3A), which can be considered as Eq. 5

$$U_{s}=U_{\text{cpr}}+U_{\text{tor}}=\frac{E_{m}\pi r^{2}s^{2}}{2L_{0}}+\frac{E_{m}\pi r^{4}\theta^{2}}{8L_{0}\left(1+\nu \right)}$$ (5)

where ν represents the Poisson’s ratio of rods and θ represents the torsion angle of oblique rods. As torsion energy is dominant to the stored energy and part of axial deformation transfers into twist angle, the *K*_eff_ significantly increased due to the decrease in ε, which can be considered as Eq. 6

ε=s∗cosβ (6)

and β represents the oblique angle of rods. In addition, the stress distribution was determined by σ_cpr_ as well as the shear stress induced by the torsion (τ). As τ across the surface of chiral rods is perpendicular to σ_cpr_ and σ_in-bend_, the maximum σ_v_ in chiral metacells follows Eq. 7

$$\begin{array}{c} \sigma_{v}=\sqrt{\sigma_{\text{cpr}}^{2}+3\tau^{2}}\\ \sigma_{\text{cpr}}=\frac{E_{m}s}{L_{0}},\;\tau =\frac{E_{m}\theta r}{2\left(1+\nu \right)L_{0}} \end{array}$$ (7)

The additional twist deformation is capable to significantly improve the energy absorption efficiency through the modulation of stress distribution, but it is prone to induce buckling in the chiral metacells, which may affect the wide application in critical load bearing structures, as depicted in text S2. Therefore, we designed a novel metacell based on arch rods and chiral structure, and further optimize the rod structure into helix to introduce additional out-of-plane bend deformation mode, which could boost the energy absorption efficiency and prevent buckling. The specific analysis process is depicted in text S3. The *U*_s_ can be considered as Eq. 8

$$\begin{array}{c} U_{s}=U_{\text{cpr}}+U_{\text{in}-\text{bend}}+U_{\text{out}-\text{bend}}+U_{\text{tor}}\\ =\frac{E_{m}\pi r^{2}s^{2}}{2L_{0}}+\frac{E_{m}\pi^{5}r^{4}w_{\text{in}-\text{bend}}^{2}}{16L_{1}^{3}}+\frac{E_{m}\pi^{5}r^{4}w_{\text{out}-\text{bend}}^{2}}{L_{1}^{3}}+\frac{E_{m}\pi r^{4}\theta_{r}^{2}}{8L_{0}\left(1+\nu \right)} \end{array}$$ (8)

where θ_r_ is the revised torsion angle. Through the combination of arch and chiral structures, compression-bend-torsion multimode coordinated deformation is accomplished, which can prevent buckling. Besides, the additional out-of-plane bend deformation further modulate the *K*_eff_ of metacells, contributing to the buffering and piezoelectric output improvement. In this study, the arch metacells were labeled NA, the chiral metacells were labeled as CL, and the chiral arch metacells were labeled as CA. On the basis of these metacells, the helical metacells were labeled as NAH, CLH, and CAH, respectively.

To validate the theoretical analysis and elucidate the large-deformation behaviors, we further simulated the quasistatic response of various metacells via the finite element analysis (FEA) method. For default models without special declaration, *r* and *h*_0_ were set to 0.6 and 10 mm, respectively, whereas the metacell section was a triangle, where *R* of the inscribed circle was 5 mm. In addition, the original maximum deflection (*w*_in_) of the arch rod in NA and NAH metacells was 2 mm. The force-displacement curves were shown in Fig. 3B and fig. S9; it indicated that the introduced helical structure effectively prevent buckling and improve stress transfer, where *K*_eff_ of CAH was the minimum, about 5 kN/m. As a result of compression-bend-torsion deformation coordination, the mechanical response of CAH metacells was steady, exhibiting large recoverable deformation without buckling (movie S1). Furthermore, the macroscopic compression-torsion coordination effect of chiral metacells, including CL, CLH, CA, and CAH, were evaluated, and the torsion angle (γ)–displacement curves were shown in Fig. 3C. It was found that γ decreased moderately due to the competitive allocation of strain energy into the additional out-of-plane bend deformation, which was accurately captured by the analytical predictions. The primary objective of the proposed analytical model is to mathematically decouple and validate the localized multimode deformation coupling mechanism of metacells.

As shown in Fig. 3D, the FEA simulation output was consistent with the analytical model of CAH metacells within the small-deformation regime. Specifically, it was found that the strain energy was dominated by the compression when displacement was less than 1%, and the in-plane bend occurred due to the lower-order deflection when the deformation was larger than 1%. When the deformation reached higher than 2%, out-of-plane bend turned to be the domination. This high consistency in the early elastic stage firmly validates the designed multimode coupling deformation, providing an essential theoretical foundation for the synergistic excitation of multiple piezoelectric modes. As the deformation reached beyond 3%, deviation appeared due to the limitations of small-deflection Euler-Bernoulli beam theory under large nonlinear deformation. Specifically, large deformations trigger the breakdown of the constant curvature approximation, induce strong geometric coupling (e.g., P-Delta effects and complex bending-torsion interactions) (*54*). Therefore, nonlinear FEA and experimental evaluations are leveraged as the robust and standard tools to autonomously adapt to these large-deformation behaviors and accurately solve the macroscopic load-bearing and energy-absorption performances (*55*).

Furthermore, the influence of structure parameters on mechanical behaviors of various metacells was studied, and it exhibited that the increase in rod diameter is capable to improve the strength and stiffness, as shown in fig. S10. The stiffness of CAH improved from 0.17 kN/m to 8.3 kN/m when diameter increased from 0.6 mm to 1.4 mm (Fig. 3E). However, the increased diameter led to the sharp decline of reaction force beyond a certain displacement threshold, especially in chiral metacells, which is detrimental to the stability and reliability during energy absorption (fig. S11). The nephograms of stiffness, recoverable displacement, and bearing capacity indicated that the mechanical response of CAH metacells can be modulated through structure optimization, including cell height and rod oblique angle (Fig. 2, F and G, and fig. S12).

### Mechanical performances of Kagome lattice–inspired metamaterials

**Fig. 4.**
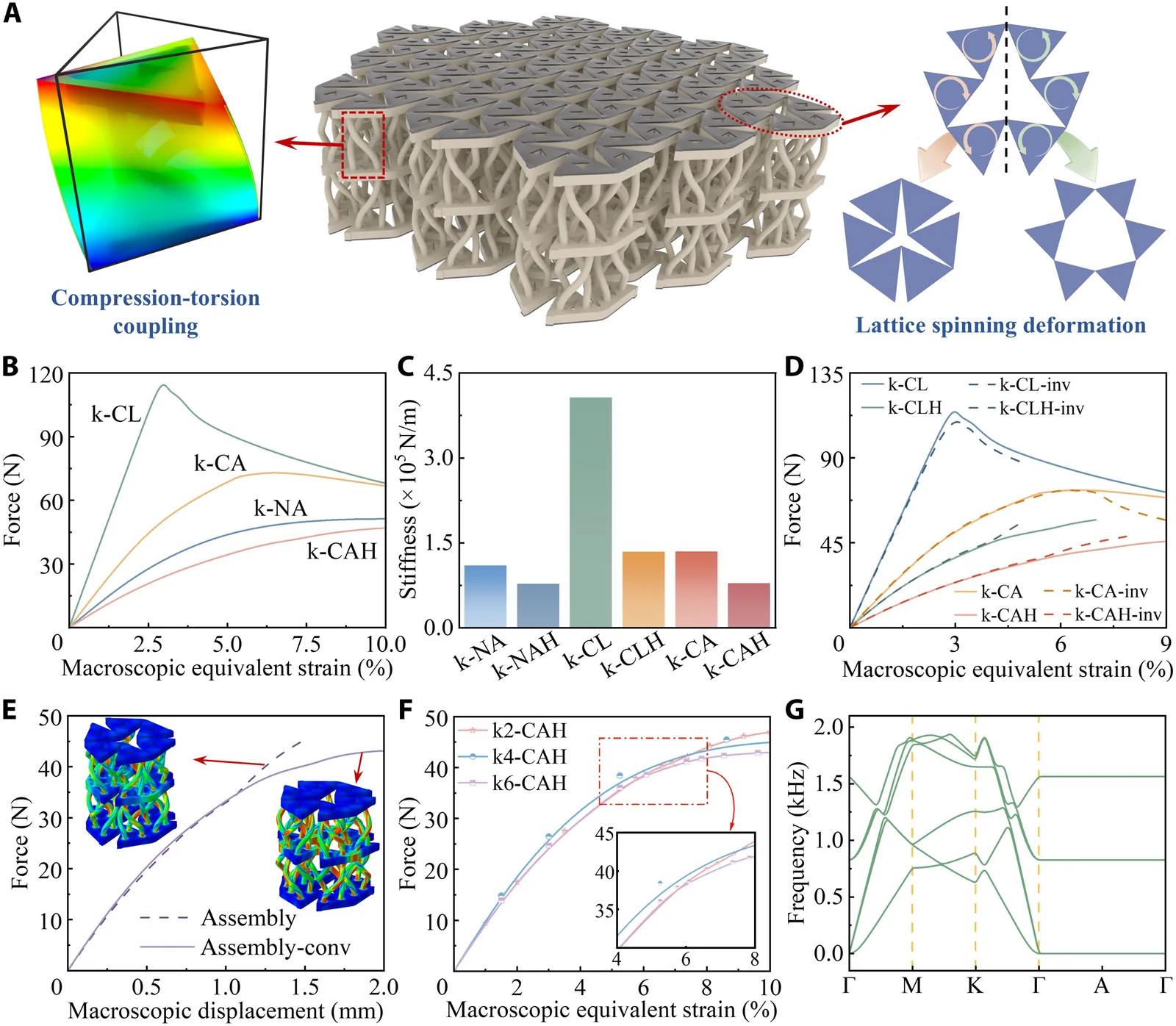
Mechanical simulation of Kagome lattice–inspired metamaterials. (**A**) Vertical-lateral coupling deformation mechanism of the assembled CAH metamaterial inspired by the Kagome lattice topological structure. (**B**) Force-strain curves of Kagome lattice–inspired NA (k-NA), CL (k-CL), CA (k-CA), and k-CAH. (**C**) Stiffness of k-NA, Kagome lattice–inspired NAH (k-NAH), k-CL, Kagome lattice–inspired CLH (k-CLH), k-CA, and k-CAH metamaterials. (**D**) Force-strain curves of k-CL and k-CL in inverse helical direction (k-CL-inv), k-CLH and k-CLH in inverse helical direction (k-CLH-inv), k-CA and k-CA in inverse helical direction (k-CA-inv), and k-CAH and k-CAH in inverse helical direction (k-CAH-inv). (**E**) Force-strain curves of the two-layer-assembled k-CAH (assembly) and assembly structure in inverse helical direction (assembly-inv). (**F**) Force-strain curves of k-CAH metamaterials with various *L*_1_/*L*_2_ of 0.2 (k2-CAH), 0.4 (k4-CAH), and 0.6 (k6-CAH). (**G**) Phonon spectra of the k2-CAH metamaterial.

On the basis of the above metacells, the piezoelectric metamaterials were constructed following the topology of Kagome lattice structure, made up of interlaced triangles and hexagons. The unique band structures and spin frustration of Kagome lattice have the potential to construct metamaterials for mechanical protection and energy recycling when extending classical waveguide theory to elastic waves (*56*, *57*). In addition, Kagome lattice exhibits excellent isotropic elasticity due to the *N*^1/2^ modes of low-energy deformation, which imparts the structure with the desired properties of lightness and buckling resistance and make it suitable for structural materials (*58*, *59*). The constructed metamaterial with CAH metacells were labeled as k-CAH, whereas metamaterials based on NA, NAH, CL, CLH, and CA metacells, as comparison groups, were labeled as k-NA, k-NAH, k-CL, k-CLH, and k-CA, respectively. Without special declaration, the short/long inner dimension ratio (*L*_2_/*L*_1_) was set to 0.2 (fig. S13). The metamaterial deformation can be resolved into vertical and lateral parts, where the vertical deformation consists of compression-bend-torsion coordination of chiral metacells and the lateral deformation is lattice-spinning-like deformation (Fig. 4A). Furthermore, the compression-torsion coordination along the vertical direction can lead to the spinning along the lateral direction, achieving integrated multimodal deformation. As the vertical torsion results in the lateral spinning direction, the influence of chirality on mechanical response of k-CAH metamaterials was studied via FEA. We set the spinning direction that led to the fold of Kagome lattice as common chirality while the inverse chirality led to the dilatation of Kagome lattice (Fig. 4A). The mechanical responses of constructed metamaterials were first evaluated via FEA, and it was found that the topology method inspired by Kagome lattice can notably improve the strength, compared to the simple parallel connection of metacells (Fig. 4B and fig. S14). The force of k-CAH metamaterials climbed steadily during compression, desirable for mechanical buffering, and the stiffness of k-NAH and k-CAH were the minimum, about 7.9 × 10^4^ N/m, only 17.6% of the k-CL (Fig. 4C).

According to the force-strain curves of chiral metamaterials in Fig. 4D, it was found that the mechanical responses of different chirality structures during small deformation were similar due to the low energy cost of Kagome topology. Moreover, buckling is more obvious in the inverse k-CL and k-CA, which indicated the advantages of auxetic in durability and conformality. As summarized in Table 2, the modal analysis results indicated that the first (652.92 Hz) and second (653.00 Hz) natural frequencies of k-CAH are nearly degenerate with a minimal gap of only 0.08 Hz. This exceptional modal proximity could trigger a strong modal coupling effect, laying the structural foundation for the broadband vibration isolation capabilities. We further assembled the k-CAH and inverse k-CAH metamaterials along the vertical direction to amplify the lateral deformation. As shown in Fig. 4E, the force-displacement curves exhibited that the auxetic metamaterial assembly was better for energy absorption when vertical displacement was larger than 1 mm (the assembly height is 20 mm). To optimize the topology strategy, we evaluate the compression and vibration response of the k-CAH metamaterial with the *L*_2_/*L*_1_ of 0.2 (k2-CAH), 0.4 (k4-CAH), and 0.6 (k6-CAH), respectively (figs. S15 and S16). The force-strain curves showed that the k2-CAH metamaterial had the largest recoverable energy absorption capacity, and the phonon spectra of k2-CAH exhibited a flat band along the path of Γ-A, which is desired for elastic wave absorption (Fig. 4, F and G). It was further found that the first natural frequency of k2-CAH, around 652.92 Hz, was the lowest compared to k4-CAH and k6-CAH (table S1). This proximity of natural frequencies triggers a strong modal coupling effect, which substantially broadens the mechanical attenuation bandwidth, thereby endowing the structure with exceptional vibration isolation capabilities (*60*). As *L*_2_/*L*_1_ rose, the first natural frequency of metamaterials significantly increased up to 670.94 Hz, demonstrating that the vibration protection spectrum of the k-CAH metamaterial can be geometrically tailored to target specific external excitations. Meanwhile, the vibration mode of k-CAH metamaterials was primarily axial in the vertical direction according to the mode shape in fig. S17. The maximum deformation occurred at the edge of Kagome lattice on the lateral surface, and there was no significant buckling even at high-order vibration modes.

**Table 2.** Natural frequency of metamaterials when the bottom was fixed and the top was free.

Order	Natural frequency (Hz)					
	k-NA	k-NAH	k-CL	k-CLH	k-CA	k-CAH
1	441.46	429.89	796.65	682.83	762.54	652.92
2	441.50	429.93	796.77	682.87	762.65	653.00
3	484.46	481.26	939.60	841.65	929.47	835.11
4	924.63	917.94	1120.50	922.69	1081.70	913.70
5	990.95	986.50	1247.00	1067.50	1228.40	1072.30
6	991.20	986.87	1247.20	1067.70	1228.60	1072.60

**Fig. 5.**
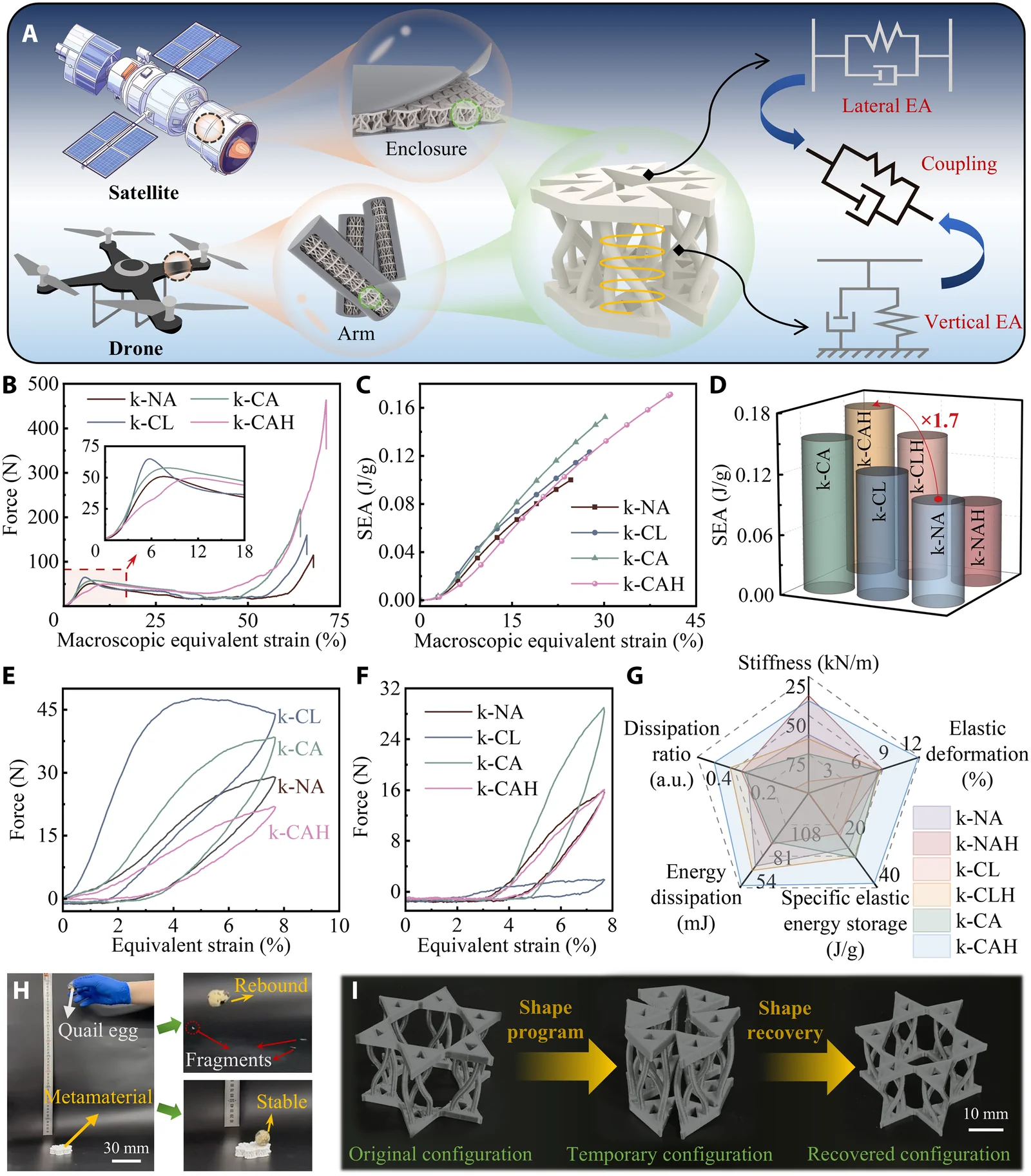
Energy absorption and durability of metamaterials. (**A**) Schematic diagram of k-CAH metamaterials for construction of key components in satellites and drones and mechanism of energy absorption. (**B**) Force-strain and (**C**) SEA-strain curves of various metamaterials. (**D**) Maximum SEA of various metamaterials. The force-strain curves of k-NA, k-CL, k-CA, and k-CAH metamaterials (**E**) at the initial stage and (**F**) the stage after 200 cycles. (**G**) Comprehensive comparison of the mechanical behaviors of various metamaterials. (**H**) Buffering performance and (**I**) shape memory cycle of k-CAH metamaterials.

The energy absorption capacity of designed metamaterial was derived from the coupling of lateral and vertical damping. The multimode-coordinated deformation of k-CAH metamaterials along the vertical direction was capable to absorb large elastic energy and induce the lateral spinning for dissipating energy via the shearing process (Fig. 5A). The Kagome-inspired topology could improve the coupling efficiency of vertical energy absorption units and thus increase the fraction of recoverable energy absorption. The coupled damping effect of k-CAH metamaterials made it promising for mechanical protection in aerospace structures as well as civil aircrafts, including satellites and drones. The quasistatic compression tests were performed to evaluate the energy absorption ability of metamaterials (Fig. 5B and fig. S18). It was found that k-CA combined the high energy absorption efficiency of k-CL and large recoverable deformation of k-NA, effectively preventing buckling. In addition, the helix structure optimization further decreased the stiffness and improved the recoverable deformation ratio and total energy absorption capacity, which was consistent with the results of FEA. During the compression process, the structure of k-CAH metamaterials maintained intact even under large deformation, resulting from the multimode coordinated deformation mechanism (movie S2). As shown in Fig. 5 (C and D) and fig. S19, k-CA exhibited the highest energy absorption efficiency and the total specific energy absorption (SEA) of k-CAH was the maximum, reaching 0.18 J/g, which was 1.7 times that of the k-NA. According to table S2, the 4D-printed k-CAH metamaterials exhibited superior SEA, compared to the common structures fabricated via additive manufacturing technologies.

The cyclical compression test was further carried out to evaluate the durability of metamaterials, as shown in Fig. 5 (E and F) and fig. S20. The force-strain curves at the initial stage and after 200 cycles indicated that k-CAH had a superior capacity to maintain mechanical response. The area of force-strain loops represents the dissipated energy during cyclical compression, which cannot be recovered after deformation and exerts a negative effect on the energy harvest. It was found that the irrecoverable energy of k-CAH was the minimum, about 55.2 mJ, which was only about a quarter of k-CL. By comparing the stiffness, elastic deformation, SEA, and dissipation ratio, k-CAH was desired for integrating energy absorption and harvesting (Fig. 5G). The mechanical buffering performance of k-CAH was assessed through a quail egg drop experiment, where the eggshell maintained intact when the quail egg was buffered by the k-CAH metamaterial and the egg dropped on the ground was smashed (Fig. 5H and movie S3). The 4D-printed metamaterial was capable to achieve structure reconfiguration due to the shape memory properties of the PLUG matrix. Through the dynamic mechanical analysis (DMA), we found that the glass transition temperature (*T*_g_) was 76.15°C (fig. S21). For shape memory assessment, the original k4-CAH was programmed into k2-CAH after being heated to 75°C and maintained, whereas the temporary configuration could rapidly recover to the initial when reheated to 75°C (Fig. 5I). The shape memory capacity made it possible to program the configuration and performances of 4D-printed structures in response to the specific conditions, which broadened the application of metamaterials.

### Piezoelectric response of piezoelectric metamaterials

**Fig. 6.**
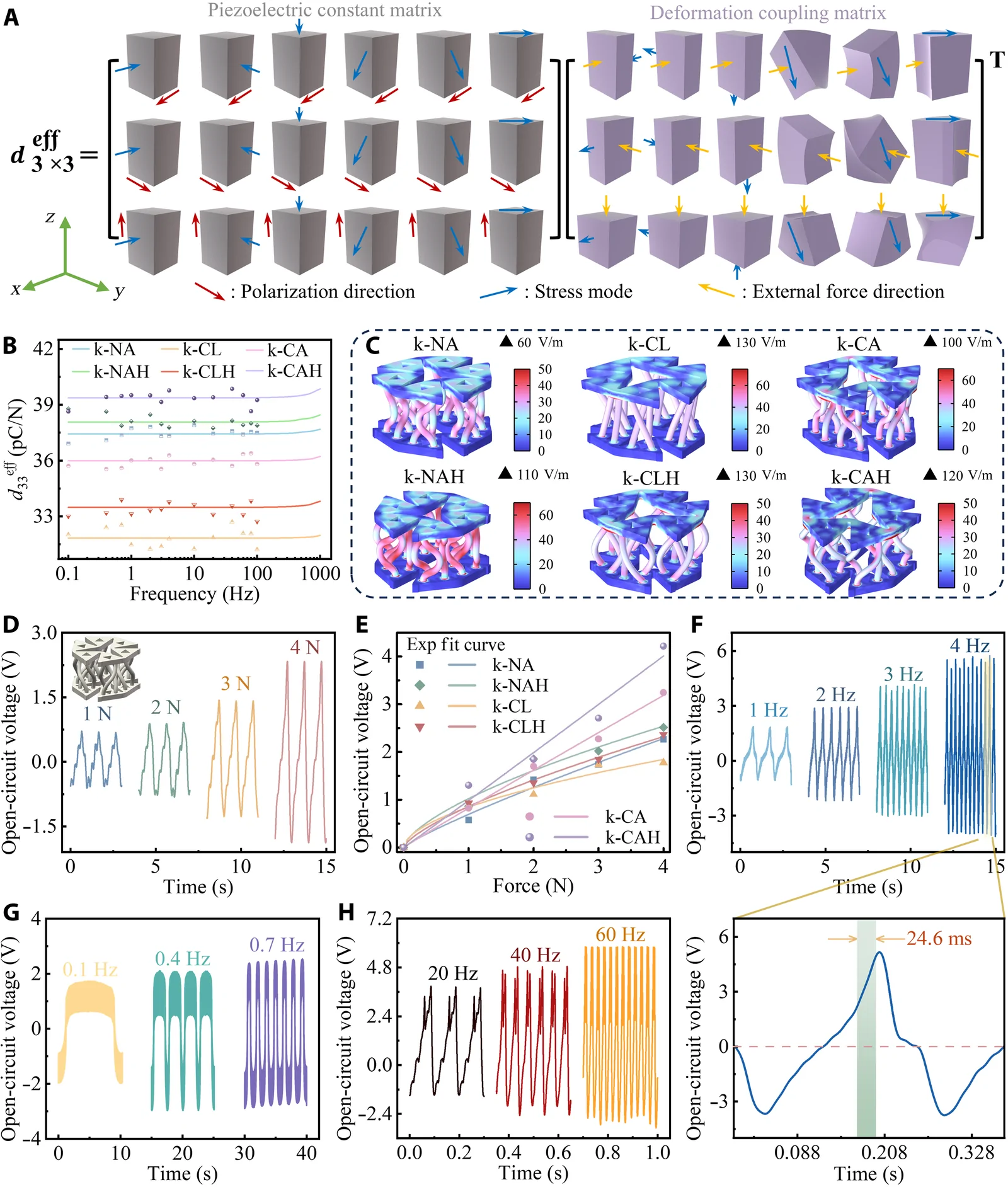
Output performances of piezoelectric metamaterials. (**A**) Schematic diagram of the effective piezoelectric matrix ($d_{3\times 3}^{\text{eff}}$), composed of naturally electromechanical conversion and coupled mechanical deformation parts. (**B**) Simulated (line) and measured (scatter) $d_{33}^{\text{eff}}$ of the designed architected metamaterial. (**C**) Simulated results of the electric field distribution of various metamaterials (2 N, 10 Hz). (**D**) Open-circuit voltage output of k-CAH metamaterials under different load conditions at the frequency of 1 Hz. (**E**) Voltage-force curves of various piezoelectric metamaterials (at 1 Hz). Open-circuit voltage of k-CAH metamaterials under a load of 3 N, at a frequency of (**F**) 1 to 4 Hz (lower), (**G**) 0.1 to 1 Hz (quasistatic), and (**H**) 20 to 60 Hz (higher).

Previously, researchers mainly attempted to control piezoelectric response mode through electrode placement and polarity orientation, which was complex and high-cost for 3D-architected piezoelectric devices. The material-architecture-manufacture integrated strategy for piezoelectric metamaterials in this study offers a new approach for promoting energy harvesting efficiency and state monitoring accuracy. Apart from the energy absorption and buffering improvement, the multimode coordination of metacells and multidirection coupling of topology strategy could effectively activate various natural piezoelectric modes, such as *d*_33_, *d*_31_, and *d*_15_. Through the combination of piezoelectric coefficient matrix of materials and deformation coupling matrix, we constructed a matrix with higher effective piezoelectric coefficients ($d_{3\times 3}^{\text{eff}}$), which illustrated the mechanism of output improvement (Fig. 6A). To evaluate the output capacity of 4D-printed piezoelectric metamaterials, an experimental platform was built with an electrometer, a signal generator, an amplifier, an excitor, a pressure sensor, and a data acquisitor (fig. S22). Considering that the vibration environment of aerospace equipment in space mainly ranged from 0.1 to 60 Hz (*61*, *62*), we focused on the piezoelectric response under microvibration conditions. As shown in Fig. 6B, the $d_{33}^{\text{eff}}$ of k-CAH was the maximum (39.8 pC/N), exceeding other architectures, which proved that the helix-chiral-arch topology acted as a high-efficiency amplifier for electromechanical transduction. Meanwhile, the $d_{33}^{\text{eff}}$ of architected metamaterials remain remarkably stable across the range of 0.1 to 1000 Hz, guaranteeing a highly reliable and stable piezoelectric response under low-frequency vibration conditions for potential aerospace applications. To evaluate the performances of various piezoelectric metamaterials, we simulated the piezoelectric output, under 2 N at 10 Hz (Fig. 6C). The electric field distribution nephogram showed that the combination of arch and chiral structure in k-CA substantially improved the response strength and prevented concentrated response, and the helix structure could effectively induce the piezoelectric effect. As the generated piezoelectric power reaches maximum at the resonance frequency (*63*), we carried out further simulation for frequency response of piezoelectric metamaterials. The induced potential of piezoelectric metamaterials was significantly higher than the ordinary bulk at the same shape (fig. S23). In addition, the open-circuit voltage of k-CAH reached the peak at around 800 Hz, which was consistent with the results of phonon spectra, resulting from the multiple resonance of the structure. The relationship of structure (*L*_2_/*L*_1_) and chirality to the piezoelectric voltage output was further studied, and the nephogram indicated that the maximum value occurred at the *L*_2_/*L*_1_ of 0.5 when the frequency was lower than 980 Hz (fig. S24). The measured open-circuit voltage climbed with the increasing load, and k-CAH exhibited the highest output at the same load, reaching 2.45 V under the load of 4 N (at 1 Hz; Fig. 6D and fig. S25). The voltage-load curves in Fig. 6E further proved the relationship, where the output voltage of k-CA and k-CAH varied linearly with the applied load. The recorded signal showed that the output of piezoelectric metamaterials increased to some extend with increasing load frequency, consistent with the simulation results, and the response was accurate, reaching up to 24.6 ms at the load frequency of 4 Hz (Fig. 6, F to H, and fig. S26). It was also found that output spectra at lower frequency (0.1 to 4 Hz) exhibited single peak whereas those at higher frequency (20 to 60 Hz) had double-peak fluctuation, resulting from the self-resonance of the structure under the high frequency load. Moreover, the k-CAH piezoelectric metamaterial maintained a desirable piezoelectric response over the microvibration range, significantly exceeding other architectures.

**Fig. 7.**
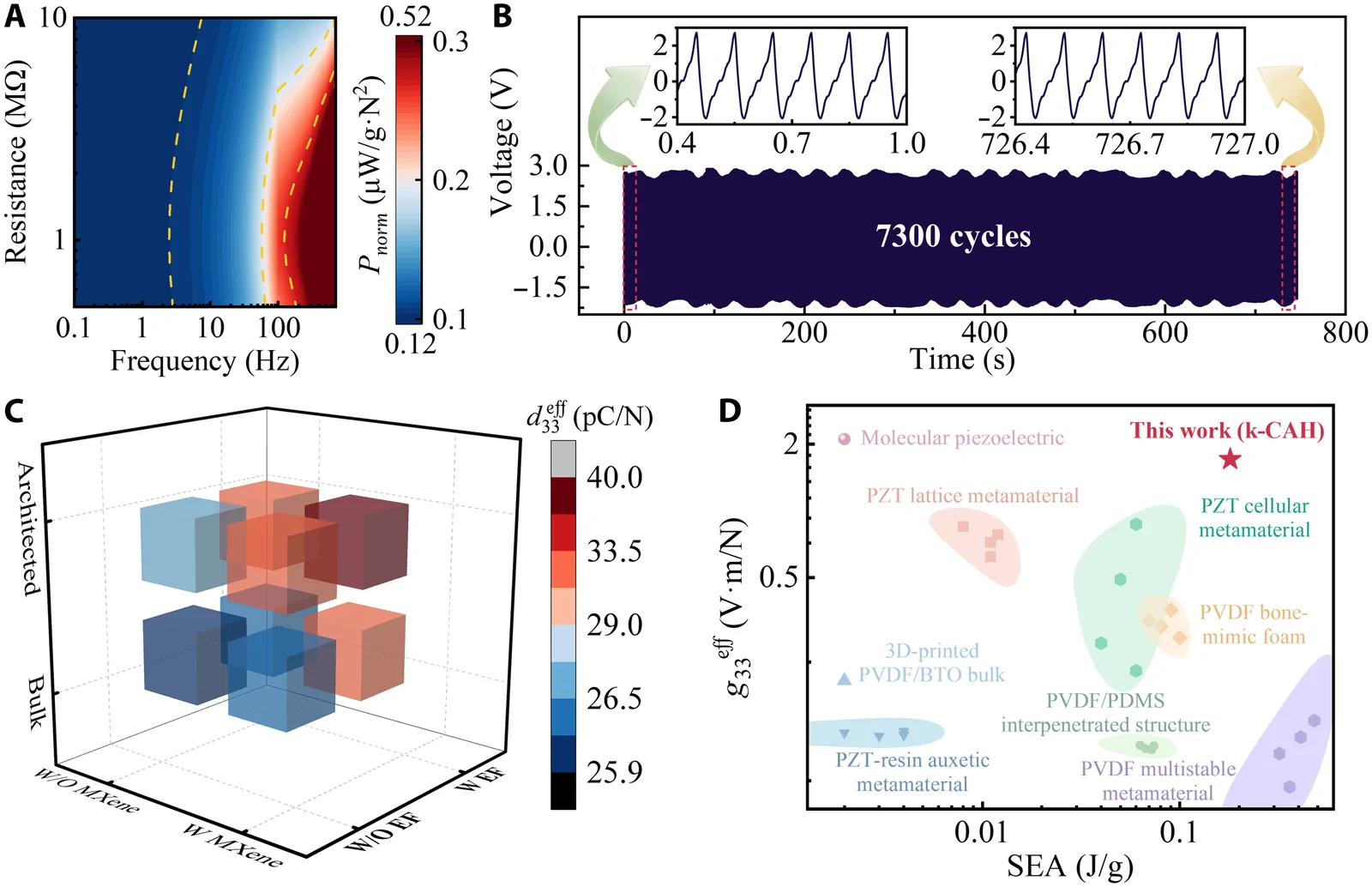
Piezoelectric performances of the metamaterial following the material-architecture-manufacture integrated strategy. (**A**) Power density-frequency nephogram of k-CAH metamaterials with various external load resistances. (**B**) Piezoelectric output durability of k-CAH metamaterials after 7300 cycles (3 N, 10 Hz). (**C**) Mapping of the $d_{33}^{\text{eff}}$ magnitude across a 2×2×2 parameter space, where three axes represent the independent design domains: material component [*x* axis: without (W/O) or with (W) MXene), multifield coupling manufacturing process [*y* axis: W/O or W in situ poling electric field (EF)], and architecture topology (*z* axis: bulk or architected metamaterial). (**D**) Comparison of the piezoelectric metamaterial, following the material-architecture-manufacture integrated strategy, with other piezoelectric architectures on mechanical and piezoelectric performances (*36*, *65*–*72*). PDMS, polydimethylsiloxane.

For output capacity evaluation, we found that the maximum normalized power density per cycle (*P*_norm_), around 0.52 μW/g·N^2^, occurred when the load resistance reached the amplitude of 3 MΩ, and the output capacity reached peak at around resonant frequency (Fig. 7A). Notably, the *P*_norm_ at far-off-resonant frequency maintained within the same order of magnitude as the resonant response, resulting from the geometry-induced local strain amplification mechanism (fig. S27). The durability of k-CAH metamaterials was evaluated, and the response curves after 7300 cycles were the same as the initial curves (Fig. 7B). To decouple the effect of material component, manufacture process and architecture design on the piezoelectric performance, we systematically compared the $d_{33}^{\text{eff}}$ with eight groups and mapped them in a 3D space (material, manufacture, and architecture) (Fig. 7C). Although independent implementation of each dimension yields modest increase, the fully integrated group (with MXene, with EF, Architected) exhibits a remarkable improvement in $d_{33}^{\text{eff}}$ compared to the control group (without MXene, without EF, Bull), from 25.9 to 39.8 pC/N. This explicitly validates the synergistic material-architecture-manufacture coupling mechanism, where geometry-induced local strain amplification synergizes with the heterogeneous intercalation network to maximize electromechanical transduction. To calculate the $g_{33}^{\text{eff}}$ following Eq. 1, the equivalent ε (ε_eq_) was expressed as (*64*)

$$\varepsilon_{\text{eq}}=\varepsilon_{\text{mat}}V+\varepsilon_{0}\left(1-V\right)$$ (9)

where ε_mat_, ε_0_, and *V* represents the material dielectric constant (at 100 Hz), air dielectric constant, and volume fraction of the metamaterial, respectively. Notably, the piezoelectric metamaterial (k-CAH) fabricated with the material-architecture-manufacture integrated strategy exhibits a significant enhancement in both piezoelectric ($g_{33}^{\text{eff}}$ of 1.712 V·m/N) and mechanical (SEA of 0.18 J/g) performances, outperforming the previously reported piezoelectric composites and architectures (Fig. 7D) (*36*, *65*–*72*).

### Real-time sensing and energy harvesting evaluation

**Fig. 8.**
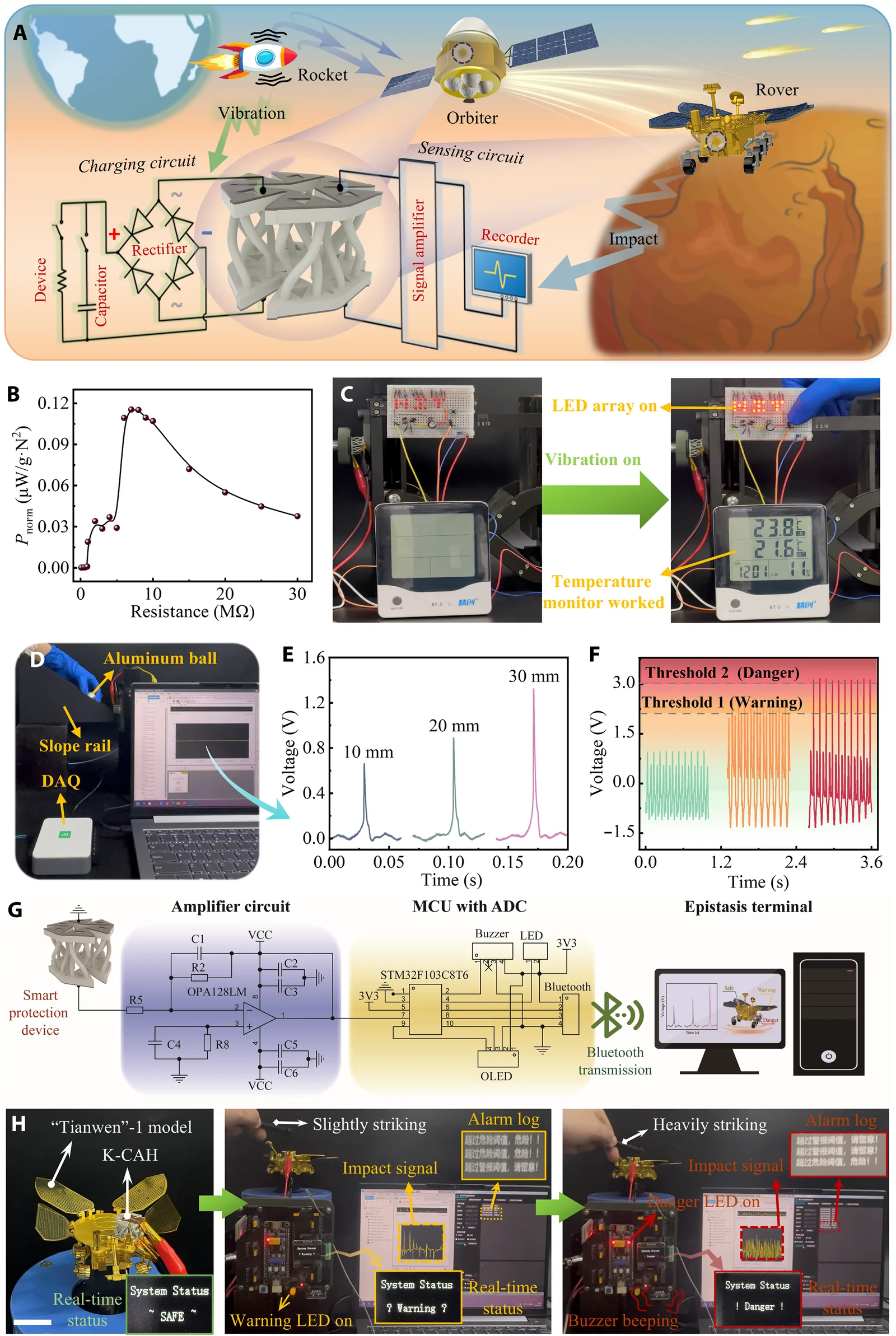
Self-powered sensing and energy harvesting for the “Tianwen”-1 Mars rover embedded with the 4D-printed piezoelectric k-CAH protector. (**A**) Schematic diagram of the application of the 4D-printed piezoelectric protector for vibration energy recycling during launch and real-time impact monitor at space. (**B**) Resistance matching curve of the k-CAH piezoelectric metamaterial, under a load of 2 N at 10 Hz. (**C**) Energy harvesting performance of the k-CAH metamaterial, simultaneously lighting up an LED array, powering an ink screen temperature monitor and charging a 100-μF capacitor. (**D**) Experimental platform for the self-powered sensing performance of the 4D-printed piezoelectric metamaterial. (**E**) Sensitivity of the k-CAH protection system for the impact load of an aluminum ball dropped from different heights. (**F**) Voltage signal thresholds (V1 and V2) and corresponding alarming states (LED/buzzer), controlled by a customized MCU. (**G**) Schematic of the smart protection system, consisting of the k-CAH piezoelectric metamaterial, amplification circuit, MCU with ADC, alarming devices, and signal transmission modules. (**H**) Activation states of the “Tianwen”-1 Mars rover model equipped with the k-CAH protector (under safe, warning, and dangerous conditions). Scale bar, 2 cm.

As the 4D-printed k-CAH piezoelectric metamaterials exhibited superior mechanoelectrical conversion performance, it was promising for energy harvesting and self-powered sensing with the assistance of external circuit. Specifically, the modern spacecrafts [e.g., satellites, orbiters, and rovers], embedded with piezoelectric k-CAH, can recycle the vibration energy during the launch process as well as in space environment and monitor the real-time impact without external supply (Fig. 8A). It was found that the resistance of k-CAH metamaterials was around 7 MΩ, and the highest *P*_norm_ was up to 0.12 μW/g·N^2^ (Fig. 8B), consistent with the simulation results. For energy recycling evaluation, we connected an array of 19 light-emitting diodes (LEDs), a 100-μF capacitor, and an ink screen temperature monitor, as external circuit loads (connected in-parallel), to the ends of k-CAH (Fig. 8C). Under a load of 2 N (at 10 Hz), the LED array was lighted and the temperature monitor was powered, and the capacitor was charged at a rate of 86 mV/s (fig. S28 and movie S4). As comprehensively compared in table S3, conventional piezoelectric devices inevitably suffer from intrinsic functional trade-offs. Resonant piezoelectric harvesters exhibit high efficiency at the severe cost of bandwidth, whereas flexible piezoelectric sensors have high sensitivity but critically low energy conversion efficiency. The k-CAH piezoelectric metamaterial concurrently delivers a broad operational bandwidth (0.1 to 60 Hz), a robust energy conversion efficiency (1.35%) under far-off-resonant conditions (10 Hz), and high perceptual sensitivity (224.3 mV/kPa), thereby integrating efficient microvibration energy harvesting with high-precision self-powered state sensing. Furthermore, conventional piezoelectric transducers suffer from severe efficiency decrease at low frequencies, far below their resonance, as they rely heavily on dynamic inertial forces to accumulate effective strain. Meanwhile, our architecture acts as a mechanical strain amplifier due to the unique multideformation mode coupling mechanism. Consequently, the piezoelectric metamaterial overcomes the low-frequency barrier by relying on geometry-driven kinematic amplification rather than frequency-dependent resonance, thereby unlocking unprecedented electromechanical transduction efficiency under microvibration stimuli. This indicated that the protection system had potential to recycle the vibration energy in space and drive the low-power components for long-time monitoring and information transmission.

We further carried out a ball-dropping test to evaluate the sensing accuracy of the k-CAH piezoelectric metamaterial, and it was found that the response potential signal was improved with the increasing dropping height (Fig. 8, D and E). It was also found that the k-CAH piezoelectric metamaterial had superior sensing accuracy, which could sense the load applied by hammer under varying amplitude and frequency (movie S5). A smart protection system was constructed with k-CAH as well as a matched signal amplifier circuit and alarming devices, including buzzer, LED, and OLED screen. The load signal was transmitted to the epistasis terminal through Bluetooth module, which could be further analyzed to detect the conditions of equipment. Moreover, we set two voltage thresholds for reminding the real-time impact load, representing warning and danger status, respectively (Fig. 8, F and G, and fig. S29). To validate the self-powered real-time monitoring capacity, the smart protection system was embedded on a “Tianwen”-1 Mars rover model (1:30) and the model was fixed on a vibration excitor to simulate the working condition (Fig. 8H). During the vibration process, we stroke the metamaterial with a hammer. When the voltage peak was higher than 2.2 V, the yellow LED twinkled and alarming information of “? Warning ?” was presented on the OLED screen (movie S6). Furthermore, the buzzer beeped and the red LED twinkled when the voltage peak reached higher than 3.0 V, and alarming information of “! Danger !” appeared on the OLED screen at the same time. Moreover, a warning log was transmitted to the epistasis terminal for alarming the corresponding status. It was proved that the smart protection system worked as design, which showed a great potential for the next-generation structural material integrating mechanical buffering, energy recycling, and real-time impact sensing. Looking ahead, the inherent dual-functionality of the 4D-printed piezoelectric metamaterials paves the way for closed-loop systems, where real-time sensing signals can trigger shape reconfiguration to achieve autonomous environmental adaptation and controllable energy recovery. To practically realize and scale these systems, it is desired to explore hybrid multiprocess additive manufacturing for integration of conformal circuits and monolithic MCU with structural architecture, thereby achieving fully integrated electronic systems.

## DISCUSSION

In summary, we have presented a novel material-architecture-manufacture integrated strategy for the smart piezoelectric metamaterial protector, simultaneously achieving large recoverable deformation, high energy absorption, and self-powered real-time sensing. This study establishes a mechanism-driven decoupled optimization paradigm synergizing material, manufacture, and architecture. At the material level, through tailoring the ratio of MXene nanosheets to piezoelectric K-BTO nanoparticles, the constructed heterogeneous intercalation network in the shape memory PLUG-BM composite could not only mitigate agglomeration but also effectively induce interfacial polarization and reduce dielectric loss. At the manufacture level, the synergy of electric and fluidic fields achieved in situ poling of piezoelectric K-BTO and orientation of MXene nanosheets, as distributed microelectrodes, during the electric field–assisted 4D printing. This in situ mechanism effectively overcomes the severe dielectric breakdown during conventional postprinting corona poling treatment. Integrating the material modulation and multifield synergistic manufacturing, *d*_33_ and *g*_33_ reached up to 41.5 pC/N and 0.763 V·m/N, respectively, which were significantly higher than most commercial piezoelectric polymers (*73*–*75*). At the architecture level, the compression-bend-torsion coordination and multidirection coupling mechanism of k-CAH metamaterials balanced the conflict between recoverable deformation and energy absorption, accomplishing high SEA (0.18 J/g). The designed topological metamaterials translate a single macroscopic uniaxial load into localized multiaxial stress fields, synergistically activating multiple intrinsic piezoelectric modes (intrinsically coupling the longitudinal *d*_33_, transverse *d*_31_, and shear *d*_15_ modes). Integrating 4D printing technology and structure design, configurations and functions of architected metamaterials could be reprogrammed to meet the requirement of complex tasks. Crucially, the synergy of the heterostructure construction, in situ poling process, and prebuckling geometry design leads to a marked improvement on device-level piezoelectric capacities (39.8 pC/N in $d_{33}^{\text{eff}}$ and 1.712 V·m/N in $g_{33}^{\text{eff}}$). The k-CAH–based system exhibited superior energy conversion efficiency, operational bandwidth, and sensitivity under microvibration conditions, compared to conventional piezoelectric transducers and self-powered flexible sensors.

Ultimately, by integrating the 4D-printed k-CAH with an MCU, energy harvesting, and alarming devices, the intelligent protection system could absorb impact energy while simultaneously monitoring real-time impact, with no need of external power sources. These advances could expand the design space for next-generation structural materials in autonomous systems in aerospace and robotics. Although a mechanism-verified local optimum was achieved in the current work, validating our integration strategy, it remains an exciting frontier to achieve a global synergistic optimum. For this estimate goal, deep learning algorithms and AI-driven generative design will become feasible approaches. Such advanced computational models will exponentially expand the optimization landscape from current discrete empirical frame to continuous, hyperdimensional spaces, unlocking the design limits of such multifunctional metamaterials.

## MATERIALS AND METHODS

### Materials

t-BTO (*D* = 0.6 to 1 μm) was purchased from Macklin Biochemical Technology Co. Ltd. (Shanghai, China). TMSPMA (≥97%) was purchased from Aladdin Scientific Corp. (Shanghai, China). PLA polyol–based photocurable resin was obtained from eSUN Co. Ltd. (Shenzhen, China). PUA and PEG(200)DA was obtained from Yinchang New Materials Co. Ltd. (Shanghai, China). The conductive silver paste (Elec-W50) was purchased from ELEC Co. Ltd. (Shenzhen, China).

### Specimen preparation

First, K-BTO was prepared by a hydrothermal method. Eight grams of t-BTO powder was dispersed in 100 ml of deionized water, and TMSPA was added with a weight fraction of 6%. The solution was magnetically stirred at 60°C overnight, and the K-BTO powder was obtained by the process of filtration and vacuum drying at room temperature for 48 hours. The KT-BTO powder was ground and further sieved into the size of 0.1 mm before being introduced into the shape memory resin. The shape memory resin was composed of commercial PLA polyol–based photocurable resin, PUA, and PEG(200)DA with a ratio of 6:4:0.1, which acted as a hard matrix, soft monomer, and cross-linker, respectively. The composite slurry was composed of KT-BTO, MXene, and shape memory resin, with a ratio of 30:1.5:100. Subsequently, the intelligent metamaterials were fabricated via a DLP printer (DF2+, Raise3D Technology Co. Ltd., China). The exposure time was set at 4 s, and an electric field of 1.2 MV/m was applied to induce the in situ poling of piezoelectric K-BTO during the 4D printing process. The specimens were modeled and transferred into STL format with the SOLIDWORKS CAD software (Dassault Systèmes, France) and further sliced with the IDEALMAKER software (Raise3D Technology Co. Ltd., China).

### Characterizations

The crystalline structure of the t-BTO and K-BTO was evaluated via an x-ray diffractometer (Rigaku, Japan), scanning from 5° to 80° at a rate of 5°/min. The function group structures were characterized by an x-ray photoelectron spectrometer (Thermo Fisher Scientific, USA). The molecular composition of the slurry was analyzed via a Fourier transform infrared spectrometer (Thermo Fisher Scientific, USA) in attenuated total reflection (ATR) mode, ranging from 400 to 4000 cm^−1^. The dielectric constants were measured with an impedance analyzer (PNA-X, Agilent, USA). The low-frequency dielectric constants, ranging from 0.1 to 1000 Hz, were measured at room temperature, using a precise impedance analyzer (Novocontrol Concept 80, Novocontrol GmbH, Germany). The *d*_33_ piezoelectric constants were measured at 100 Hz via a quasistatic piezoelectric analyzer (Huace, China). The *P*-*E* loops were measured by a ferroelectric test system (TF Analyzer 3000, aixACCT Systems, Germany) with a frequency of 10 Hz and a testing electric field of 30 kV/cm. The morphologies of the k-BTO nanoparticles and PLUG-BM composites were observed via a scanning electron microscope (AMBER, Tescan, Czech) with an accelerate voltage of 10 kV. The specimens were precoated with a thin layer of platinum. Piezoelectric characterizations were conducted with a piezoresponse force microscope (Bruker Dimension Icon, Bruker, Germany), where the SS-PFM loops were measured under the trigger voltage of 10 V.

### FEA section

The compression process and natural modes of vibration were simulated using the ABAQUS/standard software (Dassault Systèmes, France), considering nonlinear geometric deformation. Two rigid planes were set at both sides of the cell, and the bottom side was tied with the cell while the top side was connected to the cell with frictionless interaction. The bottom side was fixed while a displacement boundary condition was applied on the top side. The element type was set to C3D8R. The phonon spectra of different Kagome lattices were simulated with the COMSOL Multiphysics software (COMSOL, Sweden). The borders of the lattice were set to Floquet periodic boundary condition, and the scanning route was following “Γ-Μ-Κ-Γ-A-Γ.”

The response of piezoelectric metamaterials was simulated with the COMSOL software in frequency domain. The bottom side of metamaterials was fixed, and a cyclic load of 2 N was applied on the top side. The model was meshed with physics-controlled meshing, and a terminal was set at the top side of metamaterials. For the evaluation of $d_{33}^{\text{eff}}$, the generated potential was calculated through the surface integration of the top terminal, whereas an external parallel circuit (containing a capacitor and a load resistance) was connected with metamaterials and the voltage across the resistance was obtained for power calculation.

### Experimental section

The unidirectional compression and cyclical compression tests were carried out using a Zwick-010 tensile testing machine (ZwickRoell, Germany) at room temperature, where the load speed was set to 2 mm/min. Storage modulus and loss factor of SMPU were measured with a TA Q800 dynamic thermomechanical analyzer (TA Instruments, USA). For the evaluation of shape reconfiguration performance, shape memory cyclical tests were carried out with different deformations.

A self-built platform was used to assess the piezoelectric output of piezoelectric metamaterials under different load conditions. The platform was constructed with a signal generator (Changsha Rongqi Equipment Co. Ltd., China), an amplifier and a vibration exciter (Yangzhou Xinli Sensor Technology Co. Ltd., China), a programmable electrometer (6514, Keithley), and a data acquisitor. The metamaterials were fixed on the ground, and both sides were covered by a layer of silver slurry as an electrode, whereas the load amplitude and frequency were controlled with the input signal. For calculating the electromechanical transduction capacity of architected metamaterials, the electrometer was set to charge measurement mode. The $d_{33}^{\text{eff}}$ of the architected metamaterials was extracted from the slope (Δ*Q*/Δ*F*) of the charge-force curve. Furthermore, the piezoelectric metamaterials were connected with a rectifying circuit for powering LEDs and charging a 100-μF capacitor, which was used to power an e-paper temperature monitor. The input mechanical work per cycle was rigorously determined by integrating the dynamic force-displacement hysteresis loop, whereas the output electrical energy per cycle was determined via the rectified dc capacitor charging increment under a specific load. For self-powered sensing evaluation, the metamaterial was fixed on the ground and impacted with an aluminum ball (30 g) rolling along a slope from different heights (10, 20, and 30 mm). To confirm the potential of piezoelectric metamaterials for real-time sensing in spacecrafts, a “Tianwen”-1 Mars rover model (1:30) was equipped with the developed protection system, which was attached on the vibration table to simulate the condition of launch and space working processes. For the protection system, the metamaterial was connected to an MCU board (STM32F103C8T6, Yehuo) with a Bluetooth module (HC-05), analog-to-digital converter (ADC), and alarm modules (LED, OLED screen, and buzzer), whereas a laptop was used as a status receiver.

## Supplementary Materials

### The PDF file includes:

Supplementary Text Figs. S1 to S32 Tables S1 to S3 Legends for movies S1 to S6 References
