# Supplementary material for: Material-architecture-manufacture integrated strategy for piezoelectric metamaterials with mechanical protection and self-powered sensing
Source: Sci Adv. 2026 Sep 25;12(39):eaeg1744. doi: 10.1126/sciadv.aeg1744 (PMC13614385; doi:10.1126/sciadv.aeg1744)
Supplement: Supplementary file 1 [file sciadv.aeg1744_sm.pdf]

Science Advances
AAAS

Supplementary Materials for

Material-architecture-manufacture integrated strategy for piezoelectric metamaterials with mechanical protection and self-powered sensing

Zhicheng Wang *et al.*

Corresponding author: Xiaozhou Xin, xiaozhou_xin@163.com; Liwu Liu, liuliwu_006@163.com; Yanju Liu, yj_liu@hit.edu.cn

Sci. Adv. **12**, eaeg1744 (2026)
DOI: 10.1126/sciadv.aeg1744

The PDF file includes:

Supplementary Text
Figs. S1 to S32
Tables S1 to S3
Legends for movies S1 to S6
References

Other Supplementary Material for this manuscript includes the following:

Movies S1 to S6

Supplementary Text

S1. Analytical model of arch rod-based metacells

The strain energy of arch rod is attributed to the compression (U_{cpr}) and *in-plane* bend (U_{bend}) deformation. In this study, the constituent rods are analytically modeled utilizing a doubly-clamped boundary condition. It is important to distinguish between the global kinematics of the metacell and the local boundary conditions of the rods. Under the inherent compression-torsion coupling, the top triangular cap indeed undergoes a macroscopic rigid-body rotation and translation. However, as the structural nodes are fabricated as monolithic, rigid joints, there is no relative rotation between the rod ends and the connecting cap. Consequently, within the local co-rotational coordinate system of each rod, we mainly consider the doubly-clamped boundary, where the bottom end of rod is fixed while the top end of the rod can slide along the vertical direction but cannot rotate, as shown in **Fig. S30**. This model effectively decouples macroscopic nodal rotation from local clamped boundaries. Therefore, the effective stiffness (K_{eff}) of NA metacell is

$$K_{eff} = \frac{E_m \pi r^2}{L_0} = \frac{F_{rod}}{\varepsilon}, \qquad F_{rod} = \frac{F_v}{3} \tag{S1}$$

where L_0 is the original length of rods. Based on the beam theory, the compressive bending meets the **Eq. S2**:

$$E_m I \frac{d^4 w}{dy^4} + F_{critical} \frac{d^2 w}{dy^2} = 0, \qquad I = \frac{\pi r^4}{4} \tag{S2}$$

where $F_{critical}$ denotes the critical compression force for inducing bend deformation. Under the axial compression displacement ε, the solution to **Eq. S2** with the doubly-clamped boundary condition is

$$\begin{aligned} w(y) &= C_1 sinby + C_2 cosby + C_3 y + C_4 \\ w'(y) &= bC_1 cosay - bC_2 sinby + C_3 \\ w''(y) &= -b^2 C_1 sinay - b^2 C_2 cosay \end{aligned} \tag{S3}$$

where y is the position along the height direction. Considering the boundary conditions of bend deformation, we obtain

$$\begin{aligned} C_2 + C_4 = 0, \qquad C_1 b + C_3 = 0 \\ C_1 sinb\frac{L_1}{2} + C_2 cosb\frac{L_1}{2} + C_3\frac{L_1}{2} + C_4 = w_{bend} \\ C_1 cosb\frac{L_1}{2} - C_2 sinb\frac{L_1}{2} + C_3 = 0 \end{aligned} \tag{S4}$$

where w_{bend} and L_1 are the maximum deflection and deformed length of arch rods, respectively. For eigenvalue problem, *a=sqrt(*$F_{critical}$*/EI)*. Considering the 1st-order deflection shape of rod, we can specify:

$$
\begin{gathered}
n = 1, \qquad b = \frac{2n\pi}{L_1} = \frac{2\pi}{L_1}, \\
C_2 + C_4 = 0, \qquad C_1 b + C_3 = 0 \\
-C_2 + C_3 \frac{L_1}{2} + C_4 = w_{bend} \\
-C_1 \frac{L_1}{2} + C_3 = 0
\end{gathered} \tag{S5}
$$

The solution to **Eq. S5** is (*76*)

$$
\begin{gathered}
C_1 = C_3 = 0, \qquad C_2 = -C_4 = \frac{w_{in-bend}}{2} \\
w(t) = w_{bend}(1 - cosby)
\end{gathered} \tag{S6}
$$

where w_{bend}, L_1 and ε are the maximum deflection, deformed length of arch rods and vertical displacement, respectively. By analyzing the rod curvature, L_0 and L_1 can be solved by **Eq. S7**:

$$
\begin{gathered}
L_0 = \frac{2\alpha}{\pi cos\alpha_0} * \sqrt{h_0^2 + w_{bend}^2}, \\
L_1 = \frac{2\alpha}{\pi cos\alpha} * \sqrt{h_1^2 + w_{bend}^2}, \\
h_1 = h_0 - \varepsilon, \qquad \alpha_0 = arctan\frac{h_0}{2w_{bend}}, \qquad \alpha = arctan\frac{h_1}{2w_{bend}}
\end{gathered} \tag{S7}
$$

Thus, the critical load and compression displacement (s_{cri}) for internal bend deformation of rod is

$$
\begin{gathered}
F_{critical} = E_m I \left(\frac{2\pi}{L_0}\right)^2 = E_m I \frac{4\pi^2}{L_0^2}, \\
s_{cri} = L_0 - L_1
\end{gathered} \tag{S8}
$$

the stress and strain energy induced by compression deformation is

$$
\begin{gathered}
\sigma_{cpr} = \frac{E_m s}{L_0}, \\
U_{cpr} = \frac{E_m \pi r^2 s^2}{2L_0}
\end{gathered} \tag{S9}
$$

when bend deformation occurs, the strain energy is mainly determined by U_{bend} and the maximum stress (σ_{bend}) occurs at two ends and middle of the arch rod, which can be solved by **Eq. S10**:

$$U_{bend} = \frac{1}{2}\int_0^{L_1} E_m I w''^2(y)dy = \frac{E_m I w_{bend}^2 b^4}{4}\int_0^{L_1} 1 + cos2btdt = \frac{E_m \pi^5 r^4 w_{bend}^2}{L_1^3},$$
$$\sigma_{bend} = \frac{E_m \pi^2 r w_{bend}^2}{L_1^2} \quad (S10)$$

S2. Analytical model of chiral metacells

The chiral structure of CL metacell is capable to transfer vertical load into lateral torsion for boosting energy absorption efficiency, as shown in **Fig. S31(A)**. With the increasing vertical axial displacement (ε), additional *in-plane* and *out-of-plane* bend of the rod occurs due to the first order buckling (**Figs. S31, B** and **C**), and the critical buckling load for first order follows **Eq. S11**:

$$F_{critical} = \frac{4E_m \pi^3 r^4}{L_0^2} \quad (S11)$$

where L_0 is the original length of AB. In addition, L_1 is the length of AB'. The length and compression deformation (s) can be considered as **Eq. S12**:

$$L_0 = \sqrt{h_0^2 + 3R^2},$$
$$L_1 = \sqrt{(h_0 - \varepsilon)^2 + L_0^2 sin^2\beta}, \qquad \beta = arctan\frac{\sqrt{3}R}{h_0}, \quad (S12)$$
$$s = L_0 - L_1$$

According to **Eq. S9**, we can get

$$\sigma_{cpr} = \frac{E_m s}{L_0},$$
$$U_{cpr} = \frac{E_m \pi r^2 s^2}{2L_0} \quad (S13)$$

The *in-plane* deflection is $w_{in}(t)$ and $w_{in\text{-}bend}$ denotes the deflection at middle point of *AB'*. Based on the beam theory, the deformation meets the equation **Eq. S2**, following 1st-order buckling, and the general solution is

$$w(t) = w_{in-bend}(1 - cosbt),$$
$$n = 1, \qquad b = \frac{2n\pi}{L_1} = \frac{2\pi}{L_1} \quad (S14)$$

Additionally, the strain energy ($U_{in\text{-}bend}$), bending moment ($M_{in\text{-}bend}$) and maximum stress ($\sigma_{in\text{-}bend}$) induced *in-plane* bend, can be considered as **Eq. S15**:

$$U_{in-bend} = \frac{1}{2}\int_0^{L_1} E_m I w''^2(t)dt = \frac{E_m I w_{in-bend}^2 b^4}{4}\int_0^{L_1} 1 + cos2btdt = \frac{E_m \pi^5 r^4 w_{in-bend}^2}{L_1^3},$$
$$M_{in-bend} = E_m I w_{in-bend} b^2 = \frac{4\pi^2 E_m I w_{in-bend}}{L_1^2}, \qquad (S15)$$
$$\sigma_{in-bend} = \frac{M_{in-bend} r}{I} = \frac{4\pi^2 E_m r w_{in-bend}}{L_1^2}$$

Similarly, the additional induced *out-of-plane* bend strain energy ($U_{out-bend}$) and maximum stress ($\sigma_{out-bend}$) follows **Eq. S16**:

$$U_{out-bend} = \frac{E_m \pi^5 r^4 w_{out-bend}^2}{L_1^3},$$
$$\sigma_{out-bend} = \frac{M_{out-bend} r}{I} = \frac{E_m w_{out-bend} \pi^2 r}{L_1^2} \qquad (S16)$$

The chiral structure not only generates global torsion, but also twists the oblique arms by angle θ along its longitudinal direction. We can get

$$\frac{d\theta}{dt} = \frac{T_{rod}}{GJ}, \qquad J = \frac{\pi r^4}{2}, \qquad G = \frac{E_m}{2(1+\nu)} \qquad (S17)$$

where G is the shear modulus of rods. GJ denotes the twisting stiffness. Therefore, by solving **Eq. S17**, we can get the strain energy and shear stress induced by twisting, which is

$$\theta = \frac{T_{rod} L_0}{GJ},$$
$$U_{tor} = \frac{G I_p \theta^2}{2L_0} = \frac{E_m \pi r^4 \theta^2}{8L_0(1+\nu)}, \qquad (S18)$$
$$\tau = \frac{T_{rod} r}{J} = \frac{E_m \theta r}{2(1+\nu)L_0}$$

<u>S3. Analytical model of helix arch rod-based chiral metacells</u>

Through combining the arch rod and chiral structure, the metacell is capable to balance the buffering and energy absorption efficiency. In addition, the additional helix structure can induce out-of-plane bend deformation, which further prevents the buckling and improves stress distribution. According to **Eq. S1**, the K_{eff} decreased significantly due to the increasing rod length, which is considered as

$$L_0 = \frac{\beta}{\pi sin\beta}\sqrt{h_0^2 + \left(\frac{2\pi R}{3}\right)^2},$$
$$\beta = arctan\frac{\sqrt{3}R}{h_0} \qquad (S19)$$

The U_{cpr} can be considered as

$$U_{cpr} = \frac{E_m \pi r^2 s^2}{2L_0},$$
$$s = L_0 - L_1, \qquad L_1 = \sqrt{(h_0 - \varepsilon)^2 + L_0^2 sin^2\beta} \tag{S20}$$

As the arch rod is designed according to the 1/2-order buckling shape, the curvature of rod in the plane of ANB can be considered as **Eq. S21**:

$$w(t) = w_{in-bend}(1 - cosbt),$$
$$n = \frac{1}{2}, \qquad b = \frac{2n\pi}{L_1} = \frac{\pi}{L_1} \tag{S21}$$

Therefore, the $U_{in\text{-}bend}$ can be considered as

$$U_{in-bend} = \frac{1}{2}\int_0^{L_1} E_m I w''^2(t)dt = \frac{E_m \pi^5 r^4 w_{in-bend}^2}{16L_1^3} \tag{S22}$$

where $w_{in\text{-}bend}$ referred to the maximum deflection, $2a_{in}$, in the plans of *ABN*. Besides, the helix rod in CAH metacells forms an arch structure in the plane of AO_1N, following 1st-order buckling, therefore the induced $U_{out\text{-}bend}$ can be considered as **Eq. S23**:

$$n = 1, \qquad b = \frac{2\pi}{L_1},$$
$$U_{out-bend} = \frac{1}{2}\int_0^{L_1} E_m I w''^2(t)dt = \frac{E_m \pi^5 r^4 w_{out-bend}^2}{L_1^3} \tag{S23}$$

where $w_{out\text{-}bend}$ referred to the maximum deflection, a_{out}, in the plans of AO_1N. According to **Eq. S18**, we can get U_{tor},

$$U_{tor} = \frac{GI_p\theta^2}{2L_0} = \frac{E_m \pi r^4 \theta_r^2}{8L_0(1+\nu)} \tag{S24}$$

Different from the CL metacells, the CAH metacell occurs helix deformation due to *out-of-plane* bend, which significantly effects torsion angle θ. As shown in **Fig. S32**, the revised θ (θ_r) reads

$$\theta_r = \theta - \varphi,$$
$$\varphi = \frac{\pi}{2} + arctan\frac{w_{out-bend}}{Rsin(\frac{2\pi+\gamma}{3})} \tag{S25}$$

Supplementary Figures

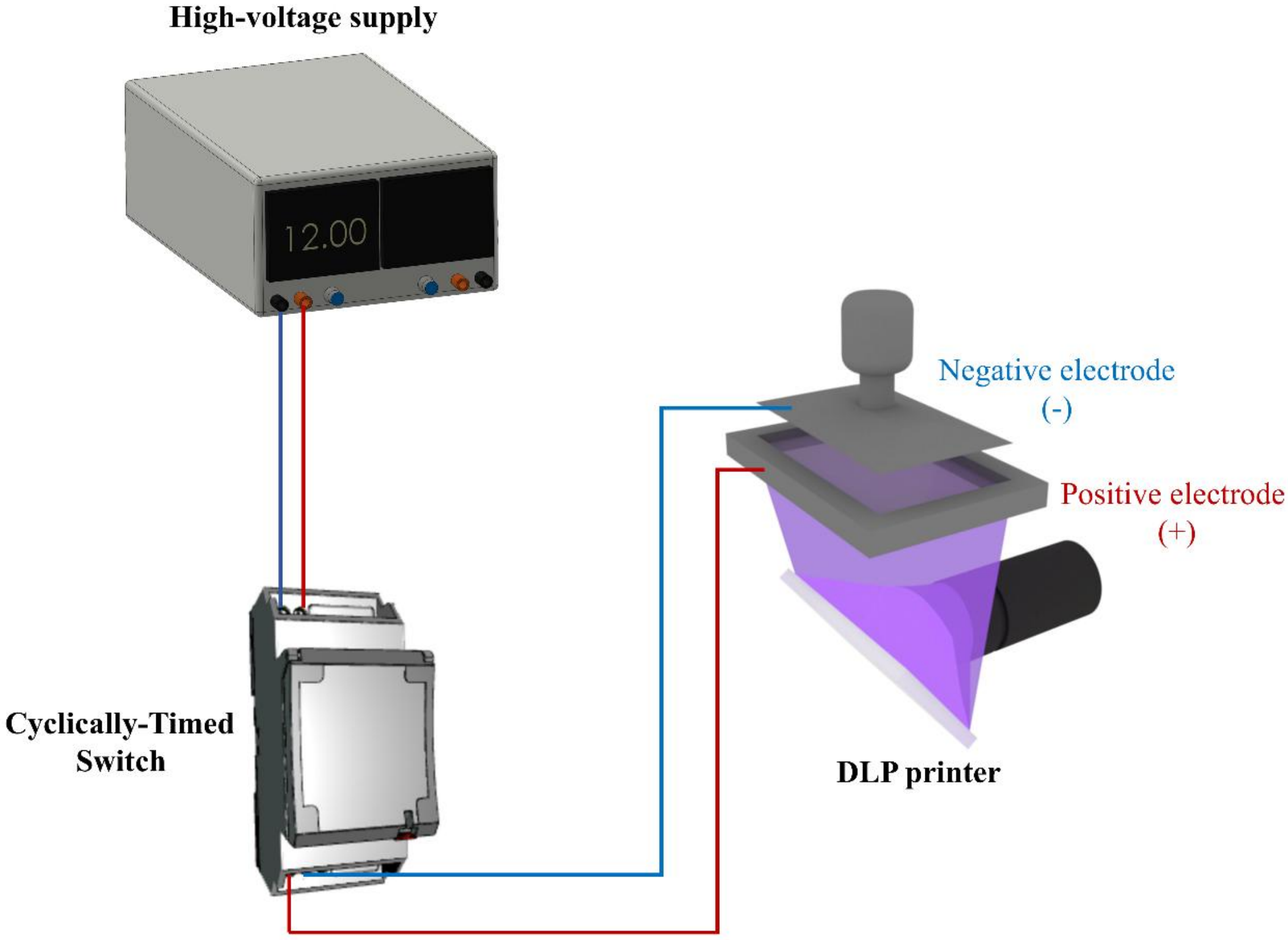

Fig. S1.
Construction of timing system, including a power supply and a cyclically-timed switch for controlling the electric field pulse interval during electric-field-assisted DLP printing process.

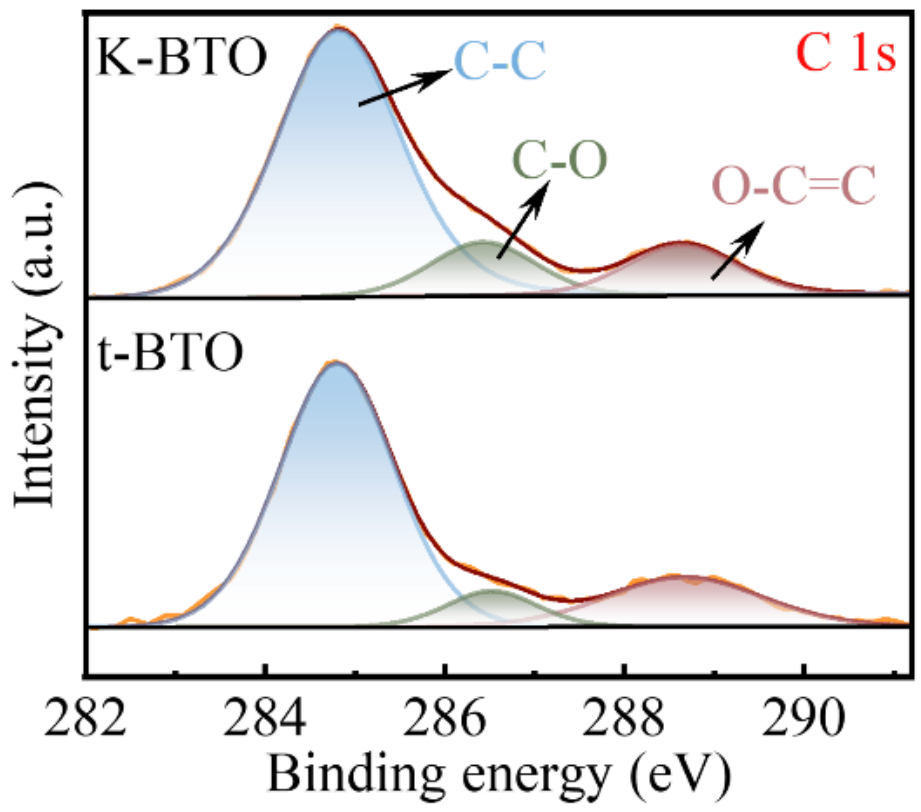

Fig. S2.
C1s spectra of t-BTO and K-BTO.

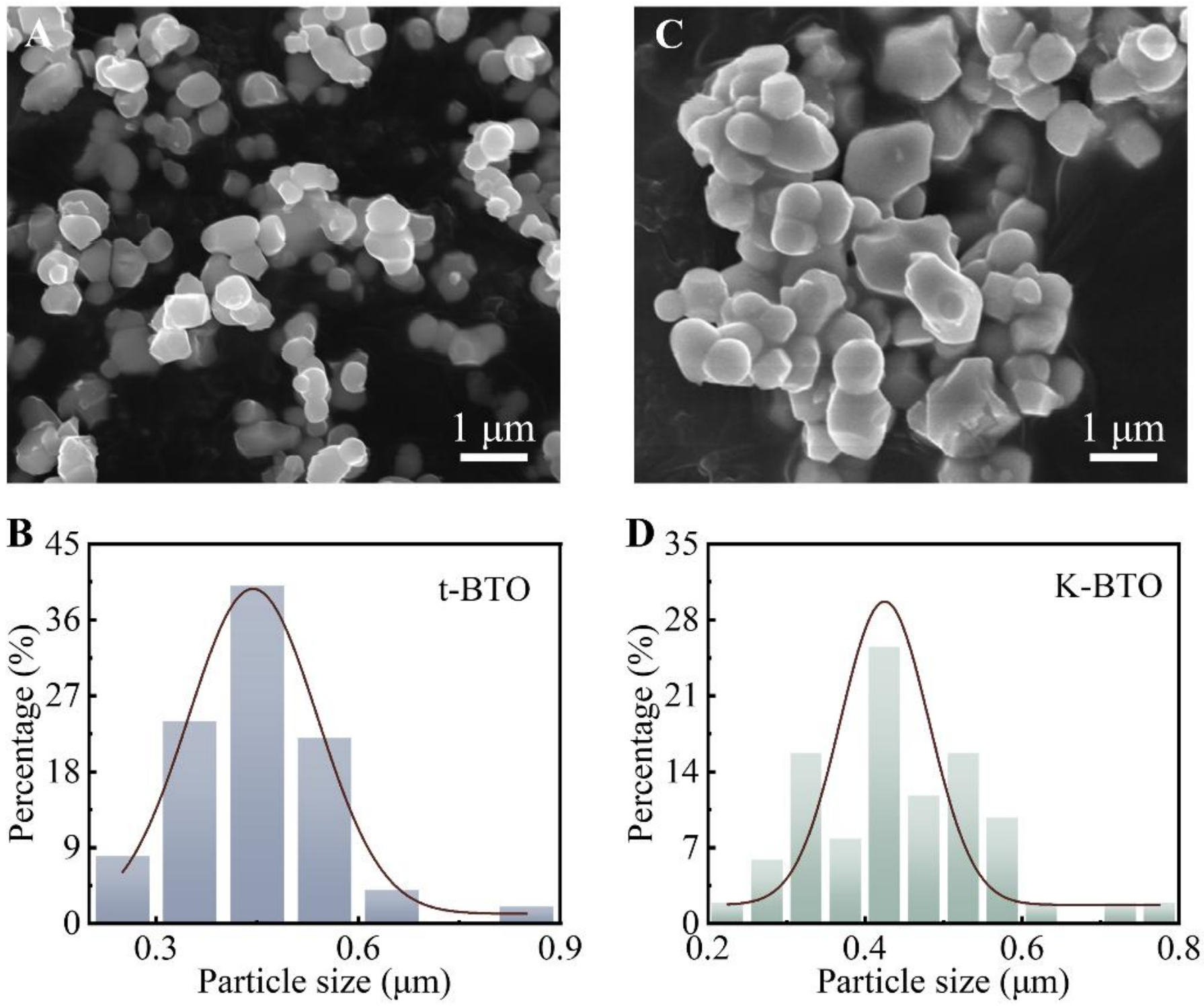

Fig. S3.
(A, C) SEM morphologies and **(B, D)** particle size distribution of t-BTO and K-BTO.

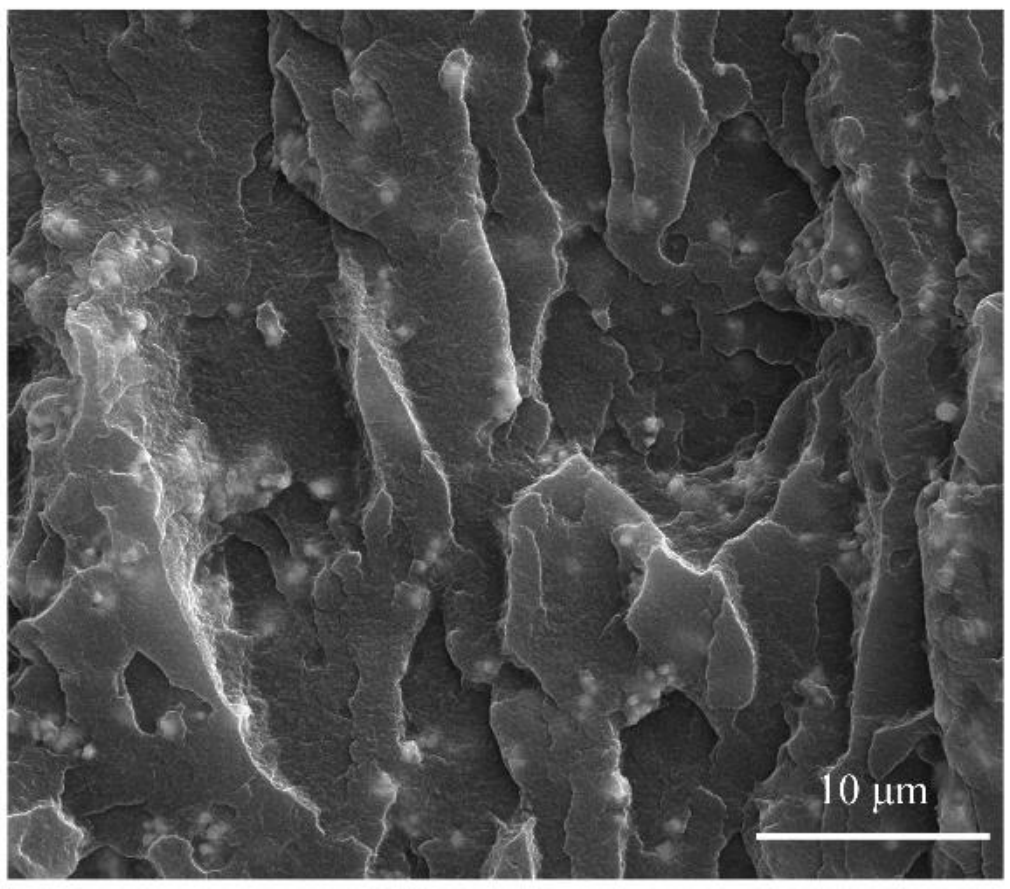

Fig. S4.
Dispersion morphologies of K-BTO in the PLUG matrix.

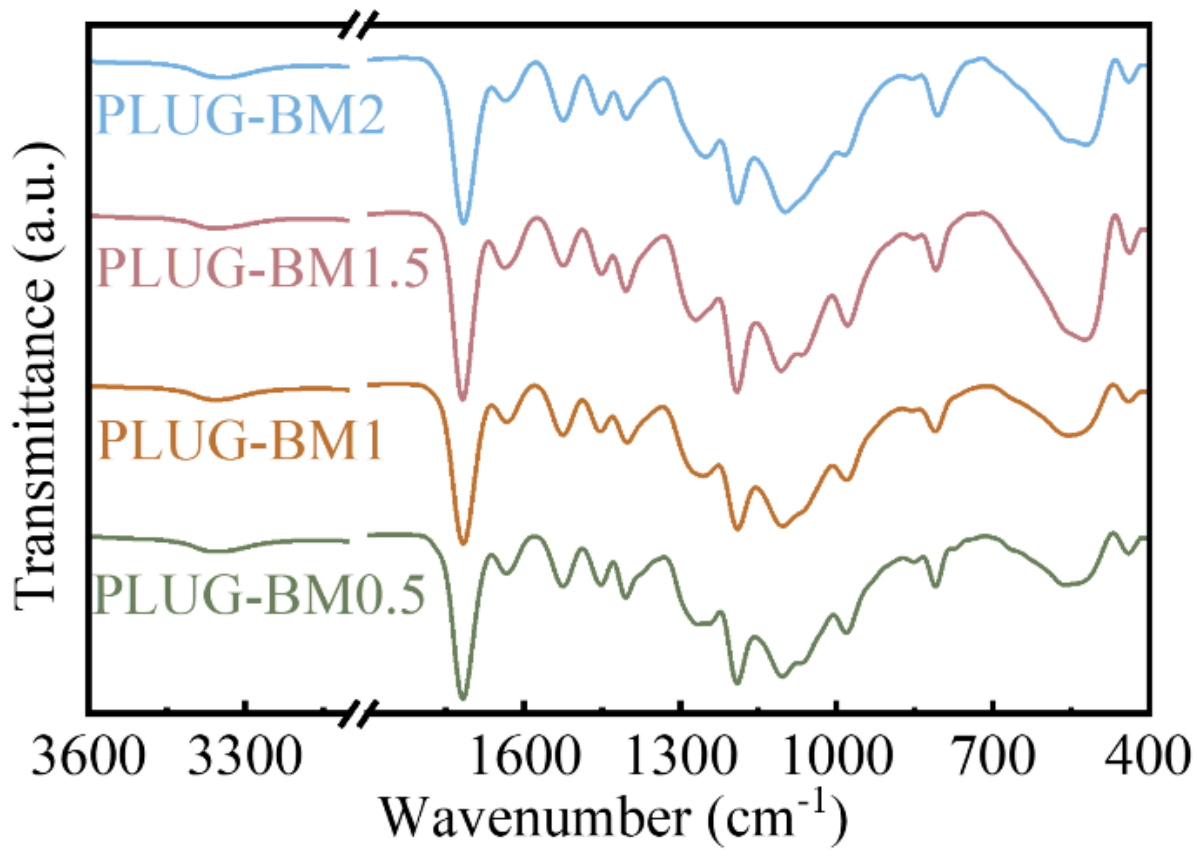

Fig. S5.

FT-IR spectra of PLUG-BM0.5, PLUG-BM1, PLUG-BM1.5 and PLUG-BM2.

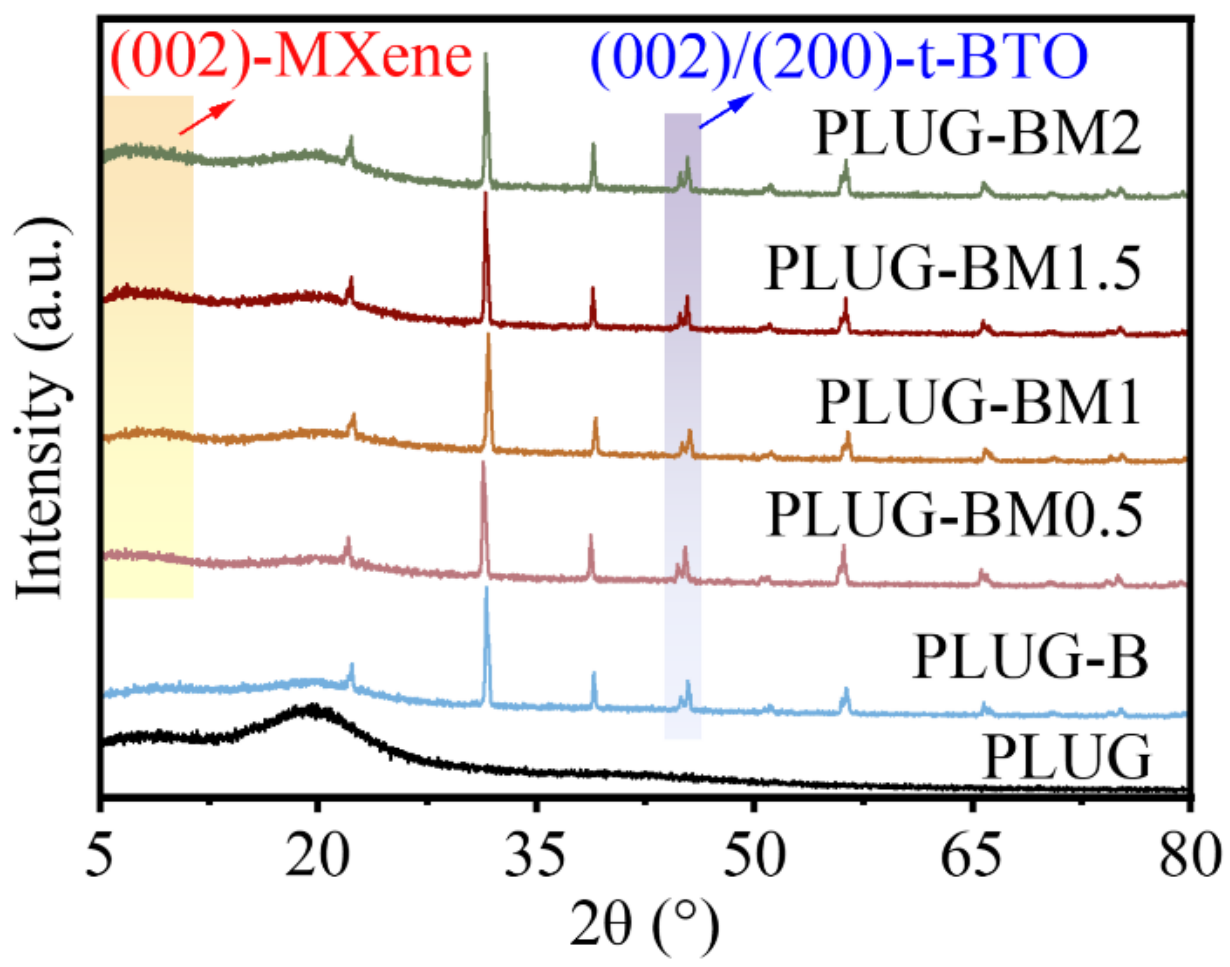

Fig. S6.
XRD spectra of PLUG-BM0.5, PLUG-BM1, PLUG-BM1.5 and PLUG-BM2.

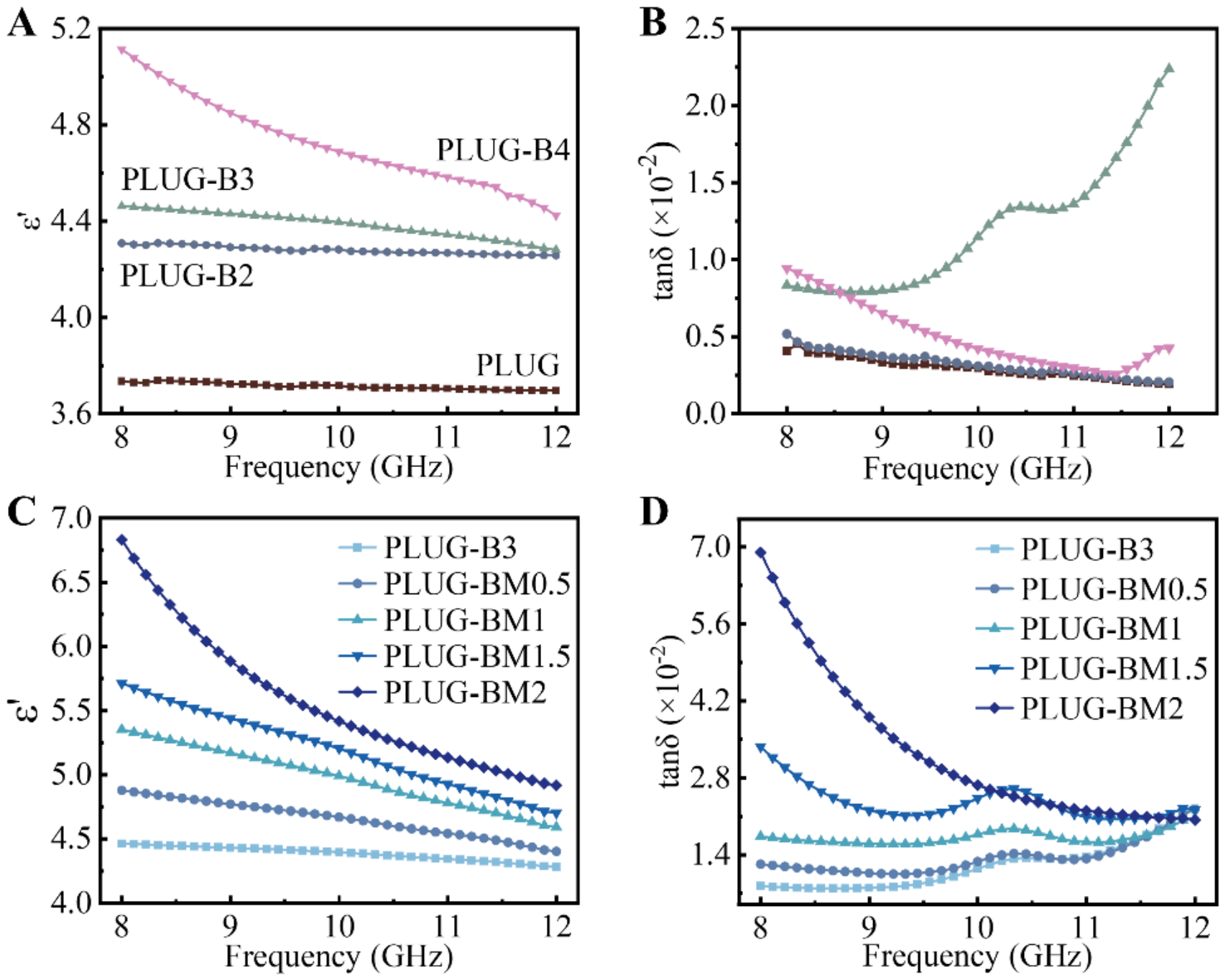

Fig. S7.

(A) Real dielectric coefficient and **(B)** dielectric loss (*tanδ*) of PLUG, PLUG-B2, PLUG-B3 and PLUG-B4. **(C)** Real dielectric coefficient and **(D)** dielectric loss (*tanδ*) of the PLUG-B3 with different MXene content.

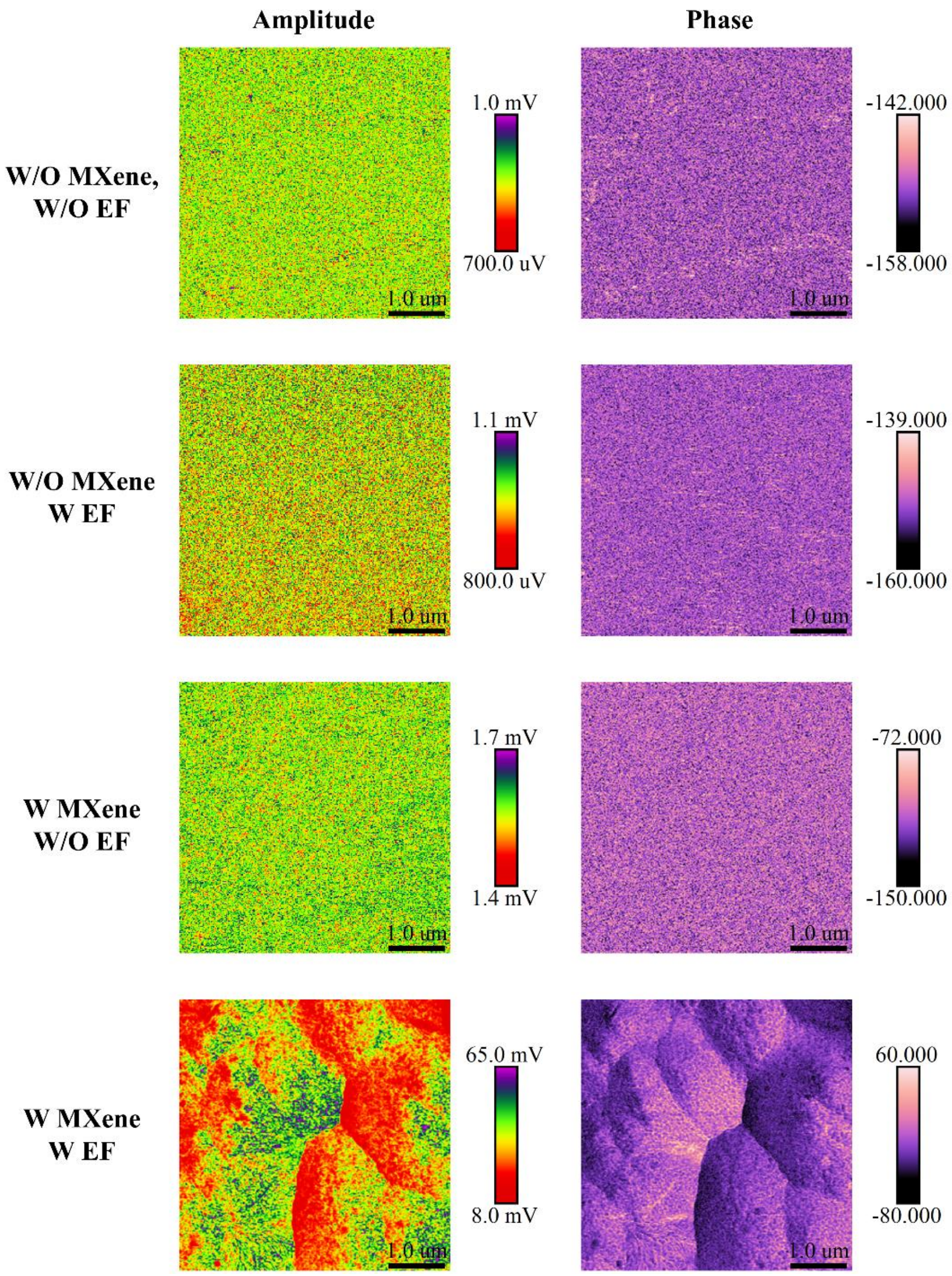

Fig. S8.

PFM results of the PLUG-B without *in-situ* EF (**W/O MXene, W/O EF**), PLUG-B with 1.2 MV/m *in-situ* EF (**W/O MXene, W EF**), PLUG-BM1.5 without *in-situ* EF (**W MXene, W/O EF**) and PLUG-BM1.5 with 1.2 MV/m *in-situ* EF (**W MXene, W EF**).

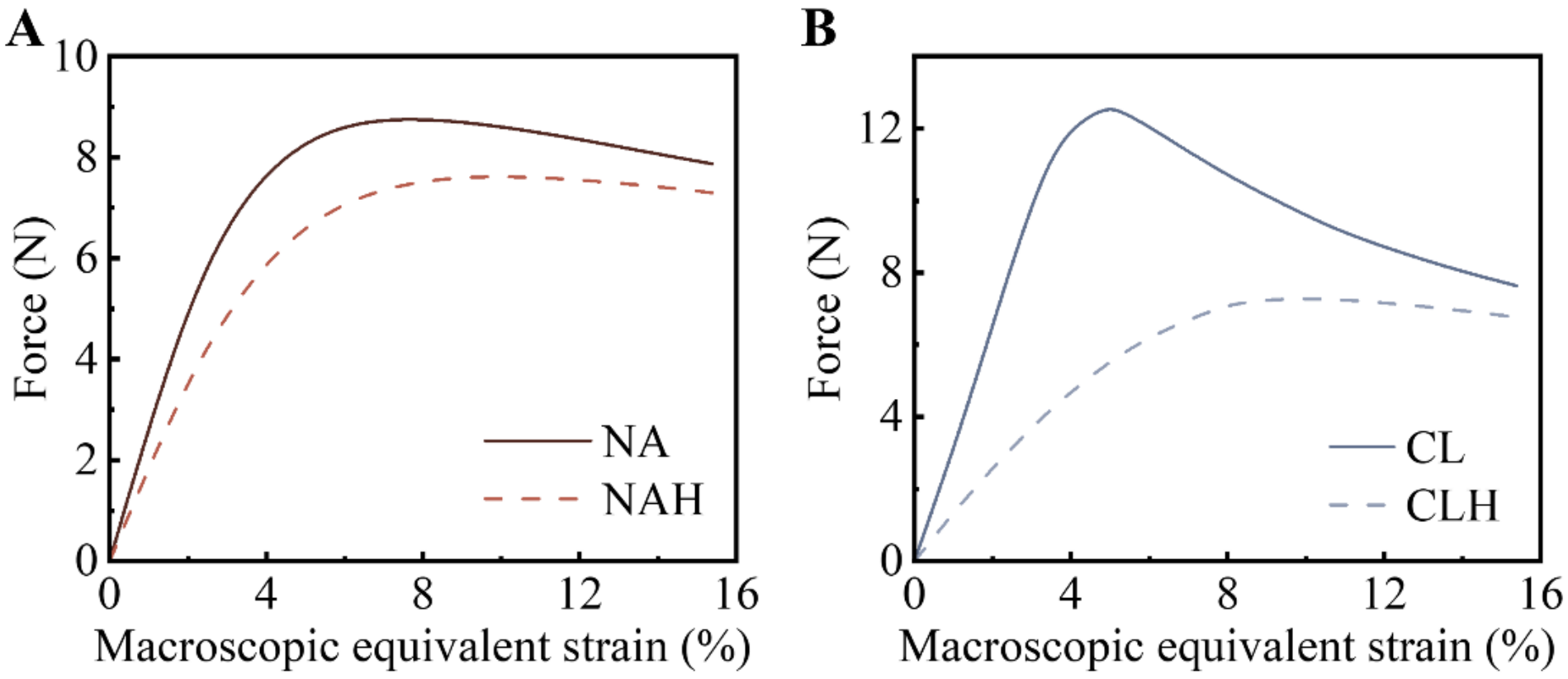

Fig. S9.

Force-strain curves of **(A)** NA and NAH metacells, as well as **(B)** CL and CLH metacells.

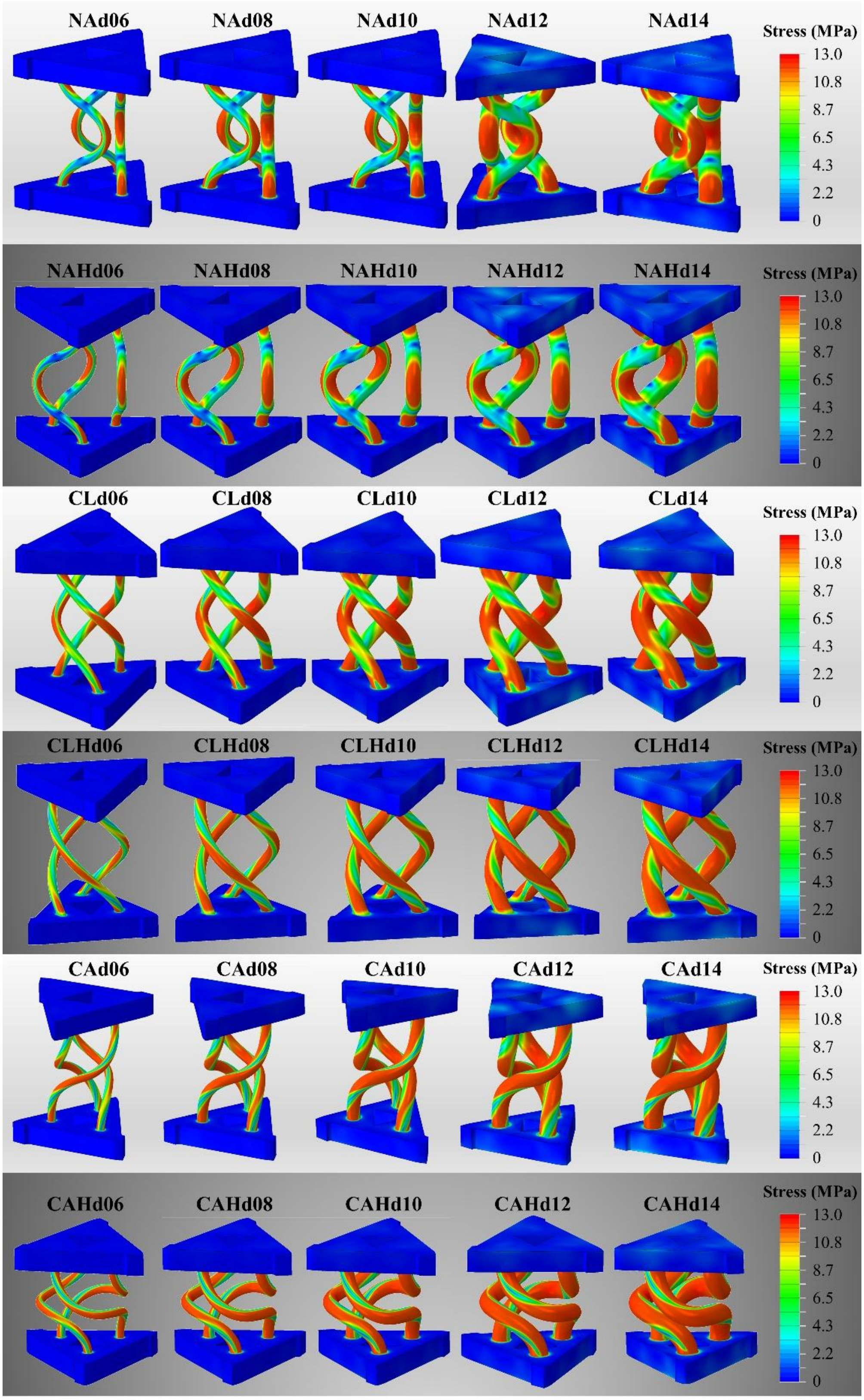

Fig. S10.

The effect of rod diameter on stress distribution of NA, NAH, CL, CLH, CA and CAH metacells.

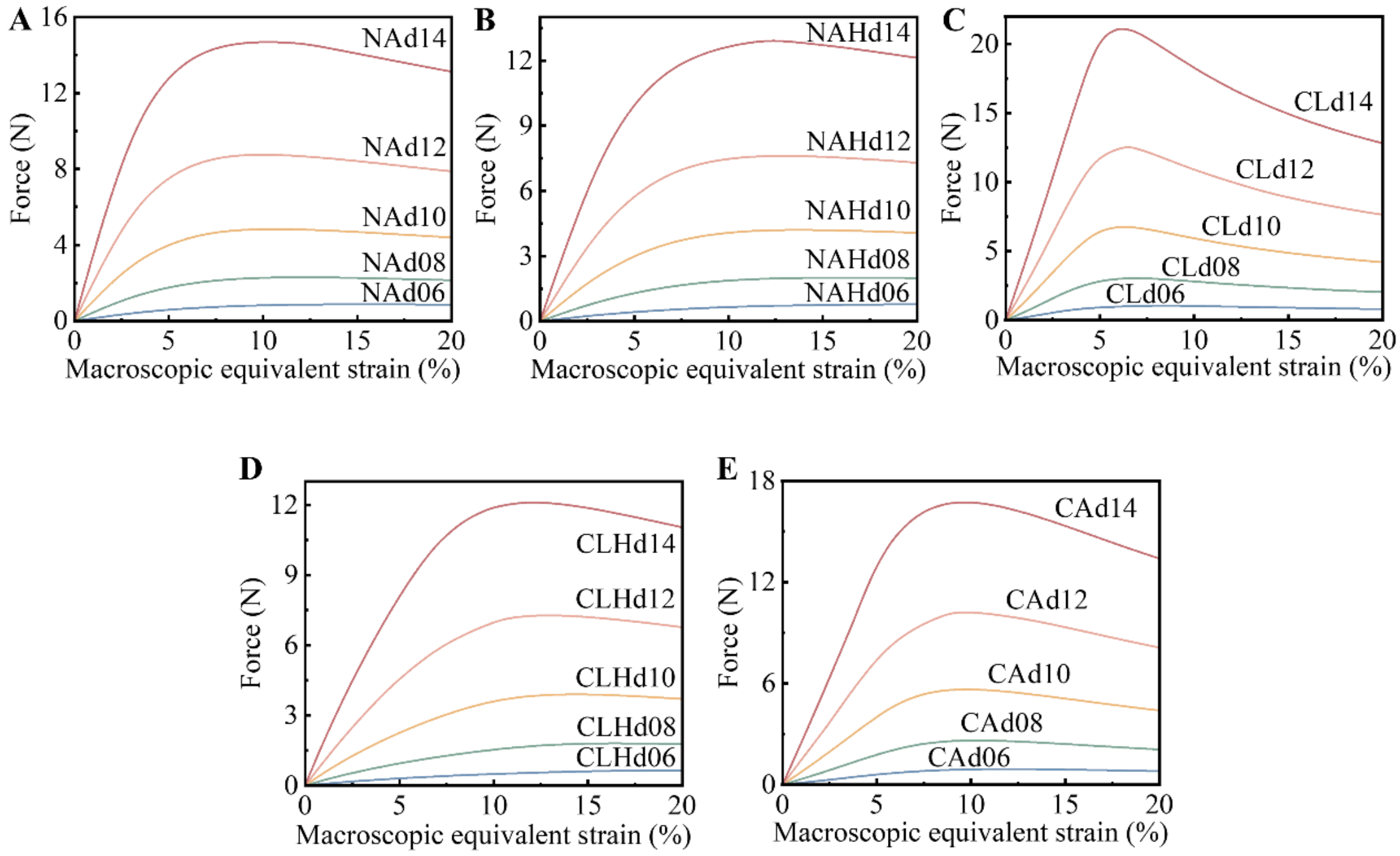

Fig. S11.

Force-strain curves of **(A)** NA, **(B)** NAH, **(C)** CL, **(D)** CLH and **(E)** CA metacells with varying rod diameters.

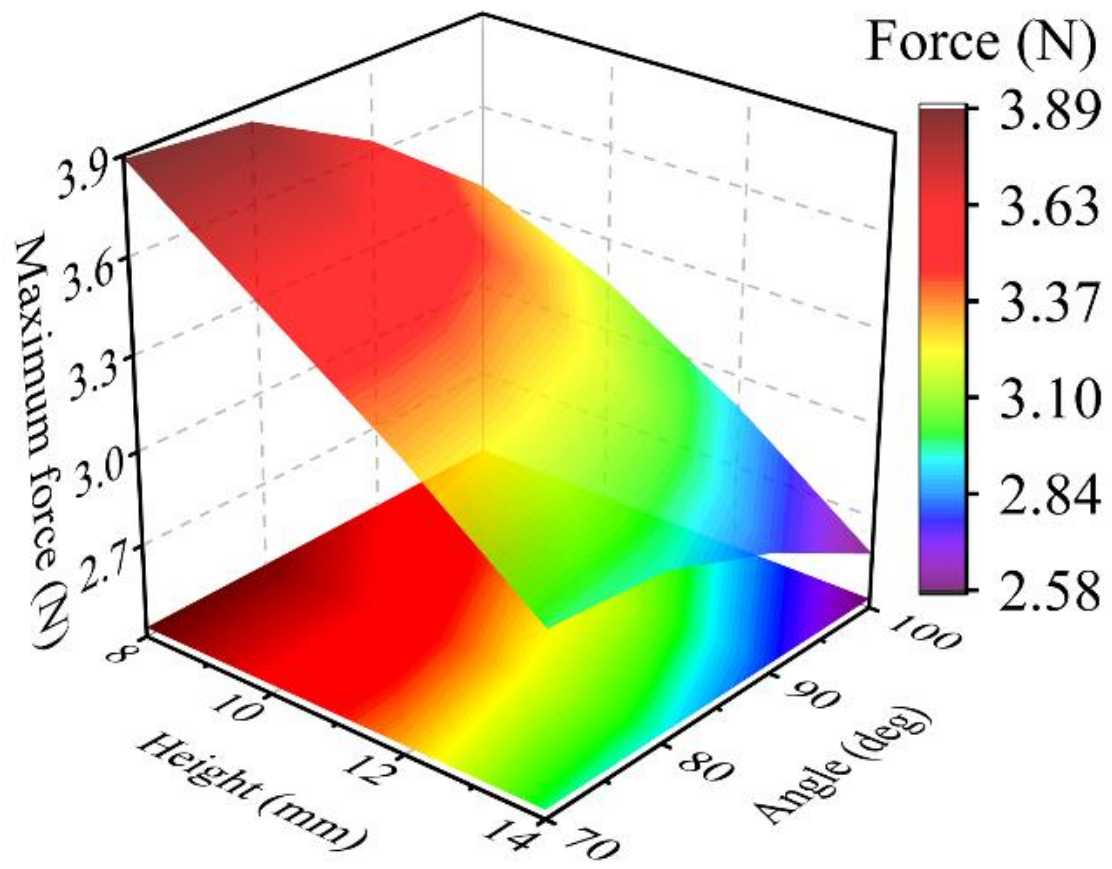

Fig. S12.

The influence of cell height and rod angle (β) on maximum reaction force, representing the bearing capacity.

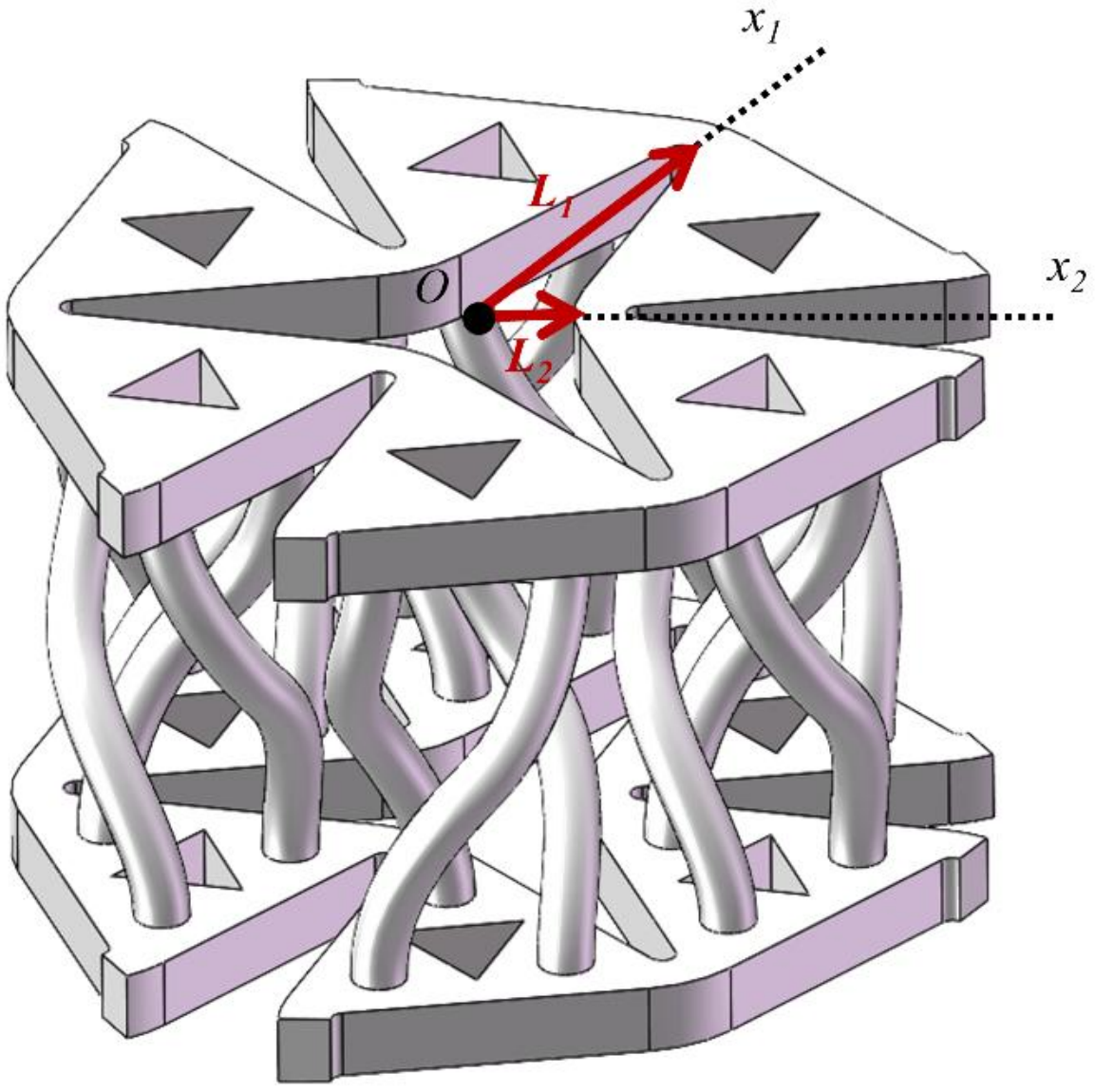

Fig. S13.

The structural parameters of k-CAH metamaterials, where L_1 represents the long inner dimension as well as L_2 represents the short inner dimension.

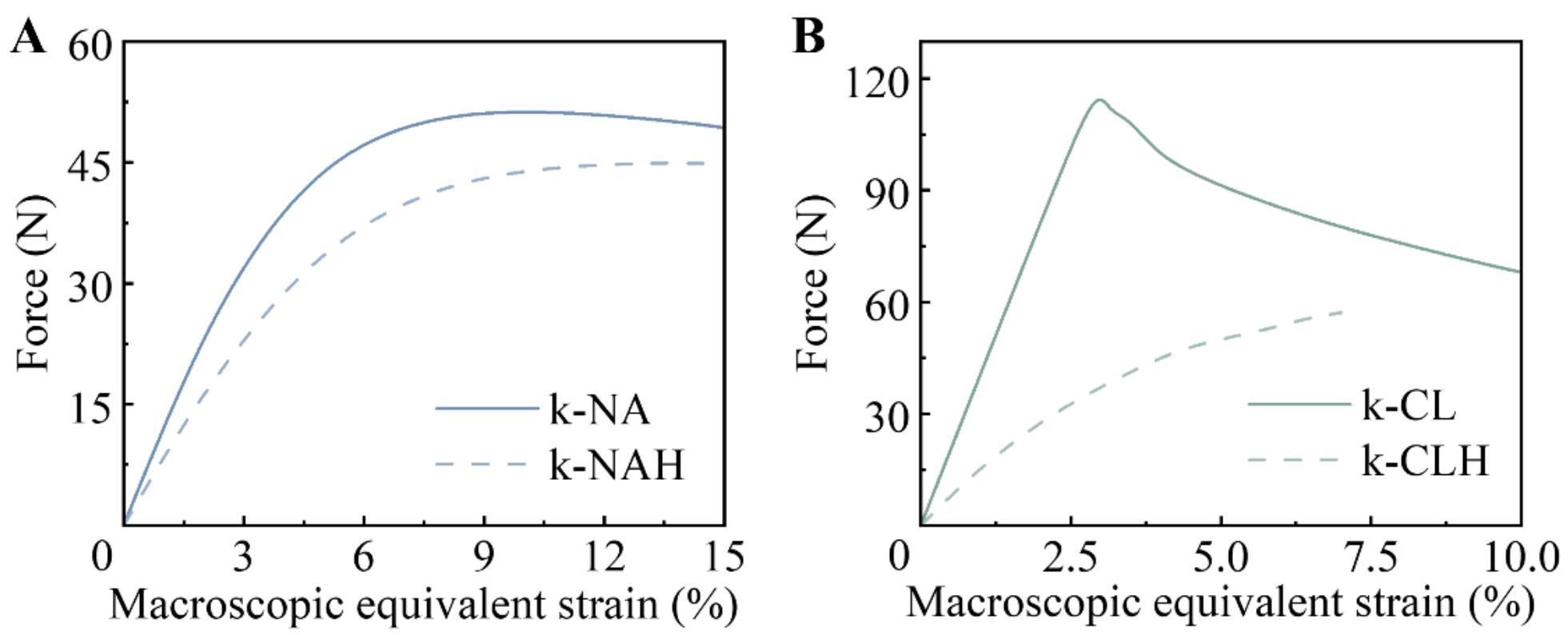

Fig. S14.

Force-strain curves of **(A)** k-NA and k-NAH metamaterials, as well as **(B)** k-CL and k-CLH metamaterials.

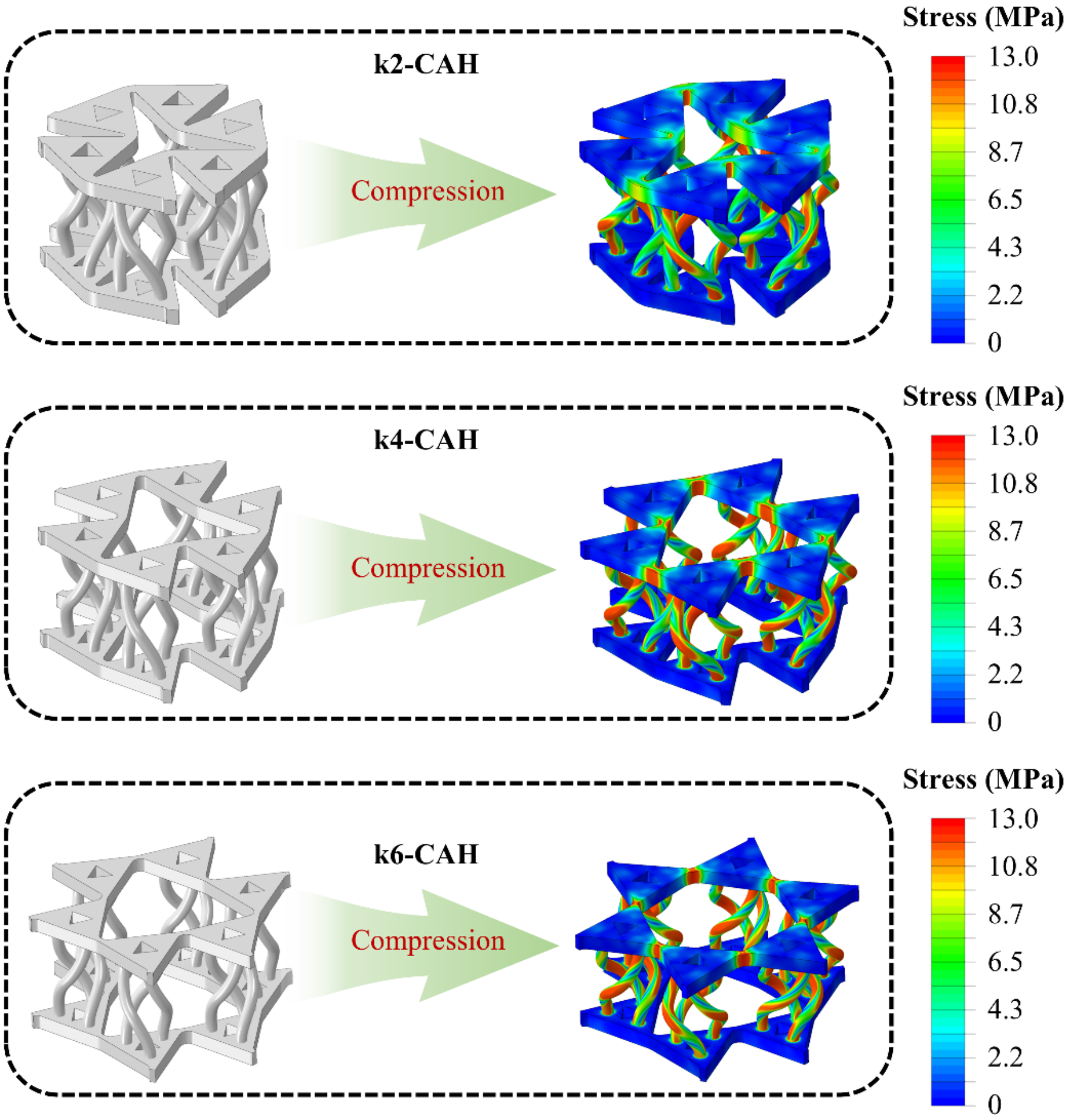

Fig. S15.

Simulated compression results of k-CAH metamaterials with varying rod diameters: k2-CAH, k4-CAH and k6-CAH.

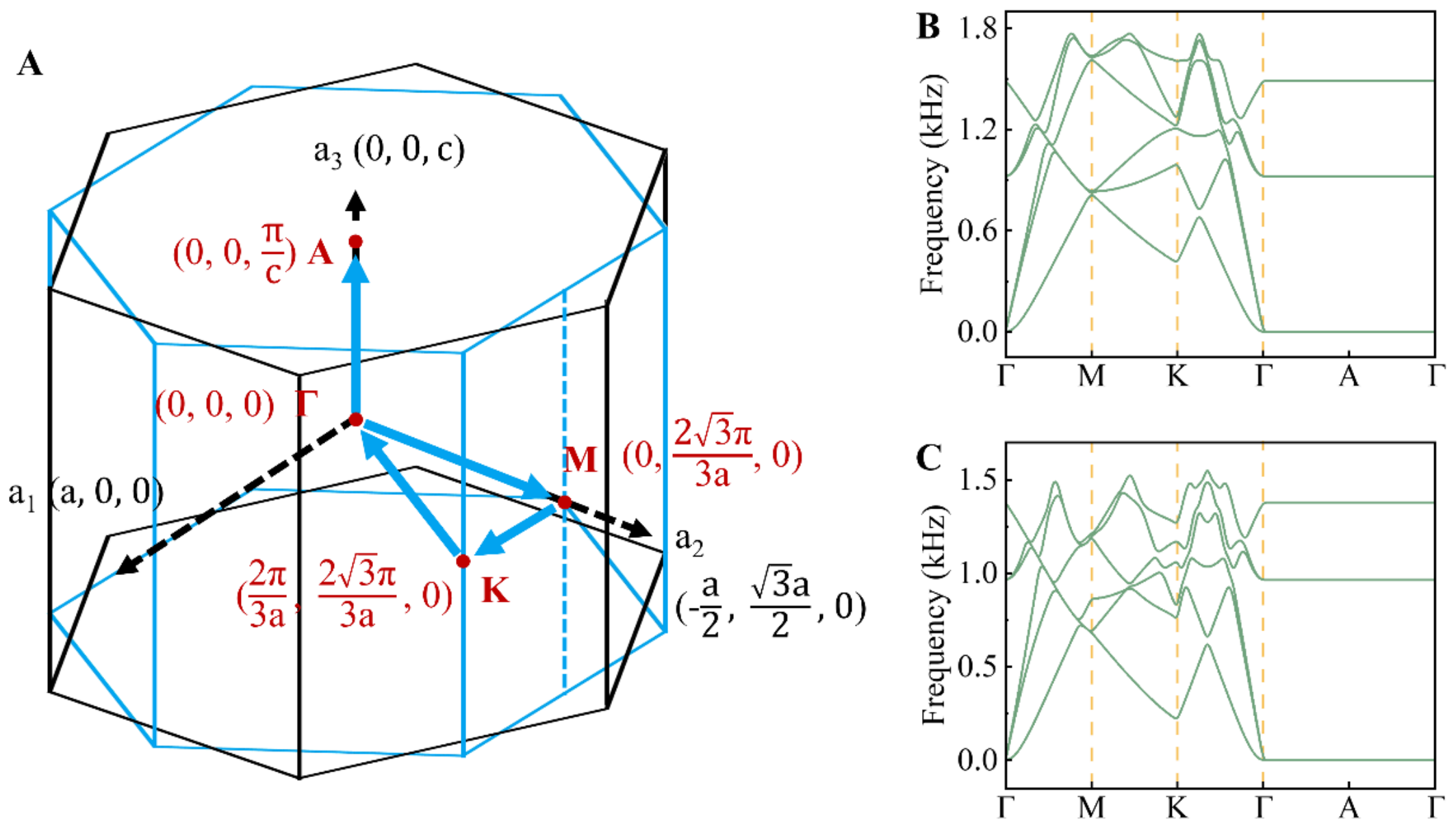

Fig. S16.

(A) Reciprocal lattice vector of hexagonal lattice. Phonon spectra of **(B)** k4-CAH and **(C)** k6-CAH metamaterials simulated via COMSOL.

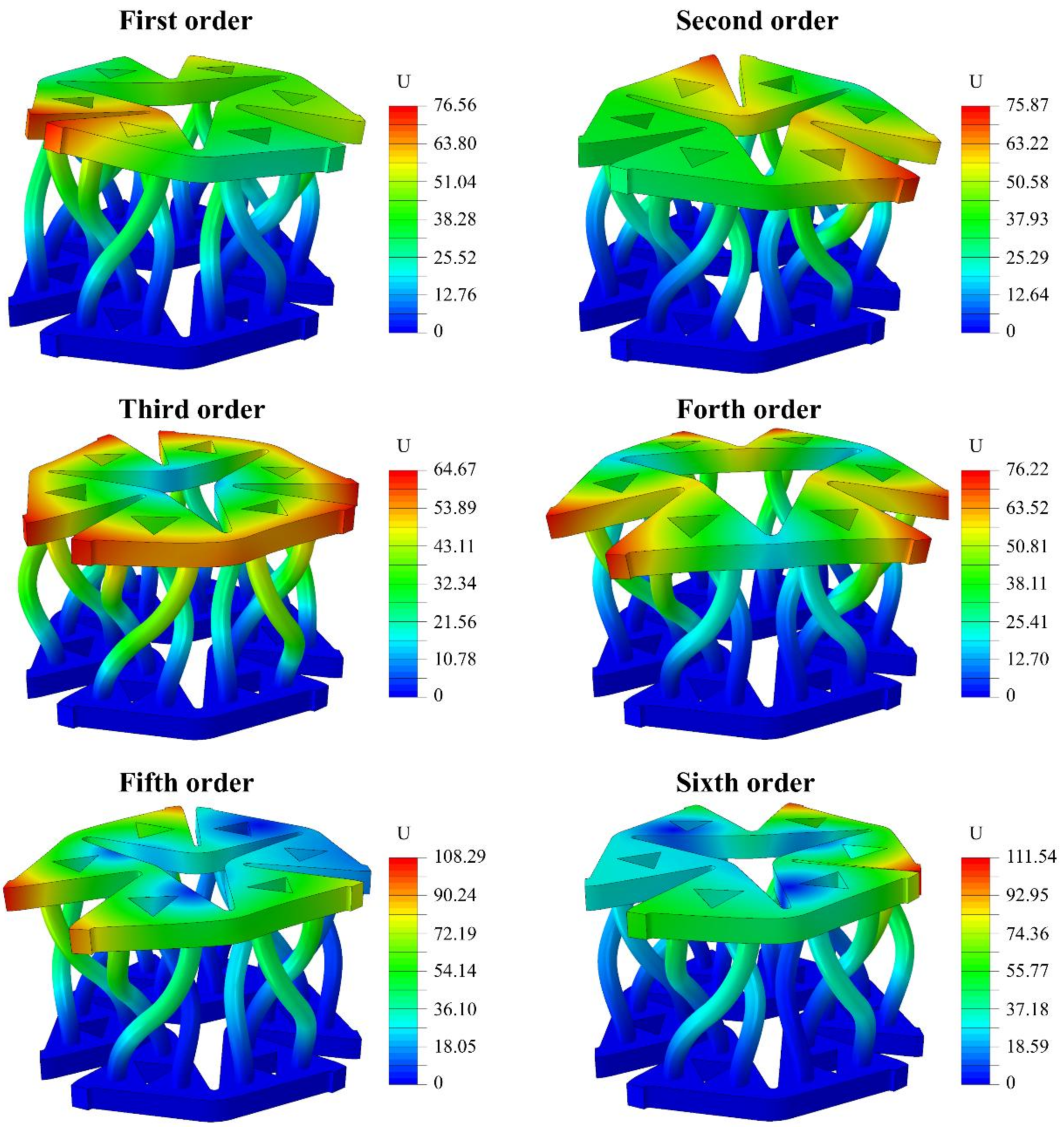

Fig. S17.

Varying-order natural frequency of k2-CAH metamaterials.

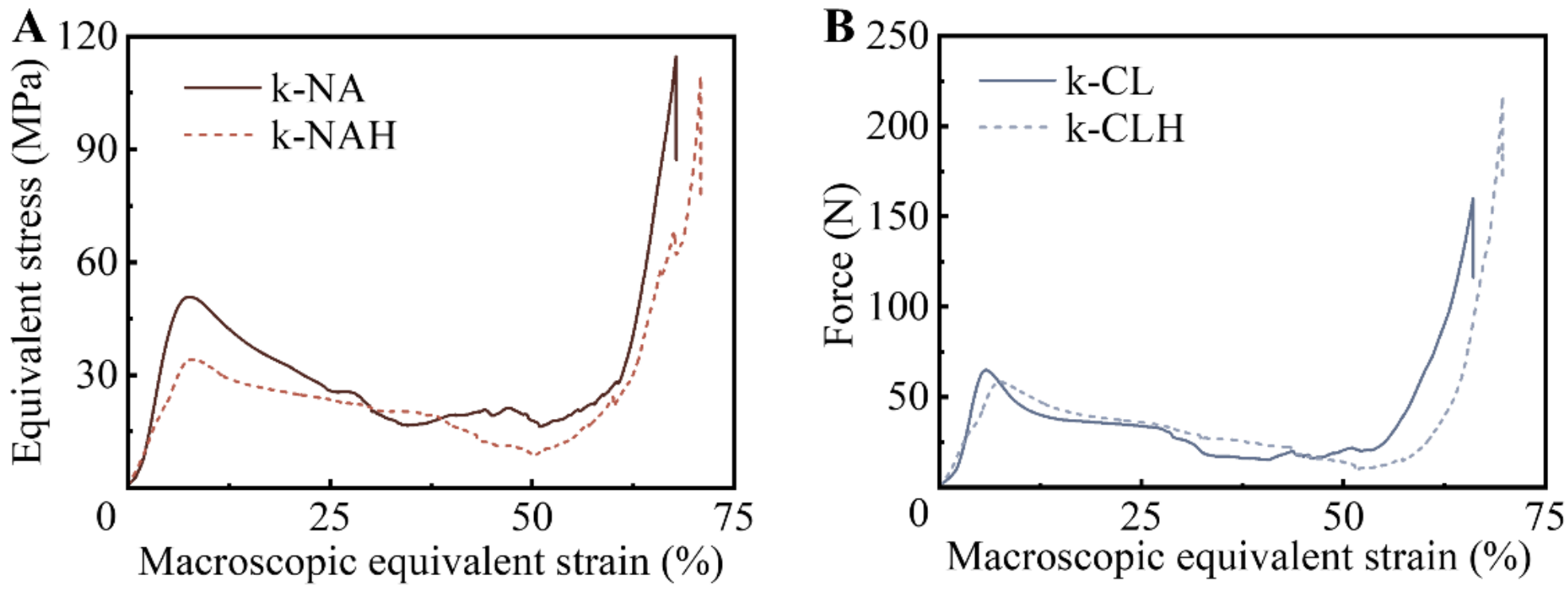

Fig. S18.

The quasi-static compression force-strain curves of **(A)** k-NA and k-NAH metamaterials, as well as **(B)** k-CL and k-CLH metamaterials.

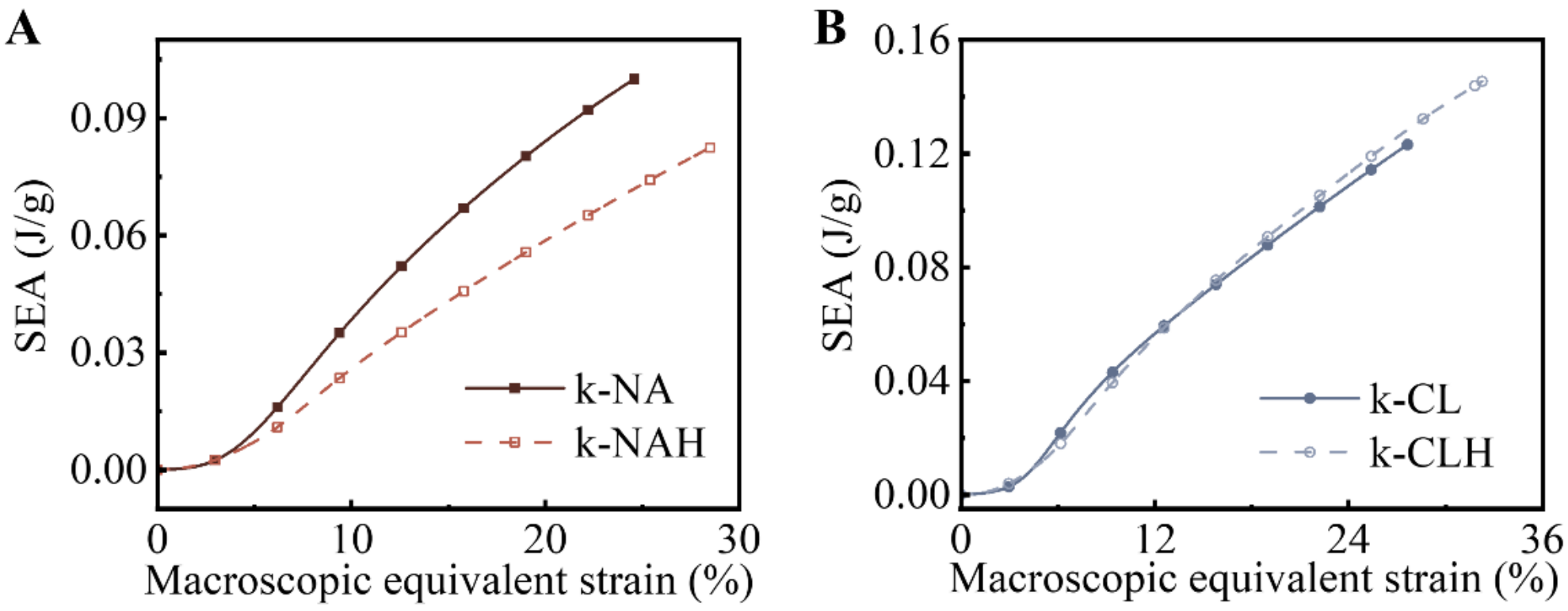

Fig. S19.

Specific energy absorption (SEA)-strain curves of **(A)** k-NA and k-NAH metamaterials, as well as **(B)** k-CL and k-CLH metamaterials.

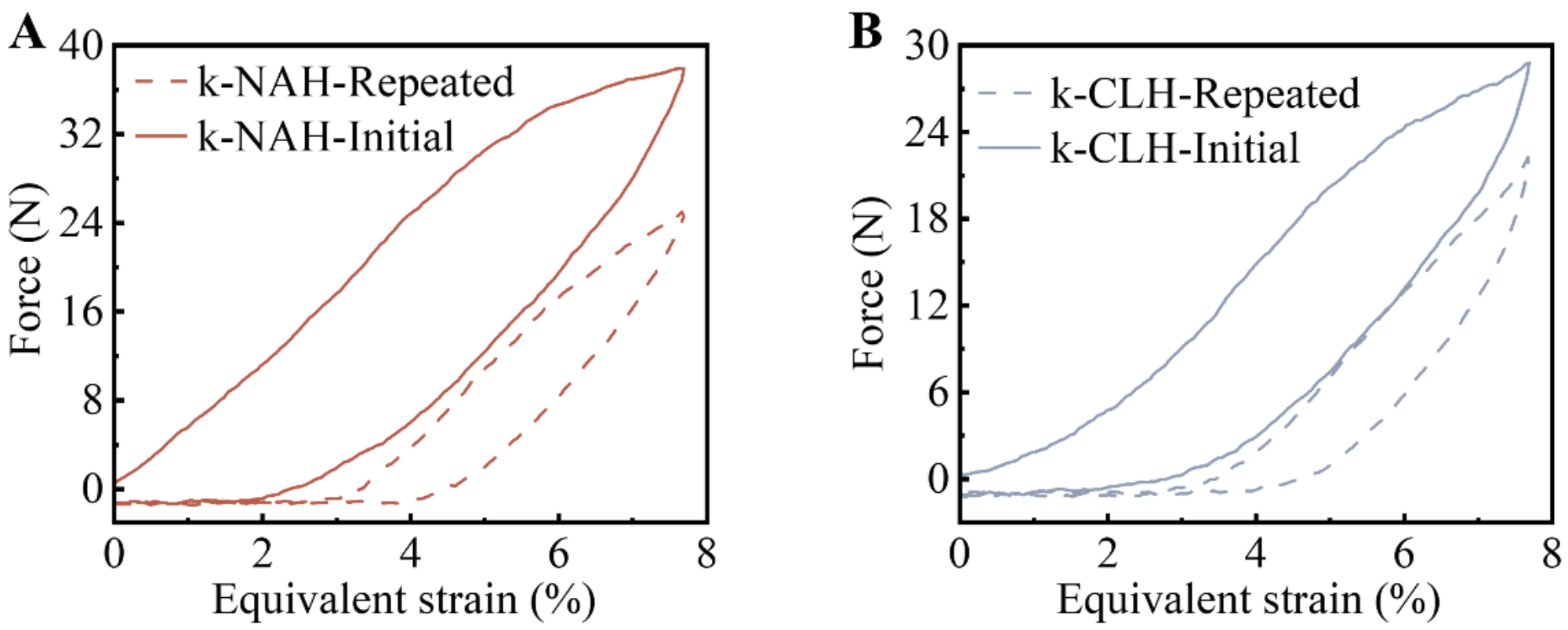

Fig. S20.

The cyclical force-strain curves, at the initial stage and after 200 cycles, of **(A)** k-NAH and **(B)** k-CLH metamaterials.

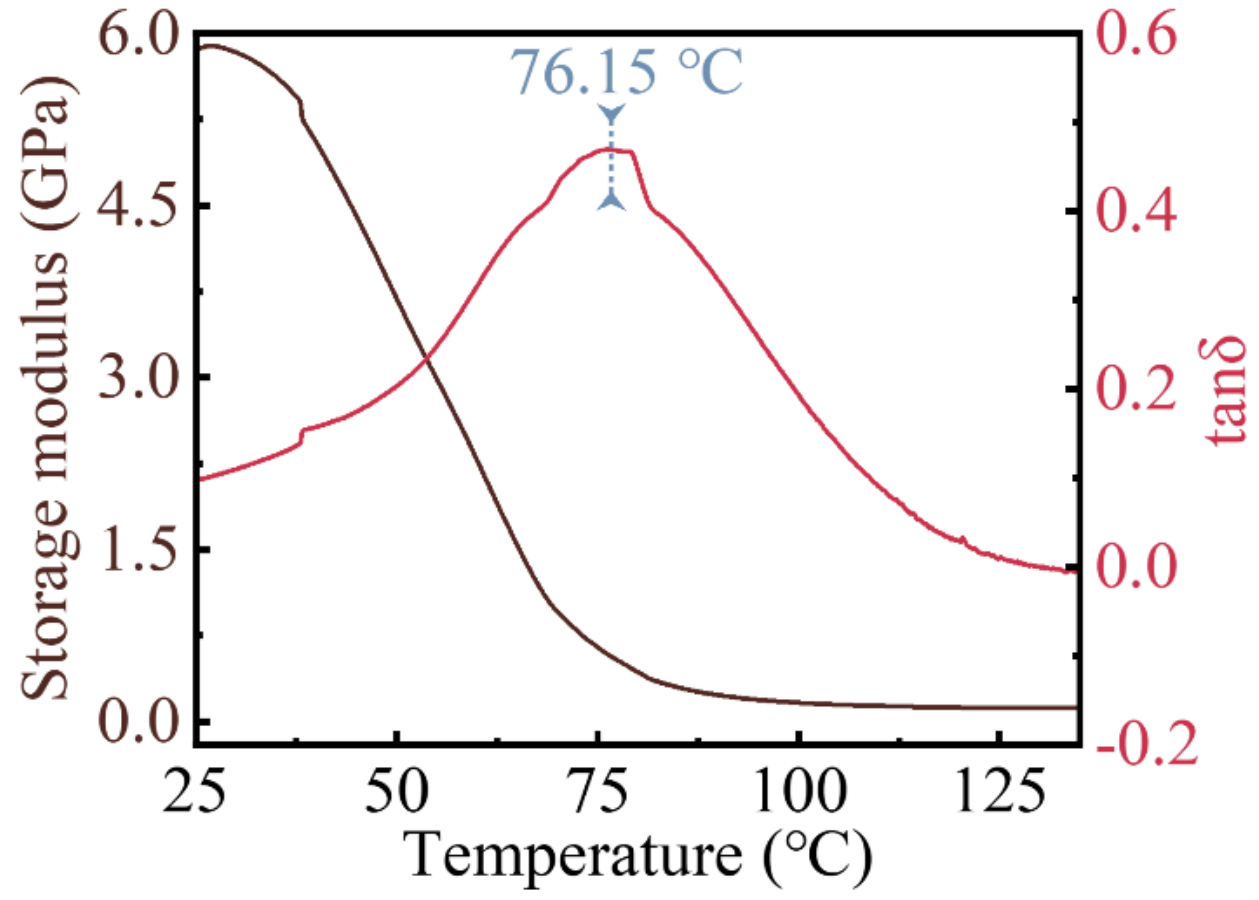

Fig. S21.

Dynamic mechanical analysis (DMA) curves of PLUG-BM1.5 composites for 4D-printed metamaterials.

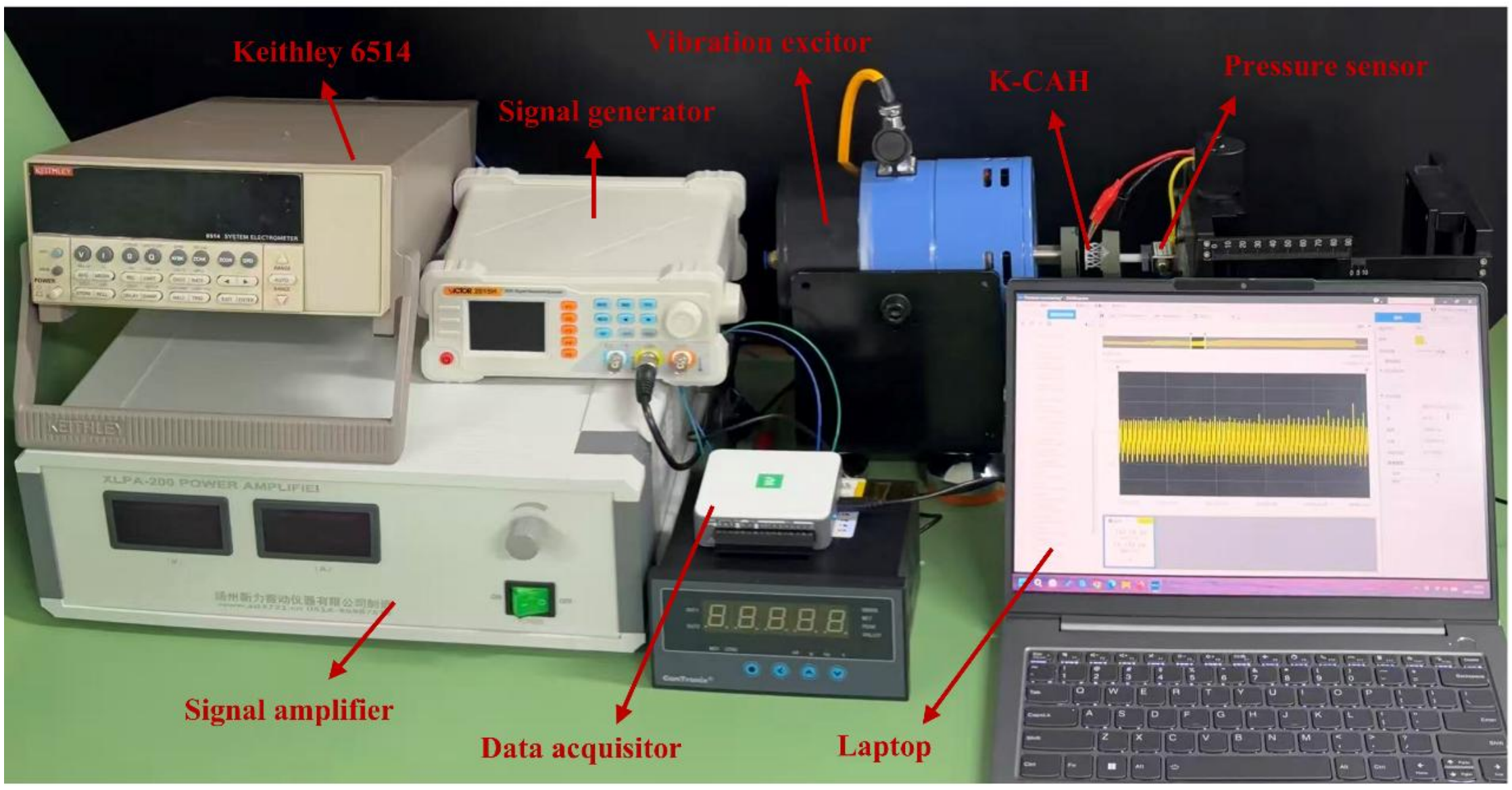

Fig. S22.

Experimental platform for energy harvesting and real-time impact monitoring, including Keithley 6514 electrometer, signal generator, signal amplifier, data acquisitor, vibration excitor and pressure sensor.

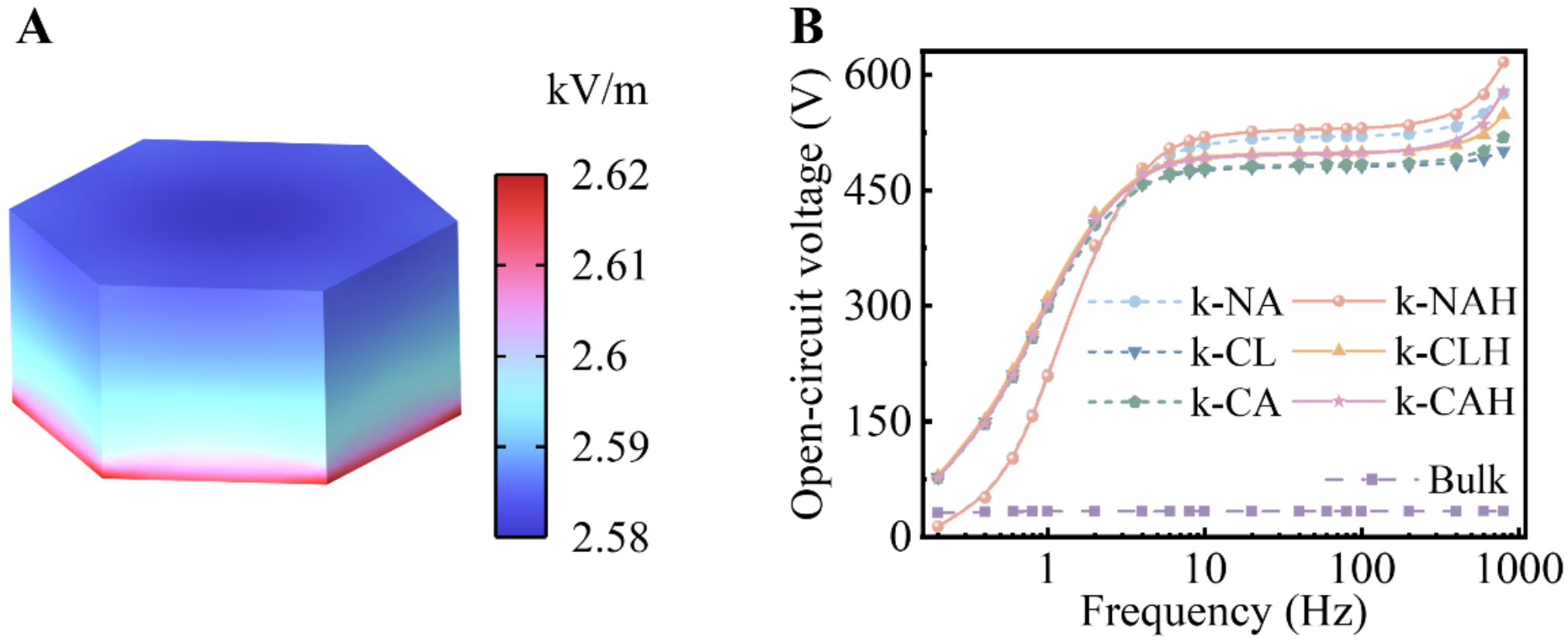

Fig. S23.

(A) The electric field strength nephogram of hexagon bulk. **(B)** Open-circuit voltage of varying structures at different frequency.

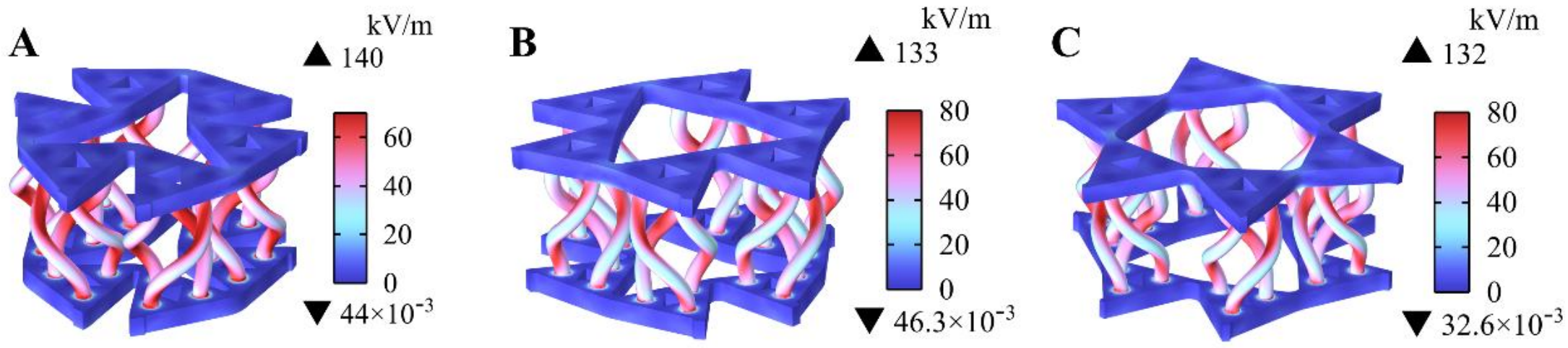

Fig. S24.

The nephogram of **(A)** k2-CAD, **(B)** k4-CAH and **(C)** k6-CAH at load frequency of 980 Hz (2 N).

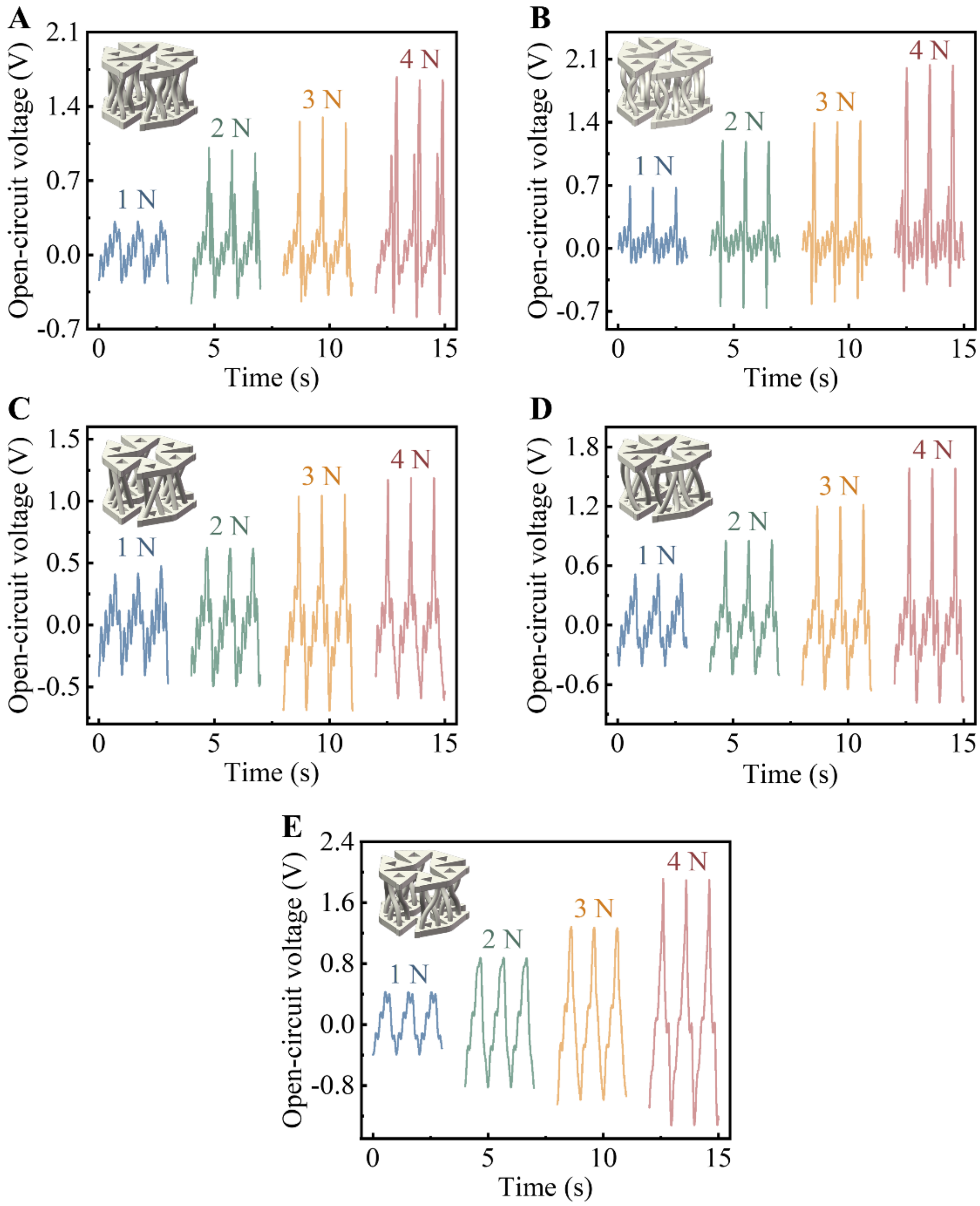

Fig. S25.

Open-circuit voltage, under varying load, of **(A)** k-NAH, **(B)** k-CLH and **(C)** k-CA metamaterial.

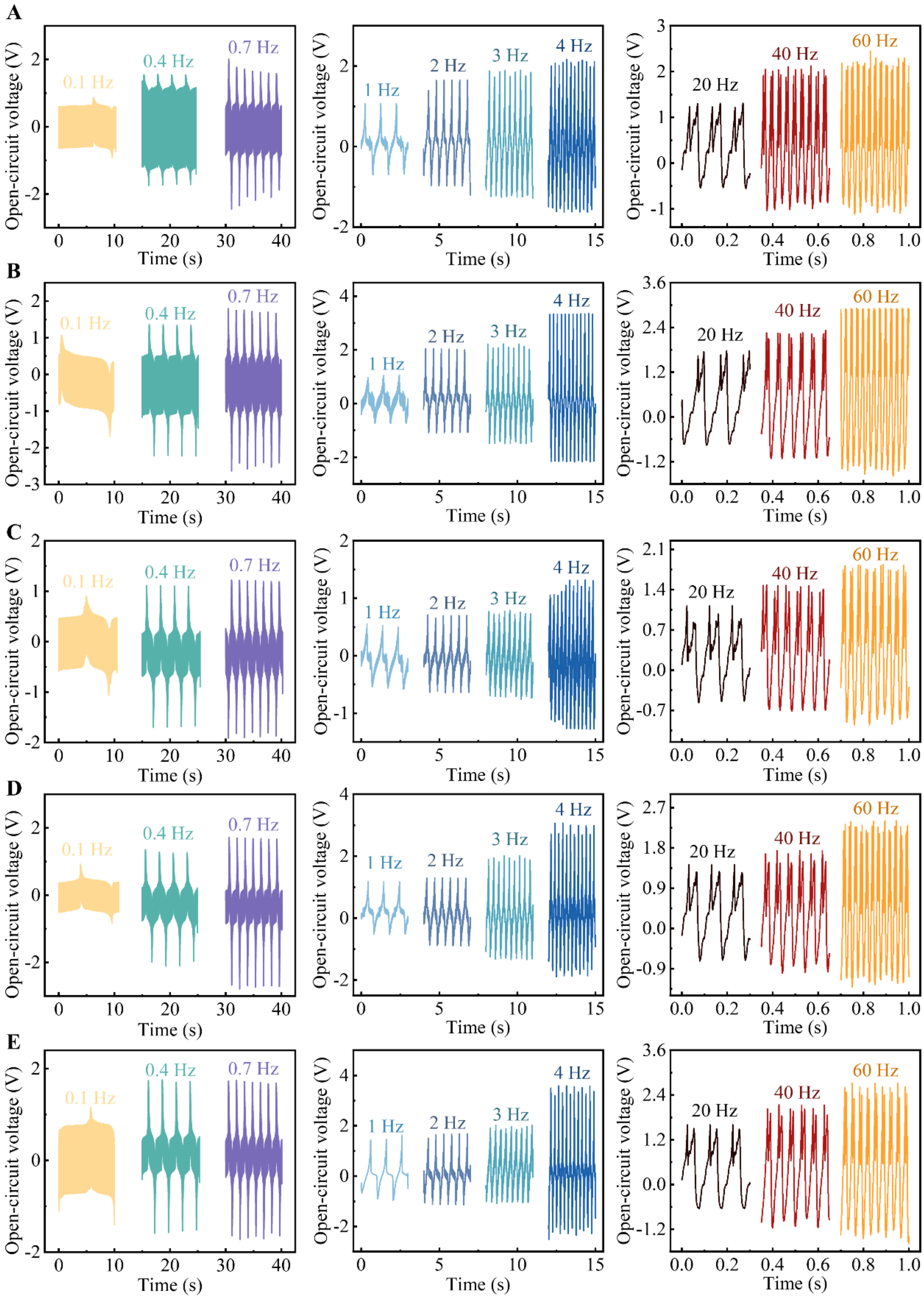

Fig. S26.

Open-circuit voltage of **(A)** k-NA, **(B)** k-NAH, **(C)** k-CL, **(D)** k-CLH and **(E)** k-CA metamaterials at low frequency (0.1-0.7 Hz, 3 N), moderate frequency (1-4 Hz, 3 N) and high frequency (20-40 Hz, 3 N).

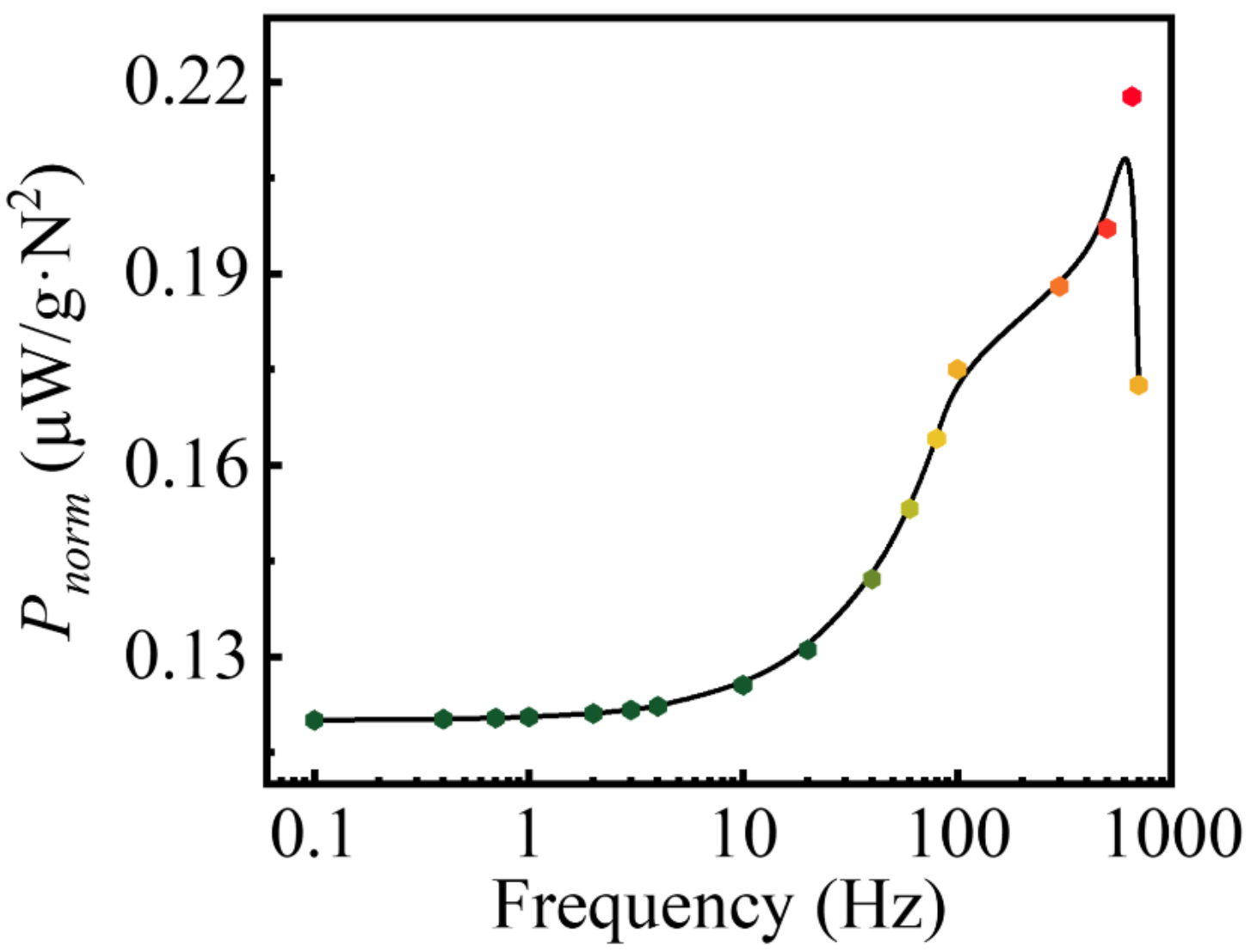

Fig. S27.

Simulated power density-frequency curve of the k-CAH metamaterial with an external resistance of 9 MΩ.

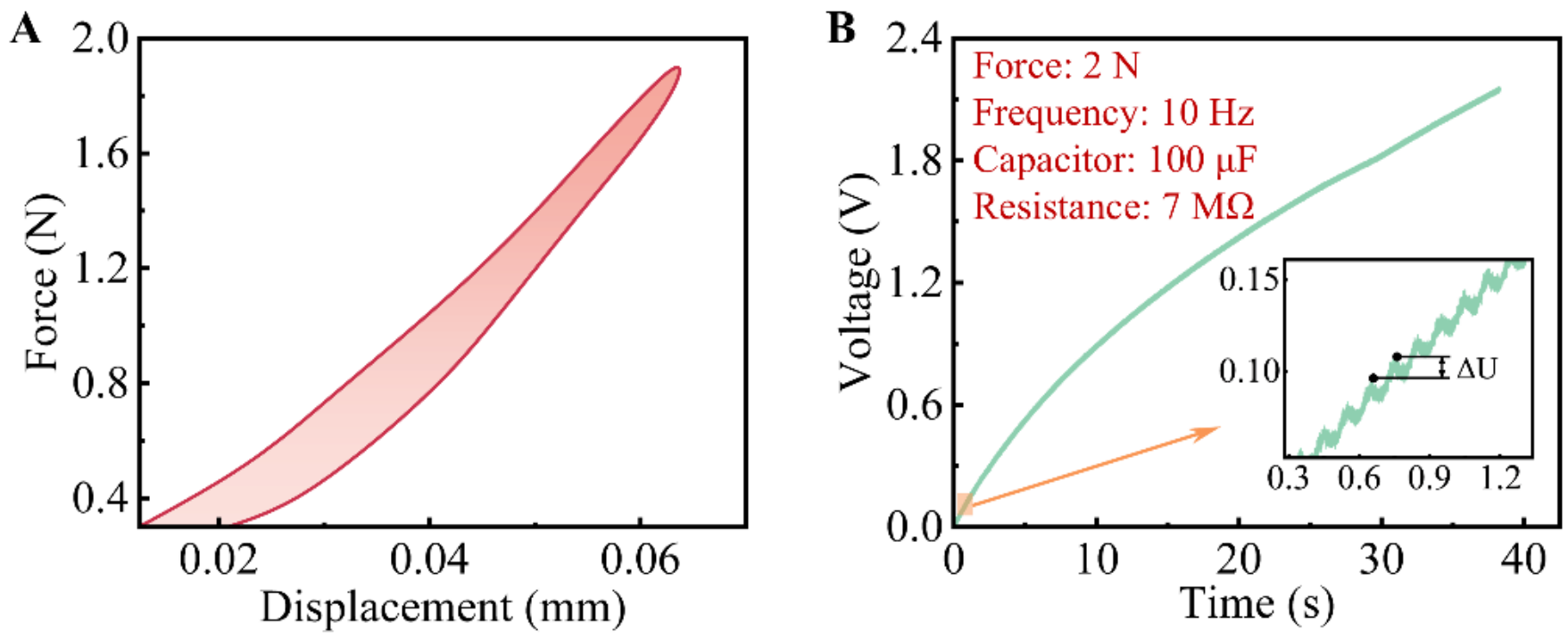

Fig. S28.

(A) Force-displacement curve for calculating the input mechanical energy. **(B)** Voltage changes of the capacitor charged by the k-CAH protection system, for calculating the output electrical energy.

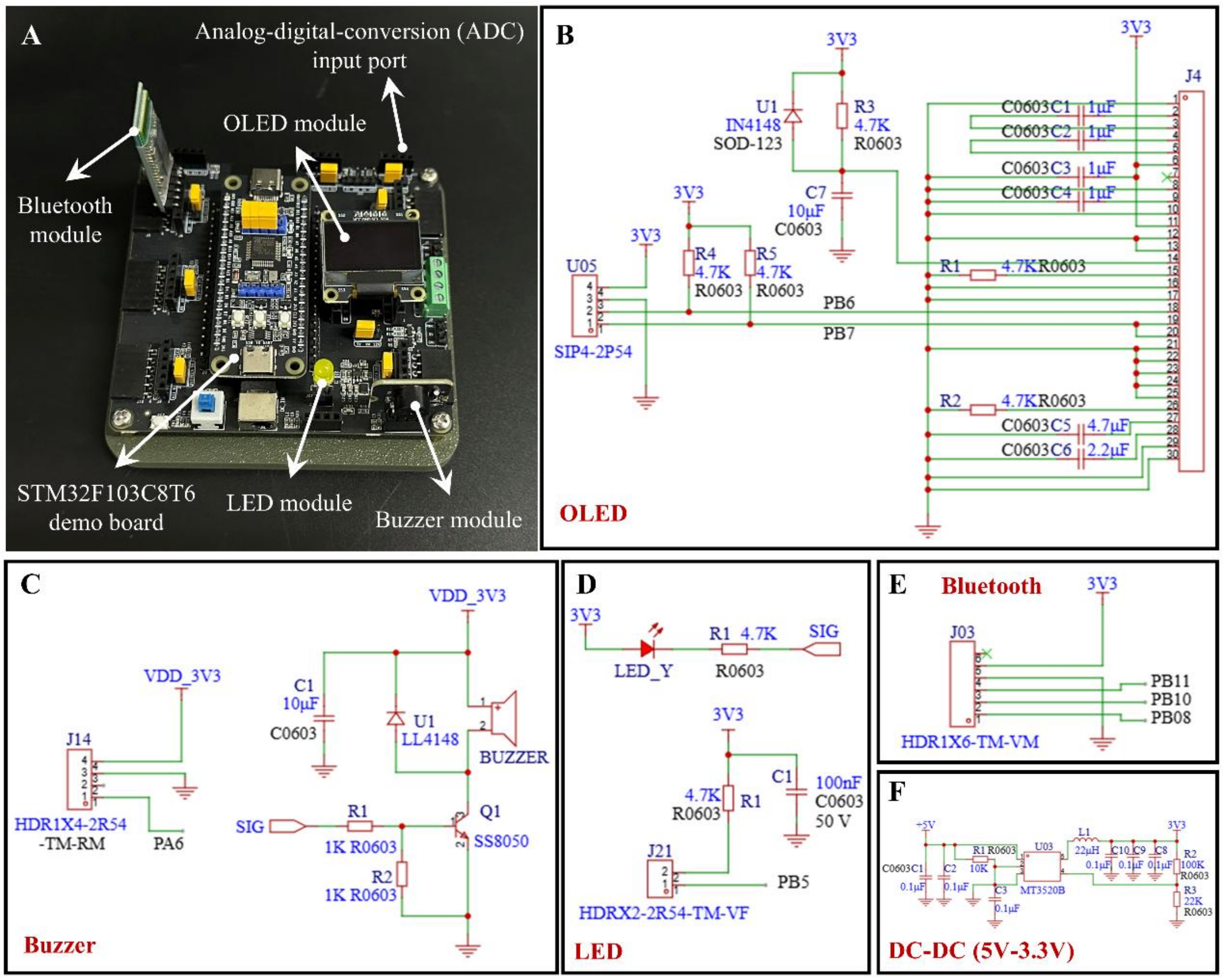

Fig. S29.

(A) STM32F103C8T6 microcomputer (MCU) demo board integrated the alarm system. The circuit diagram of alarming system devices, including **(B)** OLED, **(C)** Buzzer, **(D)** LED, **(E)** Bluetooth and **(F)** voltage conversion (5V DC to 3.3 V DC) modules.

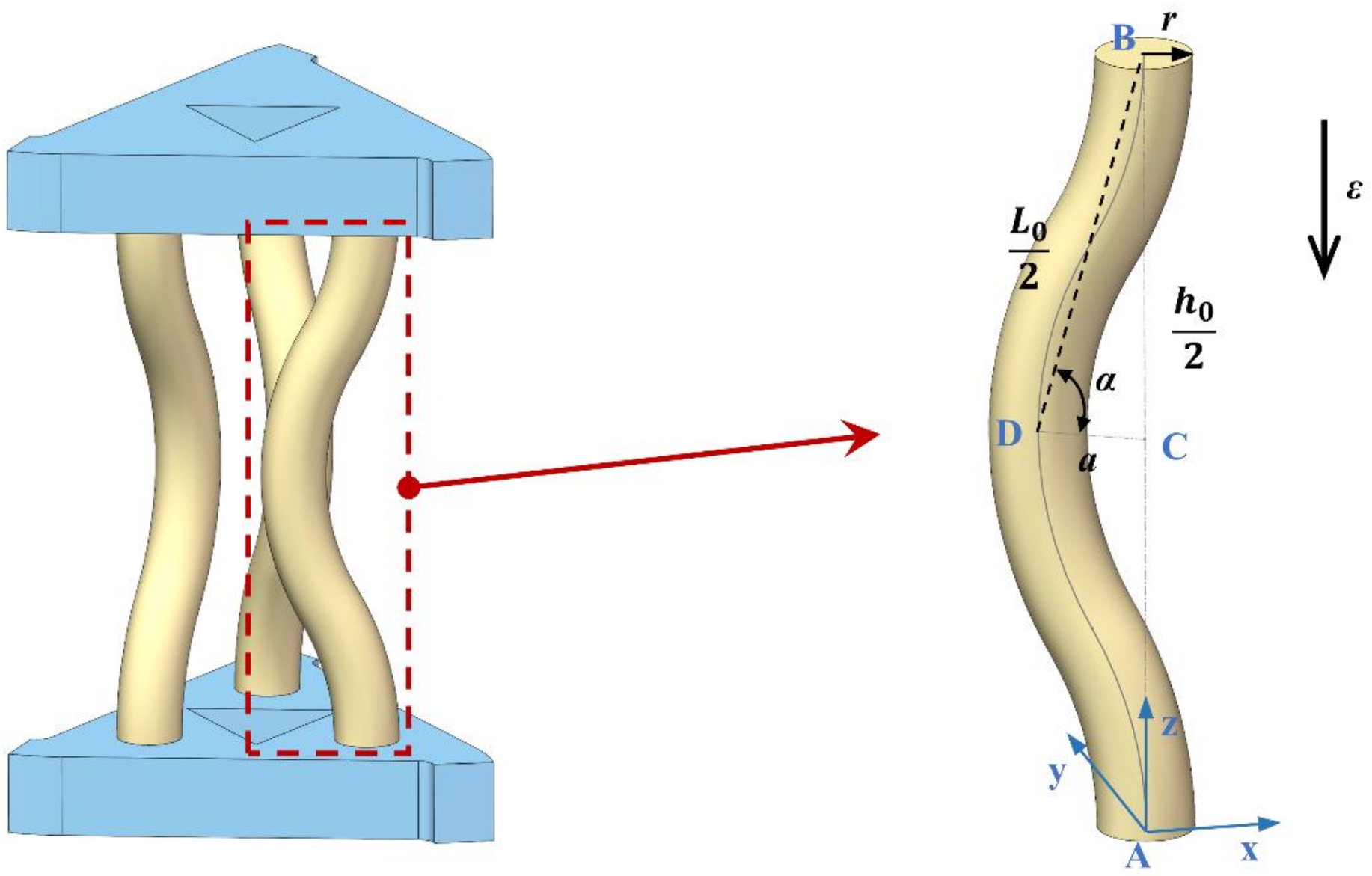

Fig. S30.

Structural diagram and parameter definition of arch metacell.

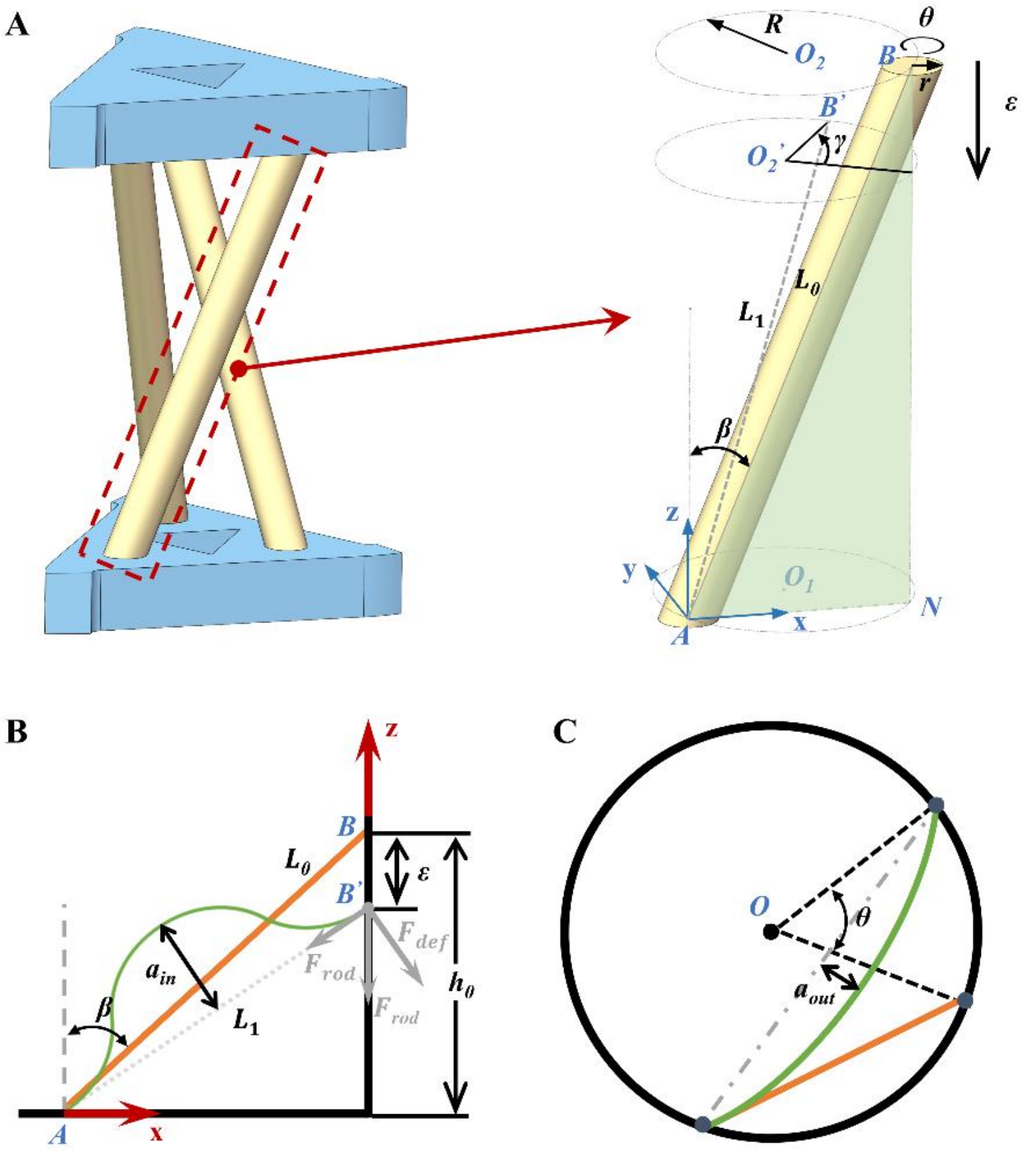

Fig. S31.

(A) Structural diagram and parameter definition of chiral metacell. Deformation of oblique rods at **(B)** vertical ABN surface and **(C)** lateral AO_1N surface.

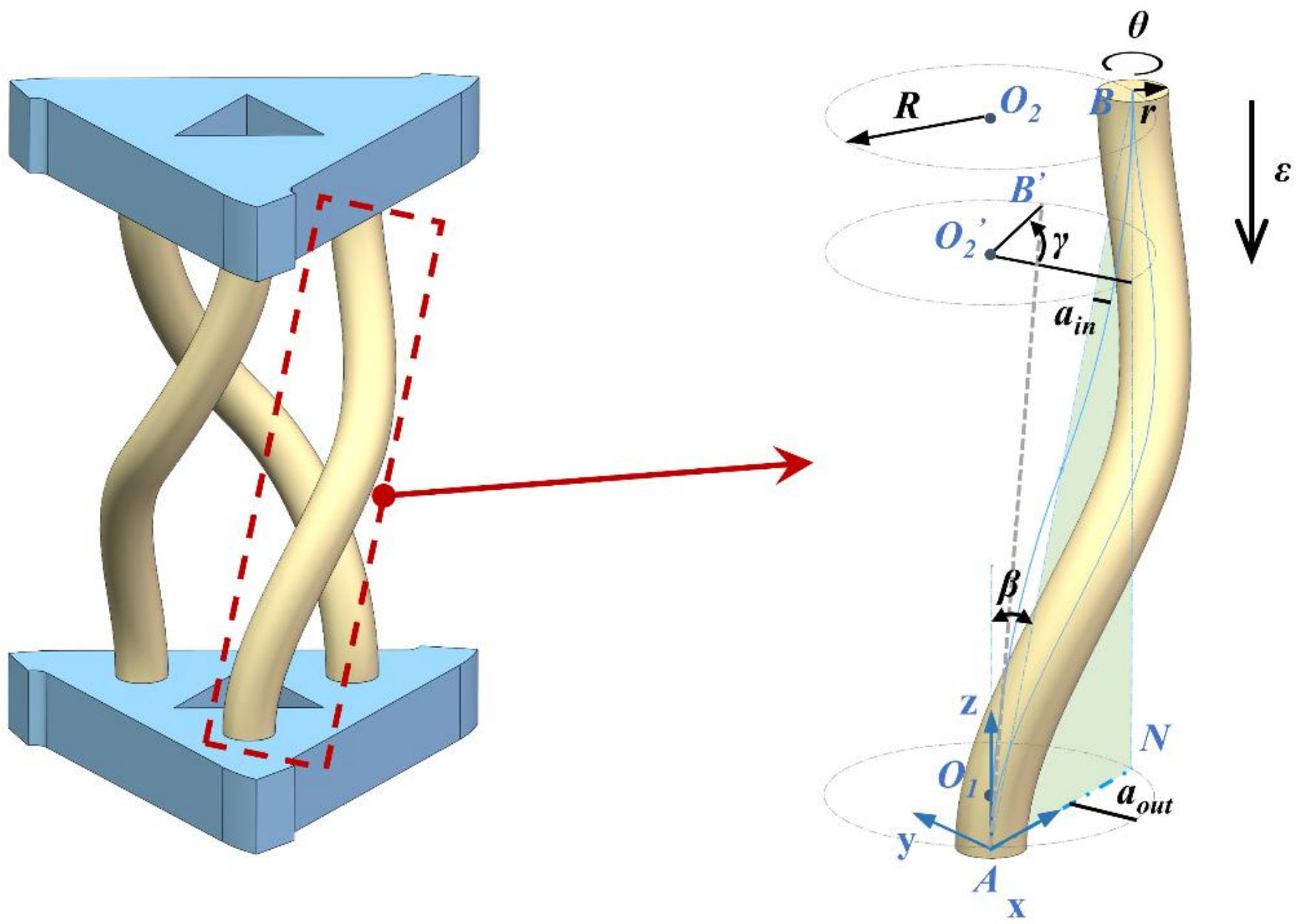

Fig. S32.

Structural diagram and parameter definition of helix chiral arch metacell.

Supplementary Tables

Table S1.

Natural frequency of k-CAH with different topological structure.

**Order**	**Natural frequency (Hz)**		
	k2-CAH	k4-CAH	k6-CAH
1	652.92	670.94	666.49
2	653.00	670.97	666.54
3	835.11	855.71	839.25
4	913.70	898.12	850.26
5	1072.30	974.78	852.91
6	1072.60	974.80	853.01

Table S2.

State-of-art summary of energy absorption performances of metamaterials.

**Material**	**Density (g/cm^3)**	**Structure feature**	**Fabrication method**	**SEA (J/g)**	***Ref.***
Photosensitive resin C-UV 9400 A	1.15	Hexaround lattice	Stereolithography (SLA)	0.14	(*77*)
Thermoplastic polyurethane	1.20	Auxetic “Bucklicrytals” lattice	Selective laser sintering (SLS)	0.1	(*78*)
Thermoplastic polyurethane	1.23	Honeycomb	Fused filament fabrication (FFF)	0.11	(*79*)
Polyurethane	1.20	Octagonal structure	Additive manufacturing technique	0.15	(*80*)
Formlabs® Tough 2000 resin	1.23	Natural-fiber-inspired lattice	SLA	0.15	(*81*)
PLUG-BM	1.17	k-CAH	Digital light processing (DLP)	0.18	**This work**

Table S3.
Comparison on η against state-of-the-art piezoelectric devices

Device type	Operational Bandwidth	Energy conversion efficiency	Sensitivity
Piezoelectric resonant harvester (*82, 83*)	**Extremely Narrow** (Strictly tied to resonant peak)	**High at resonance** (higher than 40%, but severely drops off-resonance)	**Low** (Insensitive to non-resonant input)
Piezoceramic transducers (*84, 85*)	**Moderate** (Structure dependent, lower than 100 Hz or higher than MHz band)	**Moderate** (1.55%-1.79%)	**Moderate to low** (Insensitive to gentle forces)
Flexible piezoelectric sensors (*86-88*)	**Moderate to Broad** (0.1-10 Hz)	**Extremely low** (lower than 0.2%)	**Extremely high** (220-320 mV/kPa)
k-CAH (**this work**)	**Broad** (0.1-60 Hz, far-off-resonant)	**1.35%** (at 10 Hz, far-off-resonant)	**High** (224.3 mV/kPa)

Captions for Supplementary Movies

Movie S1

Mechanical response of CAH metacell during compression process

Movie S2

Deformation behavior revolution of k-CAH metamaterial during unidirectional compression

Movie S3

Mechanical buffering capacity of k-CAH metamaterial protection structure

Movie S4

Energy harvesting capacity of piezoelectric k-CAH metamaterial

Movie S5

Real-time voltage output of piezoelectric k-CAH metamaterial under impact

Movie S6

Real-time impact monitoring and alarming of smart protection system for intelligent equipment

REFERENCES

1. C. Chen, P. Shi, Z. Liu, S. Duan, M. Si, C. Zhang, Y. Du, Y. Yan, T. J. White, R. Kramer-Bottiglio, M. Sitti, T. Iwasaki, X. He, Advancing physical intelligence for autonomous soft robots. *Sci. Robot.* **10**, eads1292 (2025).

2. Y. Zhao, Y. Hong, Y. Li, F. Qi, H. Qing, H. Su, J. Yin, Physically intelligent autonomous soft robotic maze escaper. *Sci. Adv.* **9**, eadi3254 (2023).

3. V. Viswanathan, A. H. Epstein, Y.-M. Chiang, E. Takeuchi, M. Bradley, J. Langford, M. Winter, The challenges and opportunities of battery-powered flight. *Nature* **601**, 519–525 (2022).

4. J. Chen, J. Sun, G. Wang, From unmanned systems to autonomous intelligent systems. *Engineering* **12**, 16–19 (2022).

5. R. T. Scott, L. M. Sanders, E. L. Antonsen, J. J. A. Hastings, S.-M. Park, G. Mackintosh, R. J. Reynolds, A. L. Hoarfrost, A. Sawyer, C. S. Greene, B. S. Glicksberg, C. A. Theriot, D. C. Berrios, J. Miller, J. Babdor, R. Barker, S. E. Baranzini, A. Beheshti, S. Chalk, G. M. Delgado-Aparicio, M. Haendel, A. A. Hamid, P. Heller, D. Jamieson, K. J. Jarvis, J. Kalantari, K. Khezeli, S. V. Komarova, M. Komorowski, P. Kothiyal, A. Mahabal, U. Manor, H. Garcia Martin, C. E. Mason, M. Matar, G. I. Mias, J. G. Myers, C. Nelson, J. Oribello, P. Parsons-Wingerter, R. K. Prabhu, A. A. Qutub, J. Rask, A. Saravia-Butler, S. Saria, N. K. Singh, M. Snyder, F. Soboczenski, K. Soman, D. Van Valen, K. Venkateswaran, L. Warren, L. Worthey, J. H. Yang, M. Zitnik, S. V. Costes, Biomonitoring and precision health in deep space supported by artificial intelligence. *Nat. Mach. Intell.* **5**, 196–207 (2023).

6. Z. Yi, S. Gong, F. Fang, X. Li, J. Zhou, X. Wei, X. Fang, L. Shao, W. Zhang, Sensor-actuator integration for intelligent devices. *Device* **3**, 100717 (2025).

7. X. Wang, R. Wei, Z. Chen, H. Pang, H. Li, Y. Yang, Q. Hua, G. Shen, Bioinspired intelligent soft robotics: From multidisciplinary integration to next-generation intelligence. *Adv. Sci.* **12**, e06296 (2025).

8. Q.-F. Guan, H.-B. Yang, Z.-M. Han, L.-C. Zhou, Y.-B. Zhu, Z.-C. Ling, H.-B. Jiang, P.-F. Wang, T. Ma, H.-A. Wu, S.-H. Yu, Lightweight, tough, and sustainable cellulose nanofiber-derived bulk structural materials with low thermal expansion coefficient. *Sci. Adv.* **6**, eaaz1114 (2020).

9. D. Nepal, S. Kang, K. M. Adstedt, K. Kanhaiya, M. R. Bockstaller, L. C. Brinson, M. J. Buehler, P. V. Coveney, K. Dayal, J. A. El-Awady, L. C. Henderson, D. L. Kaplan, S. Keten, N. A. Kotov, G. C. Schatz, S. Vignolini, F. Vollrath, Y. Wang, B. I. Yakobson, V. V. Tsukruk, H. Heinz, Hierarchically structured bioinspired nanocomposites. *Nat. Mater.* **22**, 18–35 (2023).

10. R. O. Ritchie, The conflicts between strength and toughness. *Nat. Mater.* **10**, 817–822 (2011).

11. U. G. K. Wegst, H. Bai, E. Saiz, A. P. Tomsia, R. O. Ritchie, Bioinspired structural materials. *Nat. Mater.* **14**, 23–36 (2015).

12. C. E. Gregg, D. Catanoso, O. I. B. Formoso, I. Kostitsyna, M. E. Ochalek, T. J. Olatunde, I. W. Park, F. M. Sebastianelli, E. M. Taylor, G. T. Trinh, K. C. Cheung, Ultralight, strong, and self-reprogrammable mechanical metamaterials. *Sci. Robot.* **9**, eadi2746 (2024).

13. M. Utzeri, H. Cebeci, S. Kumar, Autonomous sensing architected materials. *Adv. Funct. Mater.* **35**, 2411975 (2025).

14. L. Wang, R.-W. Li, A more biofriendly piezoelectric material. *Science* **383**, 1416–1416 (2024).

15. Z. Zhang, X. Li, Z. Peng, X. Yan, S. Liu, Y. Hong, Y. Shan, X. Xu, L. Jin, B. Liu, X. Zhang, Y. Chai, S. Zhang, A. K. Y. Jen, Z. Yang, Active self-assembly of piezoelectric biomolecular films via synergistic nanoconfinement and in-situ poling. *Nat. Commun.* **14**, 4094 (2023).

16. H. Cui, D. Yao, R. Hensleigh, H. Lu, A. Calderon, Z. Xu, S. Davaria, Z. Wang, P. Mercier, P. Tarazaga, X. Zheng, Design and printing of proprioceptive three-dimensional architected robotic metamaterials. *Science* **376**, 1287–1293 (2022).

17. Y. Li, G. Wang, S. Zhang, Y. Zhou, H. Li, Z. Qi, Design and calibration of spoke piezoelectric six-dimensional force/torque sensor for space manipulator. *Chin. J. Aeronaut.* **37**, 218–235 (2024).

18. M. Hassanpour Amiri, R. Fatscher, R. Taylor, P. R. F. Rocha, C. R. Bowen, K. Asadi, Piezoelectric energy harvesters: A critical assessment and a standardized reporting of power-producing vibrational harvesters. *Nano Energy* **106**, 108073 (2023).

19. F. Li, Breaking symmetry for piezoelectricity. *Science* **375**, 618–619 (2022).

20. H. Yin, Y. Li, Z. Tian, Q. Li, C. Jiang, E. Liang, Y. Guo, Ultra-high sensitivity anisotropic piezoelectric sensors for structural health monitoring and robotic perception. *Nanomicro Lett.* **17**, 42 (2024).

21. J. Zhang, C. Liu, J. Li, T. Yu, J. Ruan, F. Yang, Advanced piezoelectric materials, devices, and systems for orthopedic medicine. *Adv. Sci.* **12**, e2410400 (2025).

22. Q. Xu, Y. Tao, Z. Wang, H. Zeng, J. Yang, Y. Li, S. Zhao, P. Tang, J. Zhang, M. Yan, Q. Wang, K. Zhou, D. Zhang, H. Xie, Y. Zhang, C. Bowen, Highly flexible, high-performance, and stretchable piezoelectric sensor based on a hierarchical droplet-shaped ceramics with enhanced damage tolerance. *Adv. Mater.* **36**, e2311624 (2024).

23. J. Y. Kim, W. Hwang, S. Y. Han, Y. S. Jung, F. Pang, W. Shen, C. Park, S.-W. Kim, A. Soon, Y. S. Cho, Oxygen-doped 2D In_2Se_3 nanosheets with extended in-plane lattice strain for highly efficient piezoelectric energy harvesting. *Adv. Sci.* **12**, e2410851 (2025).

24. S. I. Kundalwal, V. Choyal, Enhancing the piezoelectric properties of boron nitride nanotubes through defect engineering. *Physica E Low Dimens. Syst. Nanostruct.* **125**, 114304 (2021).

25. I. Terzic, N. L. Meereboer, M. Acuautla, G. Portale, K. Loos, Tailored self-assembled ferroelectric polymer nanostructures with tunable response. *Macromolecules* **52**, 354–364 (2019).

26. L. Wang, G. Ulliac, B. Wang, J. A. Iglesias Martínez, K. K. Dudek, V. Laude, M. Kadic, 3D auxetic metamaterials with elastically-stable continuous phase transition *Adv. Sci.* **9**, 2204721 (2022).

27. G. Librandi, E. Tubaldi, K. Bertoldi, Programming nonreciprocity and reversibility in multistable mechanical metamaterials. *Nat. Commun.* **12**, 3454 (2021).

28. M. Oudich, N. J. R. K. Gerard, Y. Deng, Y. Jing, Tailoring structure-borne sound through bandgap engineering in phononic crystals and metamaterials: A comprehensive review. *Adv. Funct. Mater.* **33**, 2206309 (2023).

29. X. Zheng, Y. Jiang, M. Mete, J. Li, I. Watanabe, T. Yamada, J. Paik, Metamaterial robotics. *Sci. Robot.* **10**, eadx1519 (2025).

30. X. Huang, W. Guo, S. Liu, Y. Li, Y. Qiu, H. Fang, G. Yang, K. Zhu, Z. Yin, Z. Li, H. Wu, Flexible mechanical metamaterials enabled electronic skin for real-time detection of unstable grasping in robotic manipulation. *Adv. Funct. Mater.* **32**, 2109109 (2022).

31. J. U. Surjadi, C. M. Portela, Enabling three-dimensional architected materials across length scales and timescales. *Nat. Mater.* **24**, 493–505 (2025).

32. K. Guo, Y. Li, S. Zhu, C. Li, Y. Lu, Functional nano-architected mechanical metamaterials and devices. *NPJ Metamater.* **2**, 1 (2026).

33. X. He, G. Li, L. Zhang, Y. Huang, B. Xie, Z. Shi, G. Feng, W. Liu, F. Lyu, S. Wang, Z. Yu, J. Luan, C. Zhao, H. Lu, X. Hu, Q. Zhu, J. Lu, Bio-inspired material-structure-function integrated additive manufacturing of Al-based metamaterials with surpassing energy absorption. *Sci. Adv.* **11**, eaea0430 (2025).

34. J. U. Surjadi, L. Wang, S. Qu, B. F. G. Aymon, J. Ding, X. Zhou, R. Fan, H. Yang, Q. Zhao, X. Song, Y. Lu, Exploiting multiscale dynamic toughening in multicomponent alloy metamaterials for extreme impact mitigation. *Sci. Adv.* **11**, eadt0589 (2025).

35. B. Song, S. Zhang, L. Zhang, Y. Shi, Development trends and challenges of additive manufacturing metamaterials. *Engineering* **44**, 2–6 (2025).

36. H. Cui, R. Hensleigh, D. Yao, D. Maurya, P. Kumar, M. G. Kang, S. Priya, X. Zheng, Three-dimensional printing of piezoelectric materials with designed anisotropy and directional response. *Nat. Mater.* **18**, 234–241 (2019).

37. Z. Wang, J. Cheng, Y. Xie, Y. Wang, Z. Yu, S. Li, L. Li, S. Dong, H. Wang, Lead-free piezoelectric composite based on a metamaterial for electromechanical energy conversion. *Adv. Mater. Technol.* **7**, 2200650 (2022).

38. P. Wang, Q. Tang, L. Zhang, M. Xu, L. Sun, S. Sun, J. Zhang, S. Wang, X. Liang, Ultrasmall barium titanate nanoparticles for highly efficient hypoxic tumor therapy via ultrasound triggered piezocatalysis and water splitting. *ACS Nano* **15**, 11326–11340 (2021).

39. S. Li, Y. Wang, Z. Liu, B. Chen, M. Liu, X. He, S. Yang, Flexible pyroelectric energy harvesters from nanocomposites of liquid crystal elastomers/lead zirconate titanate nanoparticles. *Sci. Adv.* **11**, eadt6136 (2025).

40. G. Tan, L. Zhou, C. Ning, Y. Tan, G. Ni, J. Liao, P. Yu, X. Chen, Biomimetically-mineralized composite coatings on titanium functionalized with gelatin methacrylate hydrogels. *Appl. Surf. Sci.* **279**, 293–299 (2013).

41. S. Ge, S. Wang, W. Mai, K. Zhang, M. Tanveer, L. Wang, C. Tian, Characteristics and acidic soil amelioration effects of biochar derived from a typical halophyte Salicornia europaea L. (common glasswort). *Environ. Sci. Pollut. Res.* **30**, 66113–66124 (2023).

42. C. Murugan, H. Lee, S. Park, Tumor-targeted molybdenum disulfide@ barium titanate core–shell nanomedicine for dual photothermal and chemotherapy of triple-negative breast cancer cells. *J. Mater. Chem. B* **11**, 1044–1056 (2023).

43. G. Li, X. Chen, H. Guo, L. Liu, S. Li, Y. Zhu, Y. Wei, Insulation properties of polypropylene and silicone rubber modified by barium strontium titanate and interfacial charge accumulation properties. *Compos. Sci. Technol.* **261**, 111037 (2025).

44. H. Zheng, J. Chen, M. Que, T. Yang, Z. Liu, W. Cai, L. Yang, X. Liu, Y. Li, X. Yang, Y. Ma, G. Zhu, Highly efficient piezoelectric field enhanced photocatalytic performance via in situ

formation of $BaTiO_3$ on $Ti_3C_2T_x$ for phenolic compound degradation. *Inorg. Chem. Front.* **9**, 4201–4215 (2022).

45. L. Persano, A. Camposeo, F. Matino, R. Wang, T. Natarajan, Q. Li, M. Pan, Y. Su, S. Kar-Narayan, F. Auricchio, G. Scalet, C. Bowen, X. Wang, D. Pisignano, Advanced materials for energy harvesting and soft robotics: Emerging frontiers to enhance piezoelectric performance and functionality. *Adv. Mater.* **36**, e2405363 (2024).

46. M. Li, H. J. Wondergem, M.-J. Spijkman, K. Asadi, I. Katsouras, P. W. M. Blom, D. M. de Leeuw, Revisiting the δ-phase of poly(vinylidene fluoride) for solution-processed ferroelectric thin films. *Nat. Mater.* **12**, 433–438 (2013).

47. G. Zhao, B. Huang, J. Zhang, A. Wang, K. Ren, Z. L. Wang, Electrospun poly(l-lactic acid) nanofibers for nanogenerator and diagnostic sensor applications. *Macromol. Mater. Eng.* **302**, 1600476 (2017).

48. L. Meng, L. Zhou, C. Liu, H. Jia, Y. Lu, D. Ji, T. Liang, Y. Yuan, X. Zhang, Y. Zhu, Y. Jiang, P. Guan, Y. Zhou, Q. Zhang, T. Wan, M. Li, Z. Li, R. Joshi, Z. Han, D. Chu, Synergistic barium titanate/MXene composite as a high-performance piezo-photocatalyst for efficient dye degradation. *J. Colloid Interface Sci.* **674**, 972–981 (2024).

49. Z. Yang, W. Xia, L. Pan, X. Zhang, C. Wang, Mechanical clamping by inverse piezoelectric effect to improve the electromechanical coupling effect of a 1-3 PZT/P (VDF-TrFE) composite sheet. *J. Mater. Sci. Mater. Electron.* **35**, 944 (2024).

50. T. Tang, Z. Shen, J. Wang, S. Xu, J. Jiang, J. Chang, M. Guo, Y. Fan, Y. Xiao, Z. Dong, H. Huang, X. Li, Y. Zhang, D. Wang, L.-Q. Chen, K. Wang, S. Zhang, C.-W. Nan, Y. Shen, Stretchable polymer composites with ultrahigh piezoelectric performance. *Natl. Sci. Rev.* **10**, nwad177 (2023).

51. H. Fazeli, E. Izadi Zaman Abadi, S. Jalil Hoseini, Experimental and numerical study of the effect of steel Tie-rods on load-bearing capacity of Persian brick arches. *Structures* **48**, 64–78 (2023).

52. X. Fang, D. Yu, J. Wen, Y. Dai, M. R. Begley, H. Gao, P. Gumbsch, Large recoverable elastic energy in chiral metamaterials via twist buckling. *Nature* **639**, 639–645 (2025).

53. M. Kadic, G. W. Milton, M. van Hecke, M. Wegener, 3D metamaterials. *Nat. Rev. Phys.* **1**, 198–210 (2019).

54. S. Stark, M. R. Begley, R. M. McMeeking, The buckling and postbuckling of fibrils adhering to a rigid surface. *J. Appl. Mech.* **80**, 041022 (2013).

55. S. Sivaj, S. K. Saha, S. P. Singh, Deformation analysis of cosserat rods using piecewise clothoid approximation. *Int. J. Numer. Methods Eng.* **126**, e7608 (2025).

56. Z. Zhang, J. Dai, C. Wang, H. Zhu, F. Pang, Z. Cheng, W. Ji, 2D kagome materials: Theoretical insights, experimental realizations, and electronic structures. *Adv. Funct. Mater.* **35**, 2416508 (2025).

57. N. J. Ghimire, I. I. Mazin, Topology and correlations on the kagome lattice. *Nat. Mater.* **19**, 137–138 (2020).

58. G. Wu, Y. Cho, I.-S. Choi, D. Ge, J. Li, H. N. Han, T. Lubensky, S. Yang, Directing the deformation paths of soft metamaterials with prescribed asymmetric units. *Adv. Mater.* **27**, 2747–2752 (2015).

59. K. Wei, Q. Yang, B. Ling, H. Xie, Z. Qu, D. Fang, Mechanical responses of titanium 3D kagome lattice structure manufactured by selective laser melting. *Extreme Mech. Lett.* **23**, 41–48 (2018).

60. K. H. Matlack, A. Bauhofer, S. Krödel, A. Palermo, C. Daraio, Composite 3D-printed metastructures for low-frequency and broadband vibration absorption. *Proc. Natl. Acad. Sci. U.S.A.* **113**, 8386–8390 (2016).

61. L. Li, L. Wang, L. Yuan, R. Zheng, Y. Wu, J. Sui, J. Zhong, Micro-vibration suppression methods and key technologies for high-precision space optical instruments. *Acta Astronaut.* **180**, 417–428 (2021).

62. Z. Zhang, Q. Li, Z. Zhang, L. Liu, W. Li, Integrated modeling and active suppression of multi-source micro-vibrations in aerospace vehicles. *Adv. Space Res.* **77**, 3670–3683 (2026).

63. S. A. Kouritem, M. A. Bani-Hani, M. Beshir, M. M. Y. B. Elshabasy, W. A. Altabey, Automatic resonance tuning technique for an ultra-broadband piezoelectric energy harvester. *Energies* **15**, 8386–8390 (2022).

64. C. Sun, D. Li, W. Liao, T. Liu, Rigid-flexible interlocked metastructures enable conformal stealth. *Sci. Adv.* **11**, eaeb7870 (2025).

65. S. Wang, A. A. Khan, S. Teale, J. Xu, D. H. Parmar, R. Zhao, L. Grater, P. Serles, Y. Zou, T. Filleter, D. S. Seferos, D. Ban, E. H. Sargent, Large piezoelectric response in a Jahn-Teller distorted molecular metal halide. *Nat. Commun.* **14**, 1852 (2023).

66. J. Li, Y. Yang, H. Jiang, Y. Wang, Y. Chen, S. Jiang, J.-M. Wu, G. Zhang, 3D interpenetrating piezoceramic-polymer composites with high damping and piezoelectricity for impact energy-absorbing and perception. *Compos. Part B Eng.* **232**, 109617 (2022).

67. H. Kim, F. Torres, D. Villagran, C. Stewart, Y. Lin, T.-L. B. Tseng, 3D Printing of $BaTiO_3$/PVDF composites with electric in situ poling for pressure sensor applications. *Macromol. Mater. Eng.* **302**, 1700229 (2017).

68. D. Zhang, Y. Li, J. Hu, Z. Gong, J. Liu, J. Cao, P. Dong, H. Yuan, H. Wang, Flexible PZT-resin auxetic Metamaterials: Design, fabrication, and performance evaluation. *Ceram. Int.* **51**, 38110–38128 (2025).

69. L. He, H. Kurita, Z. Zhao, F. Narita, Multistable piezoelectric mechanical metamaterials for integrated energy absorption and energy harvesting. *Compos. Struct.* **377**, 119871 (2026).

70. L. Song, R. Dai, Y. Li, Q. Wang, C. Zhang, Polyvinylidene fluoride energy harvester with boosting piezoelectric performance through 3D printed biomimetic bone structures. *ACS Sustain. Chem. Eng.* **9**, 7561–7568 (2021).

71. M. Habib, I. Lantgios, K. Hornbostel, A review of ceramic, polymer and composite piezoelectric materials. *J. Phys. D Appl. Phys.* **55**, 423002 (2022).

72. Y. Hong, S. Liu, X. Yang, W. Hong, Y. Shan, B. Wang, Z. Zhang, X. Yan, W. Lin, X. Li, Z. Peng, X. Xu, Z. Yang, A bioinspired surface tension-driven route toward programmed cellular ceramics. *Nat. Commun.* **15**, 5030 (2024).

73. R. Liu, K. H. Shin, Y. Zhu, Q. Liu, B. Ji, G. Sun, Z. Li, D. De Silva, A. Stewart, M. Lorenzoni, I. Ludtke, O. A. Williams, W. Ming, G. Divitini, J. I. Sohn, B. Hou, Modulating D_{33} coefficients through in situ AgF and Ag_2O growth in PVDF composites for high-performance piezoelectric nanogenerators. *Adv. Mater. Technol.* **10**, 2500012 (2025).

74. A. Megdich, M. Habibi, L. Laperrière, Modeling and optimization of piezoelectric and dielectric properties of poled PVDF/BT nanocomposites. *Polym. Bull.* **81**, 9181–9201 (2024).

75. Z. Wang, Y. Wang, C. Yang, T. Zheng, R. Luo, Y. Wang, Applications of piezoelectric materials in biomedical engineering. *Macromol. Biosci.* **25**, 2500033 (2025).

76. J. Hua, Y. Zhou, Z. Meng, C. Q. Chen, Pre-compressed beam-based multistable mechanical metamaterials with programmable loading and unloading deformation sequences. *Thin-Walled Struct.* **209**, 112879 (2025).

77. S. Li, Y. Hou, J. Huang, J. Shi, L. Meng, Exploring the enhanced energy-absorption performance of hybrid polyurethane(PU)-foam-filled lattice metamaterials. *Int. J. Impact Eng.* **193**, 105058 (2024).

78. S. Yuan, F. Shen, J. Bai, C. K. Chua, J. Wei, K. Zhou, 3D soft auxetic lattice structures fabricated by selective laser sintering: TPU powder evaluation and process optimization. *Mater. Des.* **120**, 317–327 (2017).

79. S. R. G. Bates, I. R. Farrow, R. S. Trask, 3D printed polyurethane honeycombs for repeated tailored energy absorption. *Mater. Des.* **112**, 172–183 (2016).

80. R. Hamzehei, J. Kadkhodapour, A. P. Anaraki, S. Rezaei, S. Dariushi, A. M. Rezadoust, Octagonal auxetic metamaterials with hyperelastic properties for large compressive deformation. *Int. J. Mech. Sci.* **145**, 96–105 (2018).

81. Z. Gao, H. Wang, N. Letov, Y. F. Zhao, X. Zhang, Y. Wu, C. L. A. Leung, H. Wang, Data-driven design of biometric composite metamaterials with extremely recoverable and ultrahigh specific energy absorption. *Compos. Part B Eng.* **251**, 110468 (2023).

82. Y.-C. Shu, I. Lien, Efficiency of energy conversion for a piezoelectric power harvesting system. *J. Micromech. Microeng.* **16**, 2429–2438 (2006).

83. L. Tang, Y. Yang, C. K. Soh, Toward broadband vibration-based energy harvesting. *J. Intell. Mater. Syst. Struct.* **21**, 1867–1897 (2010).

84. H. Lu, H. Cui, G. Lu, L. Jiang, R. Hensleigh, Y. Zeng, A. Rayes, M. K. Panduranga, M. Acharya, Z. Wang, A. Irimia, F. Wu, G. P. Carman, J. M. Morales, S. Putterman, L. W. Martin, Q. Zhou, X. Zheng, 3D Printing and processing of miniaturized transducers with near-pristine piezoelectric ceramics for localized cavitation. *Nat. Commun.* **14**, 2418 (2023).

85. R. Koven, M. Mills, R. Gale, B. Aksak, Low-frequency and broadband vibration energy harvesting using base-mounted piezoelectric transducers. *IEEE Trans. Ultrason. Ferroelectr. Freq. Control* **64**, 1735–1743 (2017).

86. F. Yang, H. Yang, G. Zhang, S. Xu, L. Zhu, X. Gong, L. Liu, F. Luo, S. Xu, C. Liu, J. Wu, S. Jiang, K. Li, L. Dong, X. Chen, S. Zhang, Y. Zhang, Q. Wang, Mechanoelectrical metamaterials for broad-range, high-sensitivity pressure sensing. *Science* **392**, 1177–1182 (2026).

87. J.-Q. Luo, H.-F. Lu, Y.-J. Nie, Y.-H. Zhou, C.-F. Wang, Z.-X. Zhang, D.-W. Fu, Y. Zhang, Porous flexible molecular-based piezoelectric composite achieves milliwatt output power density. *Nat. Commun.* **15**, 8636 (2024).

88. C. Bowen, H. A. Kim, P. Weaver, S. Dunn, Piezoelectric and ferroelectric materials and structures for energy harvesting applications. *Energ. Environ. Sci.* **7**, 25–44 (2014).
